# Reprogrammed Cells Display Distinct Proteomic Signatures Associated with Colony Morphology Variability

**DOI:** 10.1155/2019/8036035

**Published:** 2019-11-18

**Authors:** Yngvild Bjørlykke, Anne M. Søviknes, Laurence Hoareau, Heidrun Vethe, Andreas F. Mathisen, Simona Chera, Marc Vaudel, Luiza M. Ghila, Helge Ræder

**Affiliations:** ^1^Department of Clinical Science, University of Bergen, Bergen, Norway; ^2^Department of Pediatrics, Haukeland University Hospital, Bergen, Norway

## Abstract

Human induced pluripotent stem cells (hiPSCs) are of high interest because they can be differentiated into a vast range of different cell types. Ideally, reprogrammed cells should sustain long-term culturing in an undifferentiated state. However, some reprogrammed cell lines represent an unstable state by spontaneously differentiating and changing their cellular phenotype and colony morphology. This phenomenon is not fully understood, and no method is available to predict it reliably. In this study, we analyzed and compared the proteome landscape of 20 reprogrammed cell lines classified as stable and unstable based on long-term colony morphology. We identified distinct proteomic signatures associated with stable colony morphology and with unstable colony morphology, although the typical pluripotency markers (POU5F1, SOX2) were present with both morphologies. Notably, epithelial to mesenchymal transition (EMT) protein markers were associated with unstable colony morphology, and the transforming growth factor beta (TGFB) signalling pathway was predicted as one of the main regulator pathways involved in this process. Furthermore, we identified specific proteins that separated the stable from the unstable state. Finally, we assessed both spontaneous embryonic body (EB) formation and directed differentiation and showed that reprogrammed lines with an unstable colony morphology had reduced differentiation capacity. To conclude, we found that different defined patterns of colony morphology in reprogrammed cells were associated with distinct proteomic profiles and different outcomes in differentiation capacity.

## 1. Introduction

Human pluripotent stem cells (hPSCs) such as induced pluripotent stem cells (iPSCs) and embryonic stem cells (ESCs) have the potential to be differentiated into a whole range of different cell types and are, therefore, of high interest for both researchers and clinicians. Reprogramming of somatic cells to generate hiPSCs has rapidly gained popularity as it enables the use of patient-specific cells.

Maintaining cells in a pluripotent state *in vitro* requires routine monitoring during expansion. A typical characterization pipeline to ensure pluripotency includes expression of singular pluripotency markers (SOX2 and POU5F1), karyotype analysis, and the ability to form the three germ layers using teratoma assays or embryoid body formation [1]. Despite these quality controls, numerous studies have shown major line-to-line variations [2–5]. To improve the utility of hPSCs in regenerative medicine and to ensure high-quality clinical-grade cell products, we need a pipeline of robust quality control methods that can be automated to benchmark the cells and filter out reprogrammed cells of inferior quality.

Besides teratoma formation, the colony morphology of reprogrammed cells is considered an important assessment criterion of pluripotency [6–10]. In several studies, the capacity to form teratomas and stable culturing has been correlated to colony morphology [6, 11–13], thus correlating this aspect with the functionality of the hiPSC. However, during long-term culturing, the colony morphology has been observed to vary in basically two forms: stable and unstable colony morphologies. Typically, a reprogrammed cell line with a stable colony morphology exhibits compact colonies, usually round, with distinct borders and well-defined sharp edges and is associated with a pluripotent state [14]. A reprogrammed cell line with an unstable colony morphology exhibits irregular colony morphology and is associated with spontaneous differentiation [9]. Although colony morphology is an important indicator of pluripotency, it suffers from subjective evaluation and lack of well-established quantitative metrics. Several groups have in recent years established metrics of colony morphology based on image acquisition to probe for loss of pluripotency [8, 15]. However, this requires sophisticated microscopy methods and only takes into account the physical characteristics of the cells and colonies.

Proteomics provides an excellent tool for large-scale quantification and benchmarking of cells and an opportunity to further improve the characterization of colony morphology of reprogrammed cells. Compared to other ~omics approaches (transcriptomics and genomics), proteomics measures the translated proteins as opposed to molecules that potentially can become the proteins [16]. The proteome is dynamic and changes rapidly. In this study, we hypothesized that the proteome of reprogrammed cell lines showing stable colony morphology would differ from reprogrammed cell lines showing unstable colony morphology. Subsequently, we aimed to use proteomics to obtain insight into the molecular landscape associated with different colony morphology groups and corresponding variable differentiation potential.

## 2. Materials and Methods

### 2.1. Cell Source

We reprogrammed fibroblasts taken from seven donors. All patients gave written informed consent. The reported experiments were approved by the Regional Committee of Medical and Health Research Ethics (REK 2010/2295). All methods were performed according to the Declaration of Helsinki. A total of 20 reprogrammed cell lines were generated. From donor 1, we generated the following reprogrammed cell lines; 1-A, 1-B, and 1-C. Furthermore, cell lines 2-A, 2-B, and 2-C are derived from donor 2; cell lines 3-A, 3-B, 3-C, and 3-D are derived from donor 3; cell lines 4-A, 4-B, and 4-C are derived from donor 4; cell lines 5-A and 5-B are derived from donor 5; cell lines 6-A, 6-B, and 6-C are derived from donor 6, while cell lines 7-A and 7-B are derived from donor 7. Additional information can be found in supplementary [Supplementary-material supplementary-material-1].

### 2.2. Episomal Reprogramming

Reprogramming of fibroblasts from donors 1-4 was performed by using episomal reprogramming [17] with vectors from Addgene: #27077 (OCT3/4), #27080 (L-MYC, LIN28), and #27078 (SOX2, KLF4). The plasmids were expanded in bacterial culture and purified using the QIAfilter Midi Kit (cat# 12243, Qiagen). The inserted genes were verified with PCR using the primers pCAG-F (GCAACGTGCTGGTTATTGTG) and WPRE-R (CATAGCGTAAAAGGAGCAACA). Fibroblast cells were reprogrammed using the Amaxa NHDF Nucleofector Kit (cat# VAPI-1001, Lonza). Fibroblast cells were harvested with trypsin (0.25%), and 500,000 cells were dissolved in 100 *μ*L Amaxa NHDF Solution together with 1 *μ*g of each plasmid. The plasmids were delivered to the cells by electroporation (Nucleofector™ 2b Device) and plated in MEF media (DMEM, 10% FBS, 1% L-glutamine, 1% minimum essential medium nonessential amino acid (MEM-NEAA), 1% Sodium Pyruvate). At day six, the fibroblasts were split 1 : 6, and from day seven, a knockout serum replacement- (KOSR-) based hESC medium (DMEM-F12, 20% KOSR, 1% MEM-NEAA, 1% GlutaMAX, and 1% Penicillin-Streptomycin (P/S)) was used. Clearance of the episomal plasmids was observed at day 13 after transfection, by disappearance of the GFP-tagged control vector. Media were changed daily until pickable colonies emerged by 21-26 days post transfection. Two to four different colonies per donor were picked and expanded in mTeSR™1 media (cat# 85850, STEMCELL Technologies). All reprogrammed lines were tested negative for mycoplasma.

### 2.3. Sendai Reprogramming

Reprogramming of donors 5-7 was performed by Sendai reprogramming and carried out by the company Takara Bio Inc. using a CytoTune-iPS 2.0 Sendai Reprogramming Kit (cat# A16517, Life Technologies). Clearance of the Sendai virus was tested by Q-PCR using a TaqMan assay for Sendai virus. The Sendai virus level was under the detection limit (CT ≥ 36) for all the generated clones. Colonies were picked 3-4 weeks post transduction and expanded in a Cellartis Feeder-Free DEF-CS Culture System (cat# Y30017, Takara). All hiPSC lines were tested negative for mycoplasma.

### 2.4. Maintenance of the Reprogrammed Cells

The reprogrammed cell lines were cultured in 6-well plates (cat# 83.3920, Sarstedt), coated with Matrigel (cat# 354230, Corning). The cells were maintained in mTeSR™1 media, and media were changed every day. Once the dish was confluent, just before colonies were in contact with each other, the cells were split by using a Gentle Cell Dissociation Reagent (cat# 7174, STEMCELL Technologies) by following the instruction provided by the supplier. In brief, 1 mL Gentle Cell Dissociation Reagent was added to a well in a 6-well plate and incubated (37°C) for 5 min, followed by replacing the Gentle Cell Dissociation Reagent by 1 mL prewarmed mTeSR™1 media and subsequently disrupting the colonies by gently scraping the surface with a cell scraper. The cells were split to a ratio between 1 : 6 and 1 : 10 depending on the growth rate of the line and further cultivated until confluency was reached again.

### 2.5. SSEA4^+^ Enrichment

All reprogrammed cell lines were enriched for Anti-Stage-Specific Embryonic Antigen 4- (SSEA-4-) positive cells by using magnetic cell isolation with MicroBeads (cat# 130097855, Miltenyi Biotec) following the guidelines provided by the supplier.

### 2.6. Classification of Reprogrammed Cell Lines

The reprogrammed cell lines were qualitatively evaluated by using a phase-contrast microscope and manually assigned to one of the three morphology groups (stable colony, unstable class 1, and unstable class 2). Representative lines for each colony morphology group were imaged using a Nikon TE2000 with a 10x objective. Immunocytochemistry analysis was performed on a representative line for each colony morphology group. Cells were cultured on glass coverslips and fixed in 2% PFA for 15 min. The immunofluorescence protocol was performed following the guidelines provided by the suppliers. The following antibodies were used: mouse anti-human *α*-tubulin (1/100, cat# T5168, Sigma), rabbit anti-human *β*-tubulin (1/500, cat# ab32572, Abcam); and the following secondary antibodies were used: donkey anti-rabbit A647 (1/500, Molecular Probes) and donkey anti-mouse A594 (1/500, Molecular Probes). The nuclei were stained with DAPI (cat# D1306, Molecular Probes). The samples were mounted in ProLong Diamond Antifade Mountant Media (cat# P36970, Life Technologies). The expression of *β*-tubulin and *α*-tubulin was analyzed by using a Leica TCS SP5 confocal microscope with a 40x objective. No specific feature of the original data was obscured, eliminated, or misrepresented.

### 2.7. Embryonic Body Formation

Embryonic bodies were generated by following the instructions of the AggreWell™800 Starter Kit (cat# 34850, STEMCELL Technologies). Briefly, cells were harvested with the Gentle Cell Dissociation Reagent and 1.2 million cells were plated in the AggreWell™800 plates and incubated for 24 hours. The generation of embryonic bodies was facilitated by culturing the embryonic bodies in Primate ES Cell Media (cat# 258RCHEMD001, Tebu Bio), the first 10 days in suspension plates (cat# 83.3920.500, Sarstedt) followed by 14 days in 6-well plates (cat# 83.3920, Sarstedt), coated with Matrigel (cat# 354230, Corning). The embryonic bodies were stained for beta-III tubulin (TUJ1), smooth muscle actin (SMA), and alpha-fetoprotein (AFP) by following the instructions of the 3-germ layer immunocytochemistry kit (cat# A25538, Thermo). The expression of TUJ1 (1/500), SMA (1 : 100), and AFP (1 : 500) were analyzed by using a Leica TCS SP2 microscope with a 40x objective, a Leica TCS SP5 confocal with a 40x objective, or a Leica TCS SP8 STED 3X confocal microscope with a 100x objective.

### 2.8. Differentiation Experiments

Cells were directed towards definite endoderm (DE) and primitive gut tube (PG) in MCDB 131 medium (cat# 10372-019, Thermo Fisher Scientific) with 1% 100x GlutaMAX, 1.5 g/L NaHCO_3_, 0.5% BSA, and a 10 mM final glucose concentration. Differentiation to DE was done in 3 days by daily adding 100 ng/mL Activin A (cat# 120-14, PeproTech) and 0.3 *μ*M CHIR-99021 (reduced to 0 on the last day) (cat# S2924, Selleckchem). Further differentiation to PG was done in 2 days by daily adding 0.25 mM ascorbic acid (cat# A4544, Sigma) and 50 ng/mL FGF7 (cat# 100-19, PeproTech). The cells were analyzed by flow cytometry.

### 2.9. Flow Cytometry Analysis

Cells were washed in Ca/Mg-free PBS and incubated with TrypLE™ Select (cat# 12563011, Thermo Fisher Scientific) 5 minutes in the incubator. The cell suspension was washed in Ca/Mg-free PBS and then centrifuged 500 g for 4 minutes. The pellet was resuspended in Ca/Mg-free PBS and incubated with the LIVE/DEAD Fixable Dead Cell Near-IR Fluorescent Dye (cat# L10119, Invitrogen), according to the manufacturer's instructions. Next, the cells were then fixed and permeabilized with the Fix/Perm Solution Kit (cat# 554714, BD Biosciences) according to the manufacturer's instructions. Cells were then stained with antibodies and washed. For CD9 analysis (surface marker), cells were stained with the antibody before fixation. A titration curve was previously done to determine the volume of antibody to add per tube of 10^6^ cells: 1.5 *μ*L of AF488-POU5F1 antibody (cat# BD560253, BD Biosciences), 1 *μ*L of APC-SOX17 antibody (cat# IC1924A, R&D), and 0.2 *μ*L of APC-CD9 antibody (cat# BD341648, BD Biosciences) and the same amount of isotype control antibodies (cat# BD55772, BD Biosciences; cat# IC108A, R&D; cat# IC003R, R&D). Data were analyzed with FlowJo 10. Dead cells, debris, and doublets were excluded, and after compensation, gating was determined on FL1/FL4 dot plots using Fluorescence Minus One (FMO) controls. Unstained cells and isotype controls were run separately.

### 2.10. Global Proteomic Analysis

#### 2.10.1. Sample Preparation with SDS Lysis Buffer and Filter-Aided Sample Preparation (FASP)

Cells were harvested with TrypLE™ Select, washed twice with Ca/Mg-free PBS. The cell pellet was resuspended in lysis buffer (4% SDS, 0.1 M Tris, pH 7.6), boiled for 7 minutes at 95°C, sonicated (3 × 30 seconds) and centrifuged for 10 minutes at 14,000 g. Protein concentration was measured with the Pierce™ BCA Protein Assay Kit (cat# 232225, Thermo Fisher Scientific), and volume corresponding to 25 *μ*g protein was further reduced in 0.1 M DTT and processed into peptides using filter-aided sample preparation [18]. Prior to usage, all filters were checked with a simple centrifugation step [19] in order to exclude nonretaining protein membrane filters. The peptide solutions were desalted with Oasis HLB 96-well *μ*Elution plate (cat# 186001828BA, Waters) using 0.1% formic acid (FA) and 80% acetonitrile (ACN)/0.1% FA as binding and elution buffers, respectively. Eluted peptides were vacuum dried and dissolved in 2% ACN, 1%FA prior to LC-MS analysis.

#### 2.10.2. LC-MS

For proteomic analysis, approximately 1 *μ*g peptides per sample, dissolved in 2% ACN, 0.1% FA, were injected into an Ultimate 3000 RSLC System (Thermo Scientific, Sunnyvale, California, USA) connected online to a Q-Exactive HF mass spectrometer (Thermo Scientific, Bremen, Germany) equipped with EASY-Spray nano-electrospray ion source (Thermo Scientific). The sample was loaded and desalted on a precolumn (Acclaim PepMap 100, 2 cm × 75 *μ*m ID nanoViper column, packed with 3 *μ*m C18 beads) at a flow rate of 5 *μ*L/min for 5 min with 0.1% trifluoroacetic acid (TFA). Peptides were separated during a biphasic ACN gradient from two nanoflow UPLC pumps (flow rate of 200 nL/min) on a 50 cm analytical column (PepMap RSLC, 50 cm × 75 *μ*m ID EASY-Spray column, packed with 2 *μ*m C18 beads). Solvents A and B were 0.1% FA (vol/vol) in water and 100% ACN, respectively. The gradient composition was 5% B during trapping (5 min), followed by 5-8% B over 0.5 min, 8–24% B for the next 109.5 min, 24–35% B over 25 min, and 35–90% B over 15 minutes. Elution of very hydrophobic peptides and conditioning of the column were performed during 15 minutes isocratic elution with 90% B and 20 minutes isocratic conditioning with 5% B.

The eluting peptides from the LC-column were ionized in the electrospray and analyzed by the Q-Exactive HF. The mass spectrometer was operated in the DDA mode (data-dependent acquisition) to automatically switch between full-scan MS and MS/MS acquisition. Instrument control was through Q Exactive HF Tune 2.4 and Xcalibur 3.0. MS spectra were acquired in the scan range 375-1500 *m*/*z* with resolution *R* = 120,000 at *m*/*z* 200, automatic gain control (AGC) target of 3*e*6, and a maximum injection time (IT) of 100 ms. The 15 most intense eluting peptides above intensity threshold 50 000 counts and charge states 2 to 5 were sequentially isolated to a target value (AGC) of 1*e*5 and a maximum IT of 100 ms in the C-trap, and isolation width maintained at 1.6 *m*/*z* (offset of 0.3 *m*/*z*), before fragmentation in the HCD (Higher-Energy Collision Dissociation) cell. Fragmentation was performed with a normalized collision energy (NCE) of 28%, and fragments were detected in the Orbitrap at a resolution of 15 000 at *m*/*z* 200, with first mass fixed at *m*/*z* 100. One MS/MS spectrum of a precursor mass was allowed before dynamic exclusion for 20 s with “exclude isotopes” on. Lock-mass internal calibration (*m*/*z* 445.12003) was used. Furthermore, for spray and ion-source parameters, the ion spray voltage was at 1800 V, no sheath and auxiliary gas flow, and the capillary temperature was at 260°C.

#### 2.10.3. MaxQuant Analysis

The raw MS files were searched in MaxQuant (v1.5.8.3) [20] using the default parameters with the following exceptions: label-free quantification was set to LFQ, minimum peptide length was set to 6 amino acids, and the match-between-runs option was enabled. The cellular protein levels were relatively quantified using the MaxLFQ algorithm [21], and these intracellular levels are presented as the relative LFQ intensity defined as the normalized relative protein abundance compared across the MS runs.

### 2.11. Postprocessing of the Proteomic Data

MaxQuant normalized expression data (LFQ intensities) were log_2_ transformed. Reverse hits and contaminates were removed. All samples had missing values which is common for low abundant proteins; however, to avoid too many missing values we only considered proteins with expression values in at least 14/20 samples. For every protein, the fold changes (FC) between stable and unstable were evaluated by subtracting the median of the respective logarithm transformed intensities. Next, we used *Z*-statistics [22] to evaluate the significance of the FC (referred to as FC significance), and FC *p* values < 0.05 were considered significant. The Perseus software (v.1.6.2.3) was used to analyze and visualize the data [23]. Principal component analysis (PCA) was performed in Perseus and used to compare the reprogrammed lines using the protein abundance. Missing values are incompatible with this approach; therefore, we filtered the protein abundance matrix to only contain valid values. Unsupervised hierarchical clustering was performed in Perseus on *z*-normalized abundance values. The parameters for clustering were average linkage and Euclidean correlation as distance measurement, prepossessed with *k*-means.

### 2.12. Pathway Analysis of the Proteomic Data

Pathway analysis of the proteomic data was performed in Ingenuity Pathway Analysis (IPA) software. Proteins being more abundant in the unstable colony morphology group (*n* = 338) and proteins being more abundant in the stable colony morphology (*n* = 276) were analyzed in IPA to find networks and upstream regulators in the two groups. We used log-transformed *z*-normalized abundance values. “Select identifier type” was set to “Gene symbol-human” and “Measurement annotation for observation” was set to “expr log ratio.” We performed a core analysis and used the default setting except the following: 70 molecules per network and 25 networks and all tissue and cell lines.

### 2.13. Receiver Operating Characteristic (ROC) Curves

ROC curves were generated in GraphPad Prism by using the default settings including a confidence interval of 95% calculated by using the Wilson/Brown method.

### 2.14. EMT Reversal Experiment Using Ligands

Cells were seeded in wells in a 24-well plate, each well containing a 9 mm cover slip subsequently coated with Matrigel. For the first replicate, cells were harvested prior to the experiment with the Gentle Cell Dissociation Reagent and 50 000 cells were seeded in each well. For the second replicate, cells were harvested with TrypLE™ Select and 100,000 filtered cells were seeded in each well. In both experiments, the cells were treated daily with ALX-270-445 (10, 25, and 50 *μ*M) or A83-01 (0.2, 1, and 10 *μ*M) or SMURF1-i (2, 10, and 25 *μ*M) and the cover slips were collected and fixed after 7 days. The immunofluorescence protocol was performed following directions provided by the supplier, and the following antibodies were used: mouse anti-human E-cadherin (1/250, cat# ab76055, Abcam) and rabbit anti-human vimentin (1/100, cat# 5741, CST). The following secondary antibodies were used: donkey anti-rabbit A647 and donkey anti-mouse A594. The secondary antibodies were all from Molecular Probes (dilution 1/500). The nuclei were stained with DAPI. The samples were mounted in ProLong Diamond Antifade Mountant Media. The expression of E-cadherin and vimentin were analyzed by using the Andor Dragonfly 505 (Andor Technologies, Inc.) confocal microscope with a 20x dry objective (CFI Plan Apochromat Lambda 20x). The immunofluorescence was quantified using the Imaris software (v9.2.1). No specific feature of the original data was obscured, eliminated, or misrepresented.

### 2.15. Statistical Analysis

Statistical analysis was performed in Excel (v14.7.7) and GraphPad Prism (v7.0.0). A two-sided *t*-test was used. *p* values < 0.05 were considered significant.

## 3. Results

### 3.1. Generation and Morphological Classification of Reprogrammed Cell Lines

**Figure 1:**
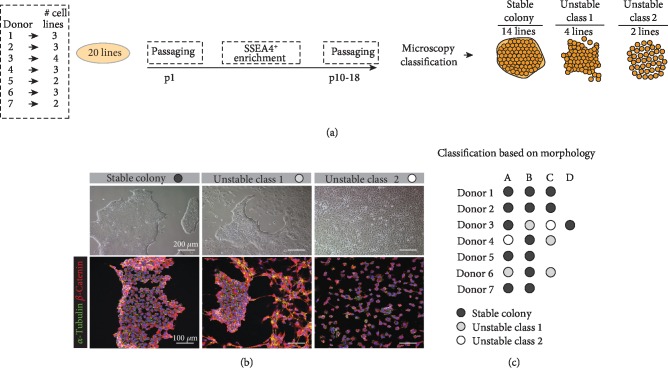
Generation of 20 reprogrammed cell lines and their classification based on morphology. (a) A total of 20 lines were SSEA4^+^ enriched and passaged >P10 and subsequently microscopically classified into one of the tree morphology groups; stable colony (14 cell lines), unstable class 1 (4 cell lines), and unstable class 2 (2 cell lines). (b) In the upper panel, phase-contrast images illustrate a representative morphology for the different groups and in the lower panel immunofluorescence images showing the organization of the structural proteins *α*-tubulin and *β*-catenin. (c) An overview of the 20 reprogrammed lines, organized by donors. The generated lines from each donor are named with the letters A–D. The classification is indicated by colour where black = stable colony, grey = unstable class 1, and white = unstable class 2.

We used fibroblast cells isolated from seven donors' skin biopsies to generate 20 reprogrammed cell lines (Figure 1(a), supplementary [Supplementary-material supplementary-material-1]). Donor 1-4 fibroblasts were reprogrammed using episomal plasmids [17] while donor 5-7 fibroblasts were reprogrammed using the Sendai virus. Each donor generated 2-4 reprogrammed cell lines each. After reprogramming, all lines presented a typical pluripotent colony morphology. However, after subsequent enrichment of SSEA4^+^ positive cells and further culturing, four of the lines had changed their colony morphology to a state with disintegrating colonies and two of the lines had changed colony morphology to a monolayer state with completely dispersed cells, referenced to in the remaining part of this paper as *class 1* and *class 2 unstable lines*, respectively (Figures 1(a) and 1(b)). The cell lines were maintained in the same culturing conditions and split when they reached 80% confluence. At around passage 13, the lines were qualitatively classified into the three colony morphology groups (stable and unstable class 1 and 2) by the use of a phase-contrast microscope (Figure 1(c)). Reprogrammed lines generated from donors 1, 2, 5, and 7 were all classified as lines showing stable colony morphology, whereas reprogrammed lines generated from donors 3, 4, and 6 included some cell lines showing unstable colony morphology and some cell lines showing stable colony morphology.

### 3.2. The Colony Morphology of Reprogrammed Cells Predicts Differences in Spontaneous and Directed Differentiation Capacity

**Figure 2:**
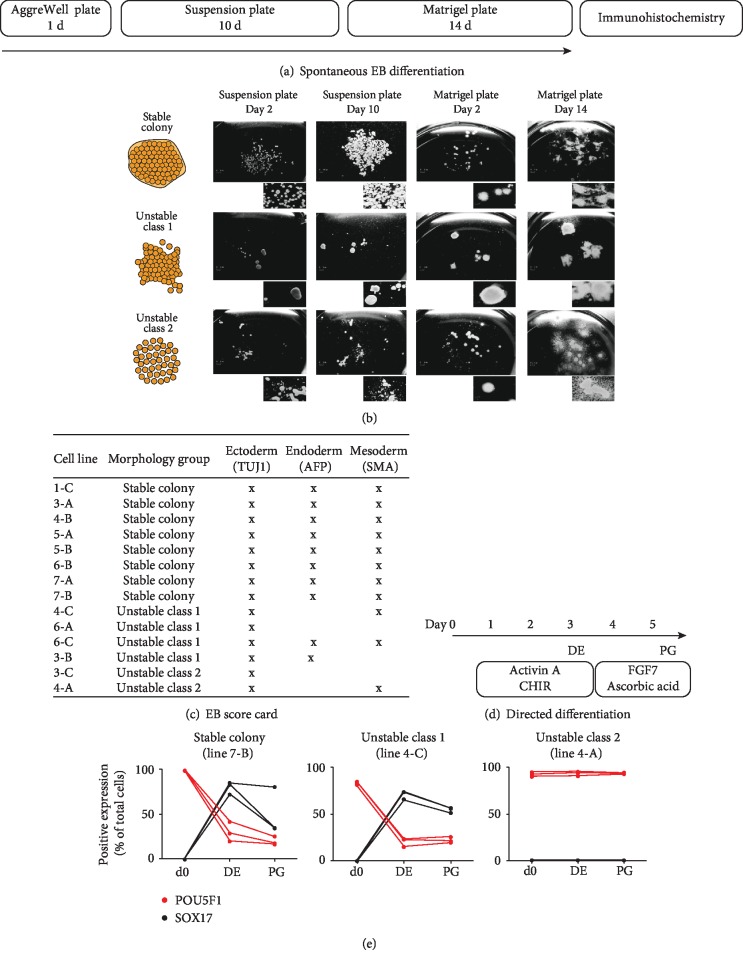
Colony morphology is associated with the capacity to form EB and directed differentiation. (a) An overview of the EB formation protocol. (b) Phase-contrast images of the spontaneously differentiated EB at the following time points; Day 2 and Day 10 in suspension plates and Day 2 and Day 14 on Matrigel-coated plates. (c) A score card of the ability to form the different embryonic germ layers for 14 selected cell lines. The analysis was performed at the end of the protocol (Day 29) and analyzed by immunohistochemistry for the markers TUJ1 (ectoderm), AFP (endoderm), and SMA (mesoderm). (d) An overview of the directed differentiation protocol towards definite endoderm (DE) and primitive gut tube (PG). (e) The directed differentiation capacity as measured with flow cytometry analysis for the pluripotency marker POU5F1 and the DE marker SOX17 in line 7-B (stable colony), line 4-C (unstable class 1), and line 4-A (unstable class 2). There were no SOX17+ cells in line 4-A at any of the time points.

We then assessed how the variation in colony morphology of the reprogrammed cell lines affected the spontaneous and directed differentiation capacity. First, we assessed spontaneous differentiation by testing the capacity to form embryonic bodies (EB) in 14 selected lines. We used AggreWell plates for EB formation, followed by 10 days of culture in suspension plates and 14 days on Matrigel-coated plates, and subsequently analyzed the EB by immunohistochemistry using markers for ectoderm (TUJ1), endoderm (AFP), and mesoderm (SMA) (Figure 2(a)). Already at day 2 in suspension, a difference was noticeable, where EB from stable colonies stayed as individual spheres, whereas EB from unstable class 1 and class 2 formed aggregates (Figure 2(b)). After completing the 29 days of the EB formation protocol, we found, as expected, that all the reprogrammed lines with stable colony morphology were able to form all three germ layers (Figure 2(c)). In contrast, reprogrammed lines with unstable class 1 morphology and unstable class 2 morphology were only able to reliably form ectoderm. Two of the lines (lines 6-A and 3-C) could only form ectoderm. Two of the lines (lines 4-A and 4-C) could form ectoderm and mesoderm, while one of the lines (line 3-B) could form ectoderm and endoderm. Only one of the unstable class 1 lines (line 6-C) was able to form all three germ layers. An overview showing immunohistochemistry images for the lines can be found in supplementary [Supplementary-material supplementary-material-1].

Next, we investigated the directed differentiation capacity using ligands that directed the reprogrammed lines (d0 stage) towards definite endoderm (DE stage) and furthermore to primitive gut tube (PG stage) (Figure 2(d)). One representative line from each colony morphology group was analyzed by flow cytometry (3 replicates per line) at the starting point, at the DE stage, and at the PG stage. In order to analyze the capacity to exit the pluripotent state and enter and exit the DE stage, we analyzed the cells by flow cytometry at all three time points (d0, DE, and PG) for cells expressing POU5F1 (pluripotency marker also known as OCT4) and SOX17 (essential transcription factor in the formation and maintenance of DE [24]) (Figure 2(e)). We found that the reprogrammed line with stable colony morphology had 99% ± 0.2 SD POU5F1+ cells at d0, dropping to 30% ± 11 SD at DE and 20% ± 4.8 SD at PG (Figure 2(e)). As anticipated, SOX17 was undetectable at d0 and increased to 80% ± 6.9 SD at the DE stage before dropping to 50% ± 26 SD at the PG stage. For the reprogrammed line with unstable class 1 colony morphology, we observed a similar pattern, albeit with only 83% ± 1.6 SD POU5F1+ cells at d0 and 71% ± 4.4 SD SOX17+ cells at the DE stage. Finally, for the unstable class 2 colony morphology line, we found 93% ± 2.4 SD POU5F1+ cells at d0, and the level stayed high at the DE stage (94% ± 2.4 SD) and at the PG stage (95% ± 0.7 SD) with no observable SOX17+ cells at any time point. Taken together, the unstable colony morphology was associated with impaired directed differentiation capacity and reduced capacity to form the three germ layers.

### 3.3. The Variable Colony Morphology Groups Show Distinctly Different Proteomic Signatures

**Figure 3:**
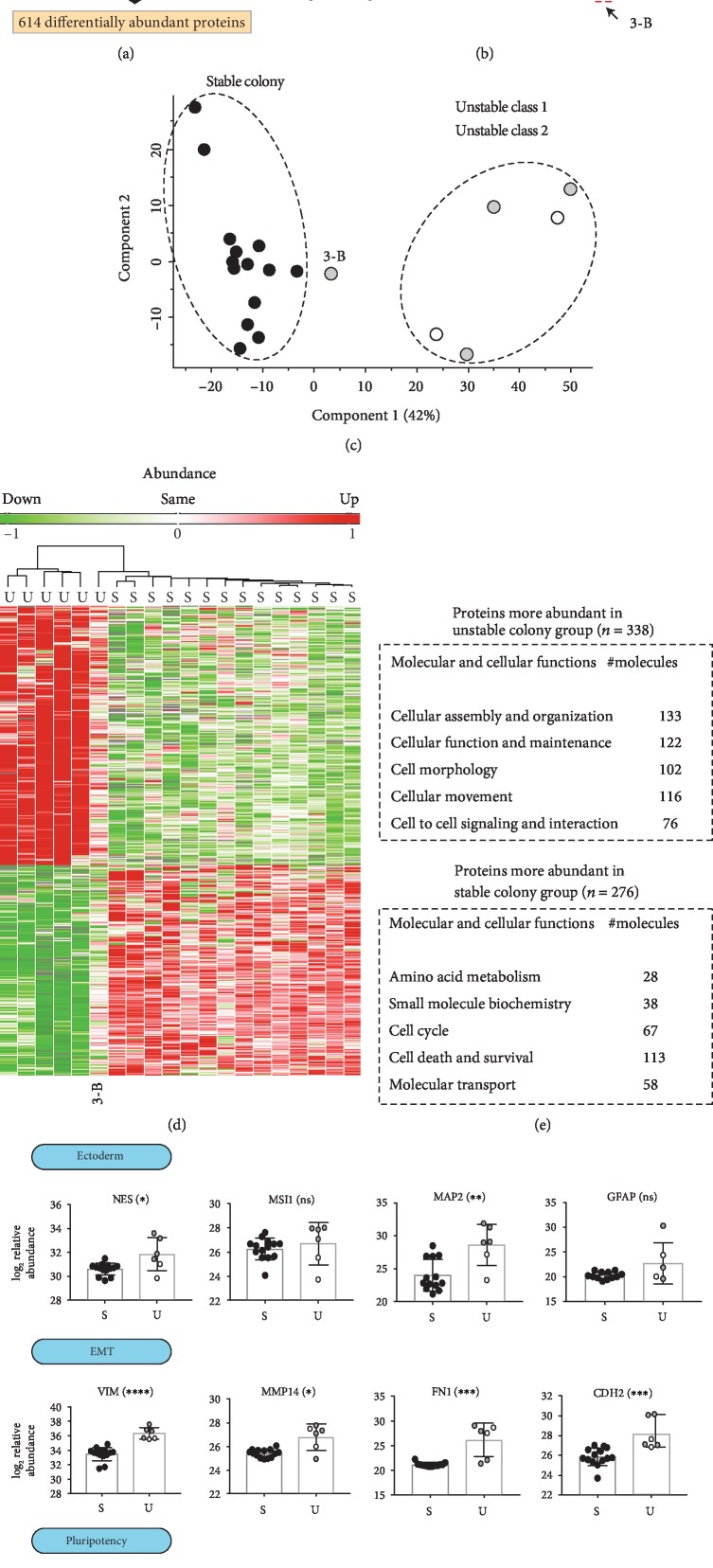
Global proteomics of the 20 reprogrammed cell lines reveals different proteomic signatures for the different colony morphology groups. (a) Workflow for the proteomic experiment analyzing the global proteome of 20 reprogrammed cell lines microscopically classified into stable colony morphology (14 lines), unstable class 1 colony morphology (4 lines), and unstable class 2 colony morphology (2 lines). The samples are cell lysates from the corresponding cell line. The samples were quantified using label-free proteomics, which yielded ~5000 proteins in each sample. Raw values were log_2_ transformed, and fold changes (FC) between the combined unstable colony morphology group (6 lines) and stable colony morphology group (14 lines) were calculated. Proteins with a *p* value < 0.05 and a FC *p* value < 0.05 (*n* = 614) were regarded as being differentially abundant between the groups. (b) Unsupervised clustering analyses of the 20 reprogrammed lines based on proteins expressed in at least 14/20 samples (*n* = 5043) showing that the clusters tend to associate more with morphology and less with donor sex and reprogramming methods. S = stable colony morphology; U = unstable colony morphology. (c) Principal component analysis (PCA) of proteins expressed in all samples (*n* = 3833) shows a clear separation between stable colony morphology (black circles) and unstable colony morphology (grey and white circles). (d) Unsupervised clustering analyses of the 614 differentially abundant proteins. The clustering analysis revealed two major clusters separated by colony morphology. (e) Tables describing molecular and cellular functions of the protein being more abundant in the unstable colony morphology group (*n* = 338) and the stable colony morphology group (*n* = 276). (f) A selection of differentially abundant proteins in the morphology groups. EMT markers (VIM, MMP14, FN1, and CDH2) and ectoderm markers (NES, MAP2) were more abundant in the unstable colony morphology group (U), whereas pluripotency markers (PODXL, DPPA4, DNMT3B, and CDH1) were more abundant in the stable colony morphology group (S). Statistical analysis was performed with Mann-Whitney *t*-test. Significant differences are shown as ^∗^*p* < 0.05, ^∗∗^*p* < 0.01, ^∗∗∗^*p* < 0.001, ^∗∗∗∗^*p* < 0.0001, and ns *p* > 0.05. n/a = not a number. (g) Top differentially abundant proteins ranked by fold change (stable compared to unstable). Only proteins with a *p* value < 0.05 were included in the ranking. The pluripotency markers DNMT3B, DPPA4, and SALL4, the EMT marker FN1, and the ectoderm marker MAP2 are highlighted in green (up in the stable colony morphology signature) and red (up in the unstable colony morphology signature).

Global label-free proteomics of the 20 reprogrammed lines yielded 6173 quantified proteins, with an average of ~5000 quantified proteins in each sample (Figure 3(a)). Proteins expressed in at least 14/20 samples (*n* = 5043) were analyzed by unsupervised clustering (Figure 3(b)). We found that reprogrammed lines clustered together based on colony morphology appearance and not by reprogramming method or sex of the donor. However, it should be noted that one of the unstable lines (line 3-B) clustered within the stable colony morphology group, although the line was classified as an unstable class 1 line. Next, we looked at proteins expressed in all samples (*n* = 3833) and performed a PCA analysis (Figure 3(c)). Again, we found that reprogrammed lines clustered together based on colony morphology, this time with a clear separation between samples from stable and unstable colony morphology lines. However, we noted that unstable class 1 colony morphology and unstable class 2 colony morphology samples did not separate from each other. These groups were thus merged into one common group in the remaining global proteome comparison.

Next, we looked at differentially abundant proteins (*n* = 614) comparing the stable group (14 cell lines) to the unstable group (6 cell lines) (Figure 3(d)). Significant differentially abundant proteins had by definition a *p* value < 0.05 and a fold change *p* value < 0.05. We identified 338 proteins being more abundant in the unstable colony morphology group (supplement [Supplementary-material supplementary-material-1]), and we identified the top molecular and cellular functions associated with these proteins, as listed in Figure 3(e). Furthermore, in the unstable group, we identified proteins well known as markers for mesenchymal cells, including N-cadherin (CDH2), fibronectin (FN1), vimentin (VIM), and matrix metallopeptidase 14 (MMP14) (Figure 3(f)). We also investigated whether we could detect protein markers for any of the three germ layers in the unstable colony morphology group. Endoderm markers (SOX17, GATA4, GATA6, and EOMES) and mesoderm markers (TBXT, FOXF1) were not identified in our data set. Ectoderm markers including nestin (NES), RNA-binding protein Musashi homolog 1 (MSI1), microtubule-associated protein 2 (MAP2), and glial fibrillary acidic protein (GFAP) were identified, among which only NES and MAP2 had a significantly higher abundance in the unstable colony group (Figure 3(f)).

Similarly, we identified 276 proteins being more abundant in the stable colony morphology group (supplement [Supplementary-material supplementary-material-1]) and we identified the top molecular and cellular functions associated with these proteins (Figure 3(e)). Furthermore, we detected protein markers for pluripotency including podocalyxin-like protein 1 (PODXL), developmental pluripotency-associated 4 (DPPA4), and DNA (cytosine-5-)-methyltransferase 3 beta (DNMT3B) (Figure 3(f)). We also noted a significant higher abundance of E-cadherin (CDH1) in the stable colony morphology group. Together with the significant higher abundance of N-cadherin (CDH2) in the unstable colony morphology group, our observations are in line with a cadherin switch (increase of CDH2 and a decrease of CDH1) previously described in EMT events [25].  Figure 3(g) shows the ranked fold changes for the individual proteins providing the signature for both morphology groups.

### 3.4. Common Markers for Pluripotency Did Not Vary Significantly between Reprogrammed Lines Showing Stable and Unstable Colony Morphologies

**Figure 4:**
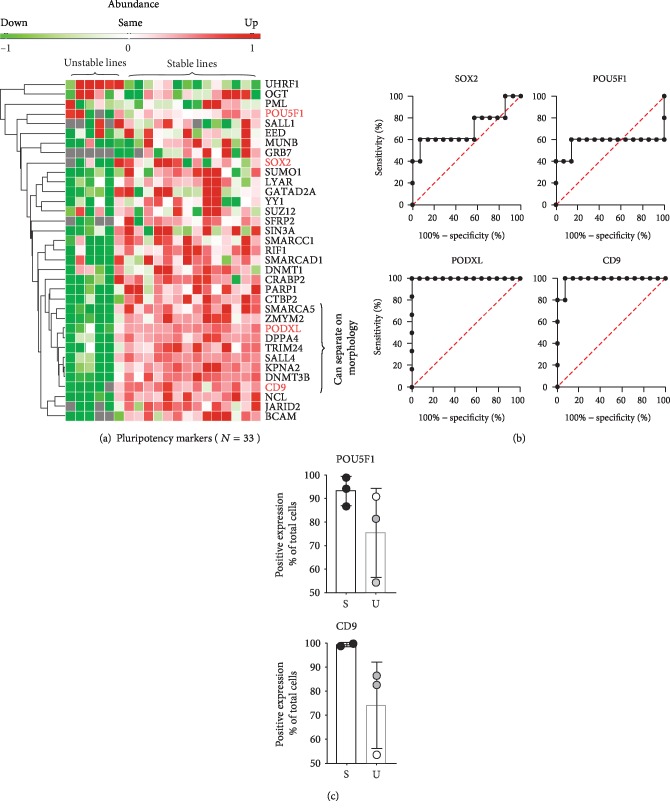
Variations in pluripotency markers and the ability to separate the stable colony morphology group from the unstable colony morphology group. (a) A heat map of 33 commonly used pluripotency markers [26, 27] identified in our proteomic data set. The cluster analysis was only applied on rows, not columns. The heat map revealed a group of 10 markers that were able to separate the two morphology groups. (b) ROC curves for POU5F1, SOX2, PODXL, and CD9 when comparing the stable colony morphology group with the unstable colony morphology group. (c) Flow cytometry analysis of the markers POU5F1 and CD9 in selected stable (S) and unstable (U) lines.

Surprisingly, the abundance of the common pluripotency markers sex-determining region Y (SOX2) and octamer-binding transcription factor 4 (POU5F1) was not significantly more abundant in the stable colony morphology group compared to the unstable colony morphology group (Figure 4(a)), with *p* values of 0.22 and 0.69, respectively (not shown). Furthermore, we asked if other common pluripotency markers had a higher abundance in the stable colony morphology group compared to the unstable colony morphology group, which could serve as a potential marker for stable colony morphology. Based on well-known pluripotency markers previously published [26, 27], we identified 33 out of a total of 49 markers in our data set and further assessed the abundance of these 33 markers in stable and unstable morphology groups (Figure 4(a)). Indeed, we identified that a subgroup of around ten proteins including CD9 antigen (CD9) and PODXL was more abundant in the stable colony morphology group, with *p* values of 0.0003 and 0.0002, respectively. Next, the separation efficiency for POU5F1, SOX2, PODXL, and CD9 to distinguish between the two groups (stable and unstable) was evaluated by making receiver operating characteristic (ROC) curves (Figure 4(b)). Indeed, SOX2 and POU5F1 showed low ability to distinguish between the two groups, with area under the curve (AUC) values of, respectively, 0.62 and 0.5. In contrast, PODXL and CD9 could distinguish between the groups, with AUC values of respectively 1 and 0.99. In order to validate the proteomic data, we measured the levels of CD9 and POU5F1 by flow cytometry in selected stable and unstable colony morphology lines (Figure 4(c)). There was a tendency, although not significant, towards lower POUF5 and CD9 levels in the unstable colony morphology lines.

### 3.5. Pathway Analysis Suggests TGFB-Induced EMT Events in Reprogrammed Lines with Unstable Colony Morphology

**Figure 5:**
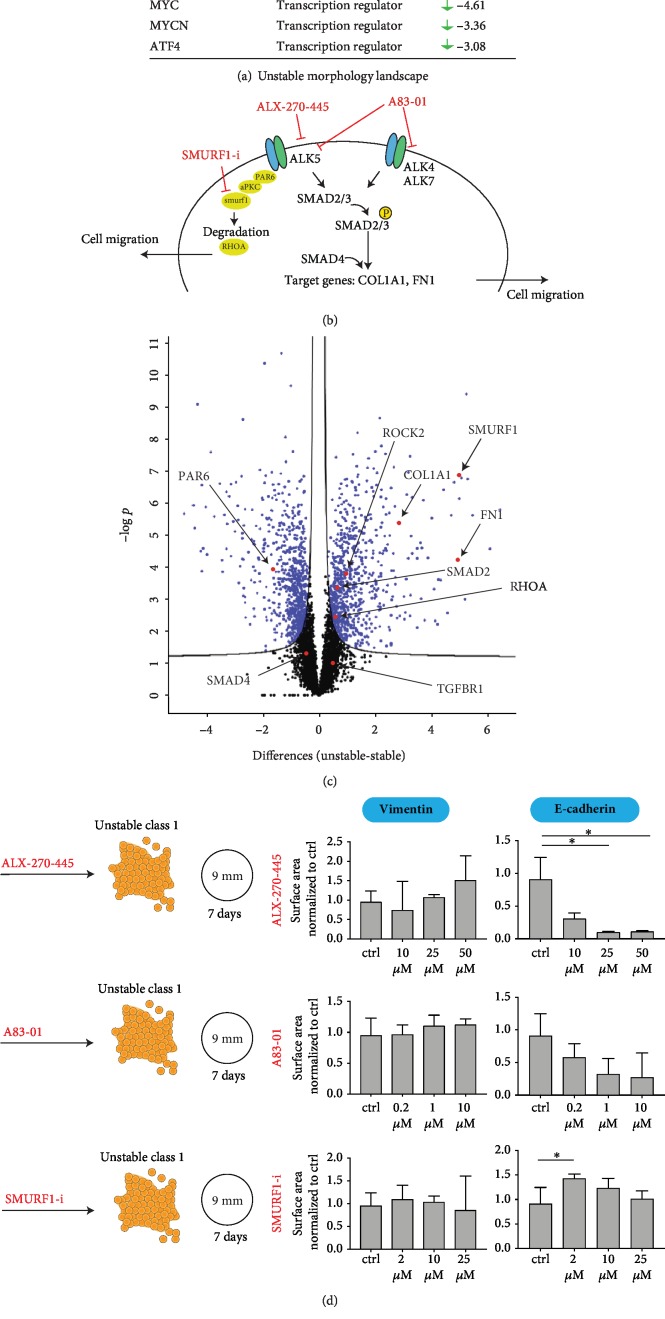
Pathway analysis of the protein landscape between the unstable colony morphology group and the stable colony morphology group. (a) IPA-generated tables of the top predicted upstream regulators in the unstable colony morphology group. (b) A model (modified from [29, 30]) illustrating mechanistic factors involved in the EMT effect caused by TGFB signalling and the small molecules we applied in the experiment that lead to the results illustrated in (d). (c) A volcano plot showing proteins being more abundant in one of the groups (blue dots), where a selection of proteins related to EMT and TGFB signalling is highlighted in red. (d) An illustration of the experiment where cells from unstable class 1 (line 4-C) were seeded on 9 mm cover slips and treated for 7 days with the ligands ALX-270-445, A83-01, or Smurf1-i. Graphs showing quantitative immunofluorescence (surface area) of vimentin (EMT marker) and E-cadherin (colony marker) in line 4-C after treatment with the ligands ALX-270-445, A83-01, and SMURF1-i at varying concentrations. The analysis was done by using the Andor Dragonfly 505 microscope and subsequently quantified by using the Imaris software, where raw surface area measurements of vimentin and E-cadherin were normalized on nucleus count for each cover slip. Expression is given as ratio to the median of the control samples (*n* = 8). Statistical analysis was performed with Mann-Whitney test. Significant differences are shown as ^∗^*p* < 0.05.

To identify upstream regulators in the unstable morphology group, we performed pathway analysis using the IPA (Ingenuity Pathway Analysis) software tool. In this analysis, we used the differentially abundant proteins (*n* = 614, displayed in Figure 3(d)) and asked which upstream regulator proteins could explain the emergence of this protein signature *in silico*. The top scoring upstream regulators in the unstable morphology group were TCF7L2 followed by CTGF and TGFB1 (Figure 5(a) and supplementary [Supplementary-material supplementary-material-1]). Since TGFB is the major signalling pathway for inducing EMT [28], we sought to focus on TGFB as an upstream regulator in the unstable morphology group. The canonical TGFB signalling is activated by ligands that act on TGFB receptors with subunits ALK4, ALK5, and ALK7 [29] (Figure 5(b)). SMAD2/3 is subsequently phosphorylated and together with SMAD4 enters the nucleus to activate transcription factors and regulate target genes. An alternative cascade occurs though SMURF1-regulated RHOA degradation that mediates EMT [30] (Figure 5(b)). In this case, the activated receptor phosphorylates PAR6, thereby stimulating the recruitment of SMURF1 and leading to tight junction dissolution, which is a characteristic of EMT. In our proteomic data set, several of the molecules involved in both the canonical TGFB route to EMT and the alternative SMURF-regulated route to EMT were identified and displayed in the volcano plot (Figure 5(c)). For the canonical TGFB signalling pathway, SMAD2 was found to be more abundant in the unstable colony morphology group together with downstream target molecules such as COL1A1 and FN1. For the SMURF1-regulated route, SMURF1 itself was found to be one of the most abundant proteins in the unstable colony morphology group compared to the stable colony morphology group (Figure 5(c)). Furthermore, we have conflicting data as PAR6 was found to be less abundant and RHOA was found to be more abundant in the unstable colony morphology group. From these different protein abundances, we hypothesize enhanced activity in the canonical TGFB route and a modified activity in the alternative SMURF1-regulated route in the unstable colony morphology group.

Trying to validate these findings experimentally, we selected a reprogrammed line with unstable class 1 colony morphology (line 4-C) previously identified with high expression of EMT markers (Figure 3(f)) and exposed it to TGFB inhibitors (ALX-270-445 and A83-01) and a SMURF1 inhibitor (Smurf1-i) to see whether these ligands could reverse the EMT event which would be indicated by an increase in the colony marker E-cadherin (CDH1) and a decrease in the EMT marker vimentin (VIM) [31]. We treated the line for seven days with each drug at three different concentrations and quantified the level of vimentin and E-cadherin by immunocytochemistry (Figure 5(d)). Although there were observable alterations in the quantified levels, none of the ligands led to significantly decreased levels of vimentin or significantly increased levels of E-cadherin, and we did not observe a reversal of colony morphology (towards stable colony morphology, not shown).

## 4. Discussion

In this study, we used label-free quantitative proteomics to compare reprogrammed cell lines displaying stable colony morphology to lines with unstable colony morphology. Colony morphology is typically considered an important criterion for undifferentiated pluripotent cells and is a valuable assessment in the daily routine in stem cell laboratories. However, this assessment suffers from manual and subjective microscopic inspection and is therefore questionable in an automated pipeline for benchmarking of cells [32].

By providing a first proteomic characterization of the molecular signatures of reprogrammed cells displaying different colony morphologies, our results demonstrate proteome signature patterns robustly capturing the colony morphology and provide an insight into the molecular mechanisms involved in spontaneous differentiation. The protein signatures presented here could represent a base for next-generation benchmarking of pluripotent cells, correlating protein profiles with colony morphology, which is considered a critical indicator of true pluripotent cells.

In the unstable colony morphology group, we found higher abundance of mesenchymal markers including vimentin (VIM), N-cadherin (CDH2), and fibronectin (FN1). This is in line with previous reports [25, 31, 33–36]. In fact, the presence of mesenchymal-like cells in colonies that undergo spontaneous differentiating was first time reported in 2001 [37]. Furthermore, epithelial to mesenchymal transition (EMT) was subsequently identified and associated with spontaneous differentiation [33, 34]. However, EMT markers in differentiating PSCs have mainly been shown by immunohistochemistry and Q-PCR [25, 33, 35] and also using RNA-seq [38] and DNA microarray [31, 36]. In our study, we show for the first time that mass spectrometry-based proteomics can identify similar EMT profile and also capture the broader molecular picture of this event.

It is known that EMT can be induced via several pathways [28]; however, the mechanisms triggering EMT in stem cells are not fully understood. Already in 2005, D'amour et al. discovered an Activin A-induced EMT in the differentiation to DE; however, it was not clear which signalling pathway was involved [39]. Later in 2017, Li et al. showed that Activin A-induced formation of DE includes an EMT event triggered by TGFB signalling [38]. This is in line with our global proteomic assay where the pathway analysis is suggestive for a TGFB-induced event in the unstable colony morphology group. We identified TGFB pathway molecules to be more abundant (SMAD2, SMURF1, ROCK2, and RHOA) as well as downstream target genes (COL1A1, VIM, and FN1). In our attempt to reverse EMT, we tried to inhibit the TGFB receptors by using the ligands ALX-270-445 (inhibits ALK 5 subunit) and A83-01 (inhibits ALK 4, 5, and 7 subunits). We also attempted to inhibit SMURF1 as this TGFB-related protein had a high abundance in the unstable colony morphology group in our data. By using the selected ligands, we observed an alteration in vimentin and E-cadherin expression; however, a reversal of EMT indicated by an increased level of E-cadherin and a decreased level of vimentin was not observed. As EMT can be induced via several pathways and crosstalk can occur [40], the role of the molecules we are targeting can possibly be replaced by other signals. Feng et al. showed for example in 2012 that an activation of PKC is associated with EMT in stem cells, and Kinehara et al. showed in 2014 that by using a PKC-inhibitor the EMT was reversed [31].

The underlying reason for the dynamic change of the PSC colony morphology is not fully understood. Epigenetic memory and an incomplete reprogramming could be one explanation [41]. Furthermore, the feeder-free system has been reported to cause unwanted spontaneous differentiation [37], especially when using Matrigel [42]. Both these findings could explain the variation in our sample set. Cell competition was recently found to be a mechanism during reprogramming where elite cells overtake the cell population [43]. Cell competition could also explain changing in colony morphology at a later passage where differentiated cells out-compete nondifferentiated cells. Also, variation in hiPSC lines has been shown to be donor dependent [5, 44, 45]; our studies, however, showed that variations related to colony morphology are not donor dependent, as three of the donors (donors 3, 4, and 6) had lines classified to more than one morphology group.

The differentiation potential associated with colony morphology is an important aspect as this is a crucial function of PSCs. In our study, we found that reprogrammed lines with unstable colony morphology could form ectoderm; however, the extent of endoderm and mesoderm formation was varying. There have been some studies correlating different classes of PSCs to differentiation capacity; however, most of them have showed a successful formation of the three germ layers in all classes or only tested a selection of qualified lines [11, 12]. Only a few studies have showed varying differentiation potential; for example, Chen et al. published in 2009 a study where hESCs were classified in three morphology groups and found that *in vivo* differentiation capacity, measured by teratoma formation in mice, differed for the classes [6]. However, the hESC classes were based on expression markers, not colony morphology. Also, Wakao et al. published in 2012 a study where only one out of seven iPSC classes could successfully form EB [13]. However, the iPSCs were classified based on cell characteristics and not the overall colony morphology. To our knowledge, our study is unique in classifying the reprogrammed lines (>P10) based on overall colony morphology and correlation to EB formation capacity.

For the PSCs and regenerative medicine field, the safety aspect is unavoidable. Changes and variations in PSC are partly unpredictable, and it is important to evaluate the cells routinely. As typical and common markers for pluripotency have been questioned [13], more comprehensive automated assays to benchmark cells are needed to ensure a sufficient quality control. Our proteomic data show distinct proteomic profiles for the colony morphology groups; hence, the proteomic analysis reflects the colony morphology and the PSC status. In this study, we demonstrate the validity of using proteomics to monitor reprogrammed lines and suggest that it should be part of an automated assay to benchmark cells.

## 5. Conclusion

In this study, we classified 20 reprogrammed cell lines based on colony morphology and subsequently tested their differentiation capacity and analyzed their proteomic profiles using mass spectrometry. We found that different defined patterns of colony morphology were associated with distinct proteomic profiles and different outcomes in differentiation capacity. Finally, we provided insight into possible molecular mechanisms involved in the formation of stable and unstable colony morphologies during reprogramming.

## Data Availability

The raw MS data files are available via ProteomeXchange with identifier PXD013481.

## Supplementary Materials

Supplementary 1 Supplementary table 1: information about the hiPSC lines included in the study. Supplementary table 2: a list of proteins being more abundant in stable colony morphology group. Supplementary table 3: a list of proteins being more abundant in the unstable colony morphology group. Supplementary table 4: activated upstream regulators in the unstable colony morphology group.

Supplementary 2 Supplementary figure 1: immunohistochemistry images of all embryonic bodies.
