# Supplementary material for: Reprogrammed Cells Display Distinct Proteomic Signatures Associated with Colony Morphology Variability
Source: Stem Cells Int. 2019 Nov 18;2019:8036035. doi: 10.1155/2019/8036035 (PMC6885794; doi:10.1155/2019/8036035)
Supplement: Supplementary 1 — Supplementary table 1: information about the hiPSC lines included in the study. Supplementary table 2: a list of proteins being more abundant in stable colony morphology group. Supplementary table 3: a list of proteins being more abundant in the unstable colony morphology group. Supplementary table 4: activated upstream regulators in the unstable colony morphology group. [file 8036035.f1.zip › Supplementary table 1.pdf]

Supplement table 1: Information about the reprogrammed lines included in the study

cell line	donor	reprograming method	donor sex	microscopic classification	passage SSEA4+	passage classified
1-A	donor 1	Episomal	f	stable	P6	p11
1-B	donor 1	Episomal	f	stable	p5	p10
1-C	donor 1	Episomal	f	stable	p6	p11
2-A	donor 2	Episomal	m	stable	p5	p12
2-B	donor 2	Episomal	m	stable	p5	p11
2-C	donor 2	Episomal	m	stable	p3	p7
3-A	donor 3	Episomal	m	stable	p8	p12
3-B	donor 3	Episomal	m	unstable, class 1	p6	p13
3-C	donor 3	Episomal	m	unstable, class 2	p5	p14
3-D	donor 3	Episomal	m	stable	P5	p11
4-A	donor 4	Episomal	m	unstable, class 2	p7	p12
4-B	donor 4	Episomal	m	stable	p8	p17
4-C	donor 4	Episomal	m	unstable, class 1	p6	p12
5-A	donor 5	sendai virus	m	stable	p11	p18
5-B	donor 5	sendai virus	m	stable	p11	p16
6-A	donor 6	sendai virus	m	unstable, class 1	p11	p17
6-B	donor 6	sendai virus	m	stable	p11	p18
6-C	donor 6	sendai virus	m	unstable, class 1	p11	p17
7-A	donor 7	sendai virus	f	stable	p11	p17
7-B	donor 7	sendai virus	f	stable	p11	p16
