# Supplementary material for: Reprogrammed Cells Display Distinct Proteomic Signatures Associated with Colony Morphology Variability
Source: Stem Cells Int. 2019 Nov 18;2019:8036035. doi: 10.1155/2019/8036035 (PMC6885794; doi:10.1155/2019/8036035)
Supplement: Supplementary 1 — Supplementary table 1: information about the hiPSC lines included in the study. Supplementary table 2: a list of proteins being more abundant in stable colony morphology group. Supplementary table 3: a list of proteins being more abundant in the unstable colony morphology group. Supplementary table 4: activated upstream regulators in the unstable colony morphology group. [file 8036035.f1.zip › Supplementary table 2.pdf]

Supplement table 2: Proteins being more abundant (named by gene names) in stable colony morphology group.
S=stable colony morphology group,U-1= Unstable class 1 morphology group, U-2=unstable class 2 morphology group .
FC=fold change. The list is sorted by FC. All proteins have a p-value <0.05 and a FC p-value <0.05. The list includes 276 proteins.
Expression values are log2 transformed LFQ intensity values.

Gene names	1-C S	3-A S	4-B S	5-A S	5-B S	6-B S	7-A S	7-B S	3-D S	1-A S	1-B S	2-A S	2-B S	2-C S	3-B U-1	6-A U-1	6-C U-1	4-C U-1	3-C U-2	4-A U-2	Median S	Median U	p-value	FC S/U	FC p-value
RBPMS2	28.2	28.5	27.8	28.5	27.4	27.4	28.3	27.9	28.0	28.4	28.1	28.4	27.6	28.0	27.1	21.3	22.3	24.6	22.4	NaN	28.0	22.4	0.0	5.6	0
EPCAM	28.4	27.9	28.0	29.4	29.3	29.6	28.3	27.9	28.1	28.5	28.0	28.5	28.0	28.2	27.1	22.1	20.7	24.6	25.9	21.5	28.2	23.4	0.0	4.9	0
SMPDL3B	28.3	27.8	27.7	27.6	28.1	27.9	28.5	28.3	28.2	28.0	27.6	28.1	27.5	27.9	25.4	24.5	24.7	22.3	22.4	22.6	27.9	23.6	0.0	4.4	0
FBXO2	26.7	26.3	25.5	26.9	27.0	27.3	27.6	26.6	27.3	26.5	26.1	26.0	26.6	26.1	24.8	22.1	NaN	22.7	22.3	21.9	26.6	22.3	0.0	4.3	0
KRT19	30.4	30.0	29.9	30.2	29.7	29.7	30.1	30.1	30.1	30.0	29.4	29.8	30.0	30.0	28.6	24.5	23.3	26.9	27.0	24.4	30.0	25.7	0.0	4.3	0
TRIM71	30.4	30.3	29.8	29.7	30.0	30.0	30.0	30.1	30.1	30.1	30.3	29.5	30.1	30.4	29.8	22.8	24.6	28.1	27.1	23.3	30.1	25.8	0.0	4.2	0
ADD2	28.2	28.2	27.7	27.5	28.4	28.1	27.9	27.8	28.0	28.2	28.1	27.8	28.0	28.3	27.9	26.1	23.7	24.0	24.2	22.5	28.1	24.1	0.0	3.9	0.00
L1TD1	32.9	32.5	32.7	32.7	32.4	32.2	32.3	32.6	32.4	32.7	32.6	32.5	32.0	32.6	31.9	25.3	24.9	30.4	30.2	27.1	32.5	28.6	0.0	3.9	0.00
CYP2S1	29.5	29.3	29.6	30.4	29.3	29.3	29.3	29.1	29.6	29.2	29.1	29.9	29.2	29.5	27.8	24.0	21.6	26.9	27.4	22.6	29.3	25.4	0.0	3.9	0.00
ALPL	29.1	29.1	29.0	29.0	29.0	29.0	28.7	29.1	29.2	29.0	29.0	29.1	29.0	29.4	27.7	24.7	23.4	25.2	26.6	NaN	29.0	25.2	0.0	3.9	0.00
DNMT3B	32.6	32.2	32.2	32.2	32.5	32.6	33.2	32.2	32.4	32.4	31.7	32.7	31.2	32.3	31.0	27.3	27.7	29.3	29.4	25.0	32.3	28.5	0.0	3.8	0.00
SALL4	29.3	29.5	29.2	29.5	29.2	29.2	29.6	29.3	29.3	29.7	29.7	28.9	29.1	29.7	28.9	24.0	23.4	27.2	27.3	21.5	29.3	25.6	0.0	3.7	0.00
JARID2	27.3	26.1	25.3	26.4	27.0	25.7	26.6	27.0	26.7	27.1	26.7	26.9	24.9	26.8	25.5	21.2	NaN	23.1	23.0	NaN	26.7	23.0	0.0	3.7	0.00
CGN	28.8	29.0	26.5	28.2	28.9	29.0	28.4	28.6	28.5	28.3	27.7	28.0	27.5	28.5	25.4	26.6	23.9	24.4	26.5	22.6	28.5	24.9	0.0	3.6	0.00
APOE	30.2	30.1	29.8	30.1	29.9	30.3	29.8	29.6	30.0	29.7	29.5	29.9	28.9	30.0	28.6	25.6	22.6	27.2	27.1	25.7	29.9	26.4	0.0	3.5	0.00
DSG2	27.9	27.8	27.0	27.7	27.9	28.0	27.1	27.4	28.1	28.3	27.7	28.1	26.6	28.1	26.5	24.4	24.7	23.6	22.8	NaN	27.8	24.4	0.0	3.4	0.00
DSP	28.0	28.1	27.6	27.8	28.6	28.4	28.7	28.7	28.5	28.6	28.3	26.9	28.3	27.8	27.3	24.7	25.7	24.8	25.1	23.8	28.3	25.0	0.0	3.4	0.00
AASS	33.2	32.8	30.8	33.0	32.7	32.8	33.3	33.0	33.0	32.0	32.0	33.0	32.1	32.7	31.2	29.1	29.3	29.7	29.6	27.9	32.8	29.5	0.0	3.3	0.00
VRTN	28.9	28.7	28.9	29.3	28.9	29.2	28.4	28.5	28.8	28.6	28.4	28.7	28.2	28.4	28.0	NaN	24.6	25.8	25.0	NaN	28.7	25.4	0.0	3.3	0.00
BUB1B	27.0	26.5	25.3	25.7	26.5	27.5	26.7	27.5	26.7	27.2	26.9	25.4	26.2	27.5	26.4	22.7	23.0	24.1	23.4	NaN	26.7	23.4	0.0	3.3	0.00
DPPA4	30.1	30.0	29.8	29.9	30.8	30.9	29.8	29.1	30.3	30.0	30.0	29.8	29.5	30.4	29.6	25.6	21.5	28.0	28.0	22.5	30.0	26.8	0.0	3.2	0.00
AK4	29.6	30.3	30.4	29.5	30.0	29.4	29.0	29.1	29.9	30.1	30.0	30.1	29.7	30.1	29.5	26.5	25.3	27.5	27.3	22.2	30.0	26.9	0.0	3.1	0.00
CKMT1A	30.4	30.1	30.1	30.2	30.1	29.8	29.9	30.0	30.0	30.0	29.7	30.4	29.7	30.0	28.9	26.7	26.8	27.8	27.1	26.6	30.0	27.0	0.0	3.0	0.00
ERBB2	28.1	28.7	28.9	29.1	29.1	29.1	28.2	28.5	28.6	29.0	29.0	29.0	28.0	28.0	27.8	25.1	24.7	27.2	26.5	25.0	28.8	25.8	0.0	3.0	0.00
CGNL1	27.3	27.8	26.6	27.4	26.7	27.5	27.7	28.0	27.5	26.6	25.8	26.4	26.9	27.2	24.8	NaN	24.0	24.5	22.5	NaN	27.2	24.2	0.0	3.0	0.00
PCK2	30.7	30.6	30.2	30.7	30.9	31.0	29.7	31.1	30.7	30.4	30.3	30.7	30.7	30.8	29.9	27.1	28.8	27.8	27.7	24.6	30.7	27.7	0.0	2.9	0.00
CTH	27.2	27.1	27.3	27.9	27.2	27.5	27.8	28.3	27.9	27.1	27.3	27.4	28.0	27.8	26.7	23.9	24.5	24.5	25.5	23.6	27.4	24.5	0.0	2.9	0.00
CD9	27.8	27.4	28.2	27.7	28.0	26.6	28.0	27.5	27.6	28.4	28.0	27.7	28.1	28.0	27.4	NaN	24.1	25.3	25.1	21.5	27.9	25.1	0.0	2.8	0.00
PYCARD	27.5	26.7	26.8	25.4	26.4	26.4	27.2	27.6	27.1	27.5	26.6	26.7	25.9	26.8	25.8	NaN	23.0	23.1	23.9	24.6	26.8	23.9	0.0	2.8	0.00
KRT18	32.1	31.9	32.0	32.1	32.3	31.9	32.1	32.0	31.7	32.1	31.4	31.8	31.9	32.1	31.5	27.4	28.3	30.3	31.1	26.8	32.0	29.3	0.0	2.7	0.00
CETN3	26.2	25.5	23.1	25.9	25.0	24.2	26.4	23.0	24.0	25.3	25.3	26.1	25.5	25.5	24.2	21.6	22.8	22.6	24.1	22.1	25.4	22.7	0.0	2.7	0.00
IMPA2	27.6	27.9	26.3	25.8	28.0	25.2	25.9	27.7	27.2	26.0	26.2	26.6	26.8	27.6	25.8	23.8	23.8	24.2	22.9	25.1	26.7	24.0	0.0	2.7	0.00
MFGE8	29.2	29.8	29.3	29.0	29.3	29.0	29.0	29.3	29.7	29.5	29.0	29.6	29.9	28.4	28.2	26.3	24.6	27.6	26.8	23.8	29.3	26.6	0.0	2.7	0.00

SLC7A3	27.8	27.5	26.0	28.1	27.3	27.1	26.1	27.2	27.0	25.8	26.2	26.8	26.5	27.3	24.4	NaN	NaN	24.4	23.8	NaN	27.1	24.4	0.0	2.7	0.00
PDLIM1	30.6	29.4	26.2	29.7	30.4	30.6	30.5	30.2	30.1	30.1	29.4	29.1	28.1	29.9	27.8	27.6	27.0	28.3	27.2	23.5	30.0	27.4	0.0	2.6	0.00
PODXL	31.0	30.6	30.4	31.1	31.2	30.9	31.0	30.8	31.0	30.7	30.7	30.7	30.7	30.7	30.0	28.2	24.7	29.8	28.3	23.4	30.8	28.2	0.0	2.5	0.00
F11R	28.7	28.2	28.0	28.3	28.6	28.3	28.1	27.7	27.8	27.8	27.4	28.7	27.9	28.6	26.5	26.1	25.2	25.1	26.2	22.6	28.1	25.6	0.0	2.5	0.00
TXLNG	27.8	26.6	27.5	27.6	26.9	26.6	27.2	28.5	26.8	27.9	27.3	27.7	25.8	26.5	26.3	22.7	24.9	24.6	26.5	24.1	27.2	24.8	0.0	2.5	0.00
TUBA4A	27.8	27.5	27.5	27.5	27.2	28.0	27.8	27.5	27.7	27.2	26.9	26.8	27.5	26.7	26.0	NaN	25.8	24.2	22.6	NaN	27.5	25.0	0.0	2.5	0.00
REEP6	25.9	24.8	24.9	22.8	24.6	24.4	24.3	25.6	25.0	24.8	24.3	25.6	23.7	25.2	24.2	23.9	21.3	21.3	22.6	22.2	24.8	22.4	0.0	2.4	0.00
PAWR	28.6	28.5	26.9	27.9	28.8	28.4	28.6	28.4	28.7	28.7	28.3	27.5	28.0	28.8	27.2	25.3	25.6	26.6	26.6	21.7	28.5	26.1	0.0	2.4	0.00
CYB5R2	28.1	27.5	28.1	27.1	26.6	26.6	28.3	26.9	27.7	27.6	27.7	27.7	27.7	27.9	26.8	23.9	25.3	26.0	24.3	NaN	27.7	25.3	0.0	2.4	0.00
VSNL1	28.2	28.2	26.8	28.1	26.6	26.6	27.7	27.7	27.3	28.1	27.7	27.6	27.8	27.7	26.8	NaN	NaN	24.2	25.1	25.6	27.7	25.4	0.0	2.4	0.00
SALL2	31.2	31.3	31.3	31.7	31.9	32.4	31.2	31.1	31.4	31.5	31.4	31.0	30.9	31.7	30.9	28.8	28.7	30.1	29.2	27.5	31.3	29.0	0.0	2.3	0.00
CDCA7L	26.1	26.2	26.0	27.1	27.2	27.7	26.5	25.9	26.4	26.5	26.2	26.7	25.5	25.4	26.7	24.1	23.4	23.9	24.4	22.3	26.3	24.0	0.0	2.3	0.00
HPDL	27.6	26.7	23.3	28.7	27.6	27.6	27.8	27.1	27.6	27.2	26.7	26.9	25.4	26.9	24.9	NaN	22.4	22.2	26.9	25.3	27.2	24.9	0.0	2.3	0.00
SLC2A3	31.0	30.4	30.6	30.9	31.6	32.2	31.6	31.9	30.2	31.0	30.9	29.9	29.8	30.3	29.7	29.2	27.6	30.2	28.2	24.4	30.9	28.7	0.0	2.3	0.00
LCK	25.8	26.7	25.4	24.9	26.4	26.1	26.0	25.6	25.7	25.3	25.7	25.2	26.0	25.4	24.2	24.5	22.8	21.9	NaN	NaN	25.7	23.5	0.0	2.2	0.00
MDN1	32.0	31.5	30.7	31.7	31.4	31.5	31.0	31.1	31.1	31.6	31.0	31.5	31.4	31.3	30.4	27.9	29.1	29.2	30.3	29.2	31.4	29.2	0.0	2.2	0.00
EPPK1	28.6	27.7	27.1	28.2	27.8	28.4	28.5	28.7	27.3	29.0	28.2	27.6	26.6	27.2	25.8	25.8	26.6	24.4	27.8	24.5	28.0	25.8	0.0	2.2	0.00
KLHL7	26.4	27.1	26.4	27.0	26.7	27.0	26.4	26.9	26.8	26.9	26.2	26.9	26.0	26.8	25.6	24.0	24.0	25.9	25.3	22.9	26.8	24.6	0.0	2.2	0.00
CD2AP	28.1	27.9	27.2	27.8	28.2	28.6	28.1	28.1	28.1	28.0	27.7	27.5	27.6	28.2	27.2	25.2	25.9	25.9	26.6	24.7	28.0	25.9	0.0	2.2	0.00
BEND3	26.5	27.2	27.5	27.0	26.4	26.6	26.5	27.0	26.7	26.9	26.8	27.0	26.6	26.8	26.6	24.1	23.8	25.8	25.3	23.8	26.8	24.7	0.0	2.1	0.00
GFPT2	27.6	27.9	27.6	28.2	28.4	28.1	28.1	28.1	27.8	28.0	27.7	28.0	28.1	27.8	27.4	24.1	25.9	26.8	25.9	NaN	28.0	25.9	0.0	2.1	0.00
TTK	27.0	27.1	26.5	27.1	27.2	26.9	26.7	27.3	26.9	27.6	26.7	27.4	26.5	26.8	26.5	24.3	24.5	25.2	25.9	24.2	27.0	24.8	0.0	2.1	0.00
ITPK1	26.4	26.8	27.5	26.5	27.4	27.2	26.6	27.5	26.2	27.3	27.1	27.5	26.2	26.6	26.0	24.9	24.6	24.9	24.8	24.6	26.9	24.9	0.0	2.1	0.00
PLS1	27.2	27.6	27.1	27.2	27.9	27.7	27.7	27.6	27.8	27.5	27.0	27.1	26.8	27.3	26.0	22.8	25.3	24.6	25.4	26.0	27.4	25.3	0.0	2.1	0.00
HOOK1	24.1	24.0	24.3	25.0	25.2	24.6	24.6	24.9	24.9	24.5	24.4	23.4	24.3	23.8	23.0	NaN	22.5	NaN	21.9	NaN	24.4	22.5	0.0	1.9	0.00
ARG2	27.7	28.2	27.6	28.4	28.1	28.1	27.7	28.1	28.2	27.8	27.7	28.5	26.3	27.4	26.6	NaN	26.0	26.0	25.5	NaN	27.9	26.0	0.0	1.9	0.00
UGP2	33.0	32.9	32.9	33.3	33.0	33.4	33.3	33.1	33.3	33.0	32.7	32.6	33.0	32.9	31.7	30.9	30.8	31.3	31.3	30.6	33.0	31.1	0.0	1.9	0.00
TIPIN	24.0	24.7	21.5	19.6	22.2	21.0	22.2	24.0	NaN	21.0	23.1	23.1	23.9	23.8	22.4	21.5	21.2	NaN	20.9	20.7	23.1	21.2	0.0	1.9	0.00
SORL1	27.1	26.0	26.1	26.4	25.9	25.9	26.6	26.1	25.8	26.6	26.0	26.0	25.9	26.4	24.7	NaN	24.0	NaN	23.4	24.3	26.0	24.1	0.0	1.9	0.00
CTSC	31.1	30.7	30.9	30.6	30.8	30.8	31.2	31.1	31.1	31.1	30.7	31.1	30.9	30.8	30.6	28.7	28.2	29.9	29.4	28.7	30.9	29.1	0.0	1.9	0.00
FRAS1	26.0	25.7	25.1	26.0	25.9	25.7	26.0	25.6	25.7	25.4	25.5	25.7	24.9	25.8	25.0	23.3	23.8	23.9	24.3	22.8	25.7	23.8	0.0	1.8	0.00
SLC16A1	29.9	29.7	30.0	30.1	29.6	29.0	29.9	29.2	29.8	29.8	29.8	29.6	29.9	29.8	29.1	27.6	28.1	28.2	27.7	27.5	29.8	27.9	0.0	1.8	0.00
MID1IP1	26.9	27.5	26.8	27.1	27.0	27.3	26.7	27.7	27.0	27.2	26.6	27.2	26.4	27.2	26.3	22.5	24.4	25.3	25.2	NaN	27.0	25.2	0.0	1.8	0.00
SAMHD1	27.3	27.3	27.0	26.7	26.0	26.4	27.4	27.1	26.9	26.6	26.2	26.3	27.2	26.5	26.0	25.2	25.8	24.8	23.8	23.5	26.8	25.0	0.0	1.8	0.00
GULP1	26.5	27.4	26.2	27.6	26.8	27.0	26.4	26.9	26.7	26.3	25.6	26.8	25.2	26.9	25.3	23.7	23.3	25.6	24.9	25.0	26.7	24.9	0.0	1.8	0.00
PLK1	28.6	28.3	28.2	28.4	28.4	29.1	28.4	28.4	28.7	28.5	28.3	28.1	28.0	28.6	27.7	24.6	26.2	27.2	26.9	24.1	28.4	26.5	0.0	1.8	0.00
NQO2	27.1	27.1	26.1	25.4	27.4	29.5	29.0	28.8	26.8	26.4	26.8	25.5	27.1	27.1	26.0	25.6	26.4	24.6	24.1	25.0	27.1	25.3	0.0	1.8	0.00
CTSV	26.0	25.8	25.8	26.4	25.2	24.9	26.0	25.2	26.6	25.7	25.0	26.2	25.2	25.1	24.4	24.0	21.8	24.7	22.7	NaN	25.8	24.0	0.0	1.8	0.00
HIST1H1A	29.5	22.2	22.2	28.7	27.4	27.4	29.8	28.9	24.9	25.2	24.8	23.1	23.6	24.1	NaN	23.0	NaN	NaN	NaN	23.6	25.1	23.3	0.0	1.8	0.00
HELLS	30.1	29.9	29.3	30.0	29.9	30.5	29.9	29.8	29.8	29.8	29.7	30.0	29.1	29.8	29.3	27.4	27.8	28.4	28.5	26.7	29.9	28.1	0.0	1.8	0.00
ARAP1	27.1	27.2	26.2	26.6	26.6	27.4	26.6	26.9	26.4	27.1	26.4	26.5	26.5	26.6	26.2	24.5	25.7	25.1	23.9	24.4	26.6	24.8	0.0	1.8	0.00
RRAS2	29.1	28.9	28.5	29.4	28.5	28.7	28.8	29.0	28.7	28.9	28.6	29.0	29.1	29.1	27.7	25.9	26.4	27.8	27.5	26.8	28.9	27.1	0.0	1.8	0.00

MTHFS	23.4	25.2	21.3	23.8	24.0	23.6	24.5	24.6	23.4	22.3	21.9	24.6	22.4	26.0	22.1	20.7	21.7	22.5	22.4	21.3	23.7	21.9	0.0	1.8	0.00
HSD11B2	26.6	26.9	24.8	23.8	25.7	24.7	25.4	26.1	25.4	24.8	25.0	26.4	25.4	26.1	24.2	NaN	23.7	NaN	22.8	NaN	25.4	23.7	0.0	1.8	0.00
HIGD1A	26.2	26.1	26.3	26.3	27.9	28.3	26.2	25.8	26.8	25.0	25.5	26.4	24.9	26.1	25.9	24.8	26.2	24.1	23.9	22.5	26.2	24.4	0.0	1.8	0.00
BCAM	27.2	27.3	26.7	27.7	27.8	27.6	27.0	26.9	27.3	27.1	26.4	27.4	27.0	27.0	26.2	NaN	NaN	24.6	25.1	25.7	27.1	25.4	0.0	1.7	0.00
TJP2	29.4	29.3	28.6	29.1	30.0	29.3	29.4	29.3	28.9	29.0	28.6	28.6	28.3	29.3	28.1	26.2	27.1	27.4	28.1	27.6	29.2	27.5	0.0	1.7	0.00
HACL1	28.0	27.2	27.7	28.2	28.0	28.2	27.8	27.8	27.6	27.7	27.4	28.1	27.7	27.7	27.0	24.2	24.8	26.1	26.5	26.0	27.8	26.1	0.0	1.7	0.00
AURKB	27.3	28.0	26.8	26.6	27.4	27.2	26.5	27.7	27.2	27.5	27.3	27.1	26.9	27.5	26.7	24.3	25.7	25.4	26.0	24.6	27.2	25.5	0.0	1.7	0.00
GOLM1	26.7	26.6	26.8	27.6	27.1	27.3	27.1	26.7	26.6	27.1	26.4	27.0	26.1	26.6	25.6	25.2	24.8	26.0	25.0	24.7	26.7	25.1	0.0	1.7	0.00
INCENP	26.3	26.0	25.5	26.1	25.4	24.3	25.8	25.8	25.7	26.5	26.6	25.3	25.5	26.3	25.7	23.1	23.9	24.4	25.2	22.9	25.8	24.2	0.0	1.6	0.00
ABRACL	29.6	28.5	29.2	27.9	28.7	28.4	29.7	29.9	29.5	29.4	28.7	29.4	29.1	29.6	28.7	27.3	27.5	27.8	28.6	26.7	29.3	27.6	0.0	1.6	0.00
SLC9A3R1	29.9	29.7	29.3	30.1	29.8	29.7	29.9	30.1	29.9	29.7	29.7	29.3	29.3	29.8	29.1	28.1	27.9	28.9	28.2	26.5	29.8	28.1	0.0	1.6	0.00
TOP2A	32.1	31.9	31.8	32.1	32.0	32.0	31.9	31.8	32.0	32.3	32.1	32.0	31.9	31.9	31.5	30.0	30.0	31.0	30.7	29.4	32.0	30.3	0.0	1.6	0.00
Mar-01	25.2	25.1	25.5	26.3	24.3	25.7	25.5	25.2	25.6	25.4	24.8	26.5	24.8	25.5	24.4	23.8	24.5	23.3	23.8	NaN	25.4	23.8	0.0	1.6	0.00
LLGL2	25.4	25.3	23.0	25.4	25.1	25.3	25.0	25.1	25.3	24.8	24.7	25.7	24.6	24.9	23.7	NaN	NaN	NaN	23.3	NaN	25.1	23.5	0.0	1.6	0.00
SEPHS1	30.6	31.2	31.3	30.8	31.0	31.0	31.0	31.5	31.1	31.0	30.9	30.8	31.2	30.6	30.6	28.9	29.3	29.5	30.0	29.3	31.0	29.4	0.0	1.6	0.00
L2HGDH	27.3	27.0	26.6	27.1	26.5	26.7	26.9	26.9	27.4	26.9	27.2	27.2	27.5	27.0	26.8	25.3	25.4	25.2	25.5	26.2	27.0	25.4	0.0	1.6	0.00
SLC27A3	25.8	25.4	24.6	24.8	25.3	25.9	24.3	25.2	25.4	25.4	24.3	25.3	24.1	25.4	24.6	24.0	22.6	23.8	23.1	23.6	25.3	23.7	0.0	1.6	0.00
GJA1	29.5	29.2	29.0	29.5	29.5	29.4	29.4	29.1	29.5	29.6	29.3	29.3	28.8	29.5	29.0	27.5	27.6	28.9	28.1	26.4	29.4	27.8	0.0	1.6	0.00
FLVCR1	24.3	25.2	25.6	24.8	25.2	25.8	25.2	25.2	25.3	25.4	25.7	25.0	24.9	24.6	24.5	23.8	24.3	23.1	23.5	23.6	25.2	23.7	0.0	1.6	0.00
SLC38A2	27.9	28.0	27.2	28.2	28.2	28.2	27.4	27.8	28.0	27.8	27.6	28.0	26.3	27.9	26.9	25.5	25.8	27.5	27.0	23.2	27.9	26.3	0.0	1.6	0.00
DLGAP5	28.2	28.3	27.7	27.8	28.6	29.0	28.2	28.5	28.1	28.5	28.2	27.3	28.0	28.4	27.6	24.9	26.6	26.7	26.7	22.3	28.2	26.6	0.0	1.6	0.00
ZNF462	28.0	27.9	26.9	27.2	27.9	28.2	27.6	27.6	27.6	27.8	27.9	27.1	27.3	27.9	27.4	25.9	26.3	26.2	26.1	22.1	27.7	26.2	0.0	1.5	0.00
PARD6B	25.9	24.9	25.6	25.7	26.2	25.8	26.3	25.8	25.6	26.1	25.6	25.7	26.1	25.6	25.2	NaN	22.8	24.3	24.1	NaN	25.7	24.2	0.0	1.5	0.00
SKA3	25.9	25.6	24.6	25.0	25.8	25.3	25.2	26.0	25.3	26.1	25.9	25.4	23.9	25.7	25.7	23.4	22.7	24.0	24.0	NaN	25.5	24.0	0.0	1.5	0.00
AMT	25.5	24.6	24.7	25.7	25.1	24.9	25.5	24.5	25.2	24.5	24.9	25.7	24.2	24.8	24.3	23.1	23.4	23.9	22.8	NaN	24.9	23.4	0.0	1.5	0.00
KCTD14	24.6	24.8	25.0	25.5	24.5	24.8	25.6	25.0	24.9	24.7	24.8	24.9	24.8	24.7	23.4	NaN	NaN	23.3	23.3	NaN	24.8	23.3	0.0	1.5	0.00
VASP	29.2	28.6	28.8	28.4	28.9	29.0	29.3	28.5	28.9	29.3	28.7	28.3	28.5	29.2	28.3	27.5	27.2	28.3	27.3	26.3	28.9	27.4	0.0	1.5	0.00
HMGA1	31.4	31.5	31.4	32.3	32.0	32.2	31.6	30.9	31.8	31.6	32.1	31.4	31.8	31.9	31.6	28.1	30.3	31.0	30.1	27.7	31.7	30.2	0.0	1.5	0.00
CDC20	26.2	26.0	26.2	26.0	26.3	26.0	25.7	25.4	26.0	26.7	26.2	26.5	25.1	25.5	26.0	NaN	24.1	24.6	24.6	23.7	26.0	24.6	0.0	1.5	0.00
OAT	31.9	31.5	31.5	31.4	31.6	31.4	32.2	32.1	31.6	31.7	31.7	31.6	31.4	32.1	31.0	29.9	30.8	30.3	30.0	30.0	31.6	30.2	0.0	1.5	0.00
RIF1	29.2	29.5	29.3	28.9	28.8	29.2	29.5	29.5	29.2	29.3	28.8	28.9	28.6	29.2	28.9	26.4	27.4	27.9	28.8	27.6	29.2	27.7	0.0	1.5	0.00
CBR1	31.2	31.1	30.1	30.7	30.2	30.5	30.9	30.9	30.3	31.0	30.7	29.5	30.8	31.0	29.1	29.1	29.5	28.8	29.9	29.9	30.8	29.3	0.0	1.4	0.00
DNMT3A	30.1	29.6	29.8	29.5	29.9	29.3	29.3	29.5	29.6	29.9	29.7	30.3	29.7	30.0	29.6	28.3	28.2	28.2	28.3	28.0	29.7	28.3	0.0	1.4	0.00
MGME1	27.4	27.5	26.8	27.3	26.9	27.1	27.0	27.6	27.3	27.4	26.7	27.5	26.6	27.3	26.8	25.2	26.1	25.6	26.1	25.3	27.3	25.8	0.0	1.4	0.00
FKBP11	26.4	27.2	25.7	27.3	26.9	26.5	26.5	27.1	27.0	26.5	26.3	27.3	NaN	26.5	23.8	25.1	26.2	NaN	25.0	26.3	26.5	25.1	0.0	1.4	0.00
AKAP1	27.4	27.0	25.6	27.1	27.8	27.6	27.0	27.2	27.7	27.2	27.1	25.8	26.4	27.5	26.1	25.7	25.4	26.0	24.4	NaN	27.1	25.7	0.0	1.4	0.00
UNG	25.6	25.8	26.4	26.7	26.1	26.7	26.2	26.2	26.6	26.3	26.2	26.9	25.7	25.9	26.3	23.8	24.1	25.6	25.4	23.9	26.2	24.8	0.0	1.4	0.00
NACC1	27.8	27.9	27.6	28.3	28.0	28.7	27.9	27.7	27.8	27.9	27.9	27.5	27.6	27.8	27.3	26.0	25.4	27.1	26.6	26.3	27.9	26.4	0.0	1.4	0.00
RRM2B	26.8	26.1	26.8	26.4	25.2	25.4	26.8	26.9	26.2	26.6	26.5	26.4	26.8	26.0	25.2	24.5	24.5	25.6	25.7	24.9	26.5	25.0	0.0	1.4	0.00
NSUN5	27.0	27.2	26.9	27.5	27.5	27.5	26.9	27.0	27.2	26.9	26.9	27.6	26.8	26.9	26.6	24.1	25.4	25.7	26.4	25.5	27.0	25.6	0.0	1.4	0.00
ITGA6	28.4	28.5	29.3	28.9	28.5	28.3	28.3	28.4	28.7	28.9	28.7	28.9	28.4	28.2	28.1	27.3	26.0	27.8	26.9	26.7	28.5	27.1	0.0	1.4	0.00
ESRP1	29.4	29.3	29.1	29.2	29.4	29.3	29.3	29.3	29.4	28.9	28.8	29.4	29.0	29.1	27.9	28.1	28.0	27.7	27.7	27.8	29.3	27.9	0.0	1.4	0.00

RAB3B	27.4	26.8	27.4	27.7	26.9	27.3	27.7	26.9	27.4	27.2	27.0	27.2	27.2	27.1	26.0	25.9	25.6	26.3	25.7	24.0	27.2	25.8	0.0	1.4	0.00
SLC2A1	29.7	29.8	29.3	29.6	30.0	29.7	29.2	29.6	29.2	29.7	29.9	30.0	29.5	30.3	29.3	27.5	26.8	29.1	29.1	27.1	29.7	28.3	0.0	1.4	0.00
NPM3	29.6	29.4	28.9	29.5	30.2	30.5	29.5	29.0	29.8	29.7	29.4	29.2	29.1	29.5	29.2	27.2	29.2	27.8	27.7	28.4	29.5	28.1	0.0	1.4	0.00
GNL3L	26.6	26.0	26.2	27.1	26.8	26.6	26.4	26.2	26.7	26.5	26.4	26.0	25.9	26.7	25.9	25.1	25.1	25.1	24.9	25.1	26.5	25.1	0.0	1.4	0.00
BSG	31.1	31.0	31.4	31.5	30.7	30.4	31.0	30.9	31.0	31.2	30.9	31.2	30.8	31.1	30.5	29.0	29.6	29.6	29.6	29.0	31.0	29.6	0.0	1.4	0.00
ANK3	27.8	27.9	26.9	27.2	27.8	27.8	27.9	28.4	27.6	26.9	26.8	26.9	27.3	27.6	26.8	26.3	26.6	26.1	25.5	25.3	27.6	26.2	0.0	1.4	0.00
KIAA0391	26.8	27.4	26.4	25.7	26.9	26.5	26.7	27.2	26.4	26.5	26.7	26.8	26.6	26.6	25.7	24.7	26.0	24.9	24.7	25.8	26.7	25.3	0.0	1.4	0.00
ZNF281	25.5	25.9	25.8	26.4	26.8	26.1	25.5	25.1	26.2	26.2	26.0	25.8	25.4	25.6	25.4	24.0	24.1	24.5	24.8	NaN	25.9	24.5	0.0	1.4	0.00
SLC39A14	27.9	28.0	28.1	28.8	27.8	28.0	27.5	27.8	27.6	28.1	27.6	28.6	26.9	27.7	26.9	NaN	25.3	26.6	26.5	24.4	27.9	26.5	0.0	1.4	0.00
KIF2C	28.9	28.9	28.4	28.6	28.9	28.8	29.0	29.1	29.0	29.1	28.9	28.3	28.8	28.9	28.4	26.6	27.4	27.8	27.6	26.0	28.9	27.5	0.0	1.3	0.01
CPVL	27.1	26.6	26.4	27.1	27.2	27.4	27.5	27.2	26.7	26.7	26.5	27.1	26.8	26.6	26.4	25.6	26.2	25.6	25.0	24.2	26.9	25.6	0.0	1.3	0.01
BAZ2A	28.8	29.3	28.9	27.7	30.0	30.0	29.0	28.6	29.7	28.7	28.9	29.0	28.3	29.0	28.6	27.9	28.9	27.0	27.4	27.1	29.0	27.6	0.0	1.3	0.01
PWWP2A	24.8	24.0	24.4	25.1	25.0	25.1	24.6	24.7	24.2	23.9	24.7	24.4	23.7	23.8	23.7	23.0	NaN	23.3	22.6	NaN	24.5	23.2	0.0	1.3	0.01
TGIF1	24.3	24.5	23.7	24.3	23.4	23.5	23.6	24.4	24.4	24.3	23.6	24.1	24.0	23.8	23.3	23.2	22.1	22.7	22.6	NaN	24.0	22.7	0.0	1.3	0.01
TRMT1	27.1	27.9	27.0	27.9	28.3	27.9	27.3	27.6	27.8	27.1	27.3	27.6	27.6	27.6	27.3	25.9	26.8	26.4	26.2	26.1	27.6	26.3	0.0	1.3	0.01
ALDH1B1	29.6	29.6	29.3	30.4	30.1	30.1	29.9	29.8	29.7	29.4	29.1	29.8	29.4	29.7	29.1	28.3	29.3	28.5	28.3	27.5	29.7	28.4	0.0	1.3	0.01
CRABP2	28.7	28.5	27.5	28.6	28.6	28.9	28.9	28.7	28.7	28.9	28.7	28.0	28.8	28.6	29.2	27.1	27.9	27.7	26.9	26.1	28.7	27.4	0.0	1.3	0.01
CEBPZ	29.3	29.3	29.4	29.3	29.1	28.7	29.2	28.9	29.4	29.4	29.1	29.2	28.9	29.0	29.1	26.8	27.8	28.1	28.0	27.8	29.2	27.9	0.0	1.3	0.01
CBS	32.1	32.1	32.1	32.0	32.6	32.4	32.1	32.2	32.4	31.9	32.0	31.9	32.1	32.0	31.5	29.6	30.8	30.8	30.8	29.4	32.1	30.8	0.0	1.3	0.01
PDK3	26.1	26.4	25.3	25.6	25.0	25.2	26.0	26.5	25.5	25.7	25.7	26.0	25.8	24.8	25.4	23.6	24.8	23.9	24.4	NaN	25.7	24.4	0.0	1.3	0.01
LPCAT1	29.6	29.3	29.3	29.2	29.1	29.1	29.3	29.5	29.4	29.3	29.1	29.4	28.8	29.5	28.6	28.3	27.7	28.5	27.7	27.1	29.3	28.0	0.0	1.3	0.01
SLC3A2	31.8	31.9	31.3	33.2	32.3	31.8	31.8	32.5	32.2	31.7	31.5	32.6	31.3	32.3	31.5	29.8	30.8	31.0	30.4	28.9	31.9	30.6	0.0	1.3	0.01
KPNA2	32.6	32.5	32.7	32.6	32.9	33.0	32.6	32.6	32.8	32.6	32.5	32.6	32.3	32.4	32.1	30.6	30.8	31.8	31.8	30.2	32.6	31.3	0.0	1.3	0.01
SLC25A29	26.7	26.9	26.8	25.5	26.3	25.8	26.2	26.5	26.7	26.2	26.2	27.7	24.9	26.3	25.9	25.0	25.0	24.4	26.0	24.9	26.3	25.0	0.0	1.3	0.01
ALDH4A1	28.1	28.4	28.4	28.2	28.5	28.7	28.0	28.2	28.5	28.1	27.8	28.4	28.2	28.4	28.0	27.3	26.4	27.0	26.6	27.0	28.3	27.0	0.0	1.3	0.01
HMOX1	25.9	26.3	28.0	27.2	26.8	26.6	26.0	26.3	26.4	26.8	26.5	27.5	25.2	26.3	26.9	22.9	24.8	26.0	25.2	25.2	26.5	25.2	0.0	1.3	0.01
CCNB1	28.6	28.8	28.6	28.9	29.0	29.2	28.6	28.4	28.7	28.5	28.5	28.8	28.4	28.6	28.5	26.6	26.9	28.3	27.9	26.0	28.6	27.4	0.0	1.3	0.01
TGS1	24.4	25.1	23.9	24.8	24.2	25.5	24.2	25.6	24.8	25.1	25.0	24.6	25.0	24.9	24.8	22.9	23.6	23.5	NaN	24.1	24.8	23.6	0.0	1.2	0.01
ACSL4	29.3	29.4	29.4	29.5	28.9	28.4	28.8	29.6	28.8	29.5	29.3	29.5	28.9	29.0	28.3	27.3	27.8	28.9	28.8	27.0	29.3	28.1	0.0	1.2	0.01
SLC38A1	27.4	27.3	27.7	28.2	26.8	28.1	27.1	27.0	27.3	27.6	27.4	27.7	26.7	26.9	26.4	24.7	25.8	26.8	27.3	25.0	27.3	26.1	0.0	1.2	0.01
ANLN	26.5	26.7	25.7	26.6	26.4	26.5	25.7	26.8	25.7	26.9	26.8	26.1	25.2	27.0	26.2	24.8	24.6	25.5	25.6	25.1	26.5	25.3	0.0	1.2	0.01
QSER1	24.8	24.5	25.9	24.0	26.3	25.7	24.3	24.6	25.0	25.2	25.0	25.2	24.8	24.5	25.1	23.5	23.2	NaN	23.7	24.1	24.9	23.7	0.0	1.2	0.01
HERC2	25.2	25.4	26.2	25.8	26.1	25.8	25.9	25.2	25.4	25.9	25.5	26.6	26.1	25.9	25.0	24.3	24.2	25.0	24.1	24.9	25.8	24.6	0.0	1.2	0.01
TRIM24	28.2	28.2	27.8	28.5	28.0	28.1	28.4	28.0	28.4	28.3	28.2	27.9	27.9	28.0	27.6	26.7	26.8	27.8	27.0	25.9	28.1	26.9	0.0	1.2	0.01
TACC3	27.6	27.6	27.6	28.0	28.1	27.8	27.5	27.6	27.7	28.0	28.0	27.6	27.5	27.8	27.7	25.5	25.9	27.4	27.0	25.0	27.6	26.4	0.0	1.2	0.01
TRIP10	26.0	25.9	26.1	25.9	26.0	26.8	26.1	26.3	26.5	26.3	26.1	26.1	26.4	26.3	25.7	25.2	24.6	25.1	24.8	24.5	26.1	24.9	0.0	1.2	0.01
USP28	26.0	25.4	26.3	26.2	25.8	25.8	26.3	26.7	25.0	25.2	25.7	25.0	25.9	25.6	24.8	NaN	NaN	25.0	24.4	24.1	25.8	24.6	0.0	1.2	0.01
EPHX1	29.2	29.1	28.4	29.2	28.9	29.2	29.1	28.9	28.7	28.8	28.4	29.2	28.7	28.9	27.8	27.6	28.6	27.9	27.4	27.1	28.9	27.7	0.0	1.2	0.01
FGFR1OP	25.7	25.7	25.4	22.9	26.3	26.7	24.6	26.2	26.2	25.4	25.7	25.6	25.0	26.0	25.6	25.0	24.0	25.1	23.7	23.8	25.7	24.5	0.0	1.2	0.01
KIF22	27.2	28.0	27.0	27.7	27.7	27.9	27.0	27.5	27.5	28.1	27.7	27.5	27.3	27.6	27.8	26.2	26.0	26.5	27.1	25.2	27.5	26.3	0.0	1.2	0.01
LRBA	28.5	28.4	28.3	28.3	28.5	28.4	28.6	28.5	28.5	28.6	28.1	28.2	29.1	28.3	27.4	25.6	27.2	26.7	27.5	27.3	28.4	27.2	0.0	1.2	0.01
PSIP1	31.3	31.2	31.3	31.0	30.9	30.9	31.4	31.3	31.1	31.4	31.2	31.2	31.2	31.2	31.1	30.1	30.1	30.0	29.9	29.4	31.2	30.0	0.0	1.2	0.01

ZIC2	25.5	26.4	25.8	26.1	26.8	26.8	25.6	25.2	26.3	26.6	26.3	26.5	25.7	25.7	26.1	25.0	25.1	25.0	23.9	NaN	26.2	25.0	0.0	1.2	0.01
PCCA	27.8	27.7	27.2	27.3	27.8	28.1	27.6	27.7	27.6	27.6	27.6	27.2	27.8	27.7	27.4	26.5	26.9	26.3	25.9	26.5	27.7	26.5	0.0	1.2	0.01
C11orf98	26.6	26.9	24.9	25.8	27.5	27.8	26.0	26.8	27.3	25.9	25.9	26.4	25.8	27.3	25.9	25.5	25.7	24.4	25.1	24.7	26.5	25.3	0.0	1.2	0.01
ENPP1	26.5	26.2	25.2	25.8	26.1	25.6	26.1	26.6	25.7	26.1	25.6	26.3	25.5	26.4	24.9	24.6	NaN	24.2	25.0	25.6	26.1	24.9	0.0	1.2	0.01
MTFP1	25.5	25.5	24.6	25.1	24.1	24.1	23.4	24.3	25.1	25.1	25.2	25.0	24.8	25.6	23.9	NaN	24.4	23.4	24.1	23.7	25.0	23.9	0.0	1.2	0.01
ACAD8	27.2	26.8	26.5	27.0	26.2	26.0	26.9	26.3	26.8	26.9	26.7	27.8	26.8	26.7	26.4	25.8	24.4	24.8	25.4	26.2	26.8	25.6	0.0	1.2	0.01
GLA	27.1	27.2	26.6	26.9	27.0	27.4	27.5	27.4	27.2	27.2	26.6	27.4	26.9	26.7	26.3	25.9	25.9	25.9	26.1	26.2	27.2	26.0	0.0	1.2	0.01
POLRMT	26.2	27.2	26.6	28.0	27.1	27.3	26.5	27.0	26.8	27.0	26.9	28.2	25.7	26.9	26.6	22.9	25.7	25.9	26.4	24.7	27.0	25.8	0.0	1.2	0.01
FDXR	27.7	27.5	26.4	27.5	28.1	27.8	27.5	27.8	27.2	27.3	27.2	27.6	27.0	26.8	26.5	26.9	25.3	25.3	26.2	27.3	27.5	26.3	0.0	1.2	0.01
ZFAND6	24.6	25.0	24.9	24.9	25.8	24.9	26.1	24.2	25.5	25.0	24.9	24.8	24.6	25.1	24.5	24.1	23.7	23.2	23.8	23.7	24.9	23.8	0.0	1.2	0.01
NASP	33.1	33.0	33.1	33.1	33.2	33.2	33.3	33.2	33.4	33.2	33.2	32.9	33.1	33.0	33.2	31.4	31.7	32.3	32.4	31.7	33.2	32.0	0.0	1.2	0.01
ABHD17B	25.5	26.2	25.5	25.9	26.6	25.8	25.4	26.5	25.7	25.8	25.8	26.0	25.4	25.3	25.1	24.6	24.0	24.7	24.6	24.4	25.8	24.6	0.0	1.1	0.01
ERCC6L	26.6	25.9	26.2	26.3	26.2	25.9	26.6	25.6	26.3	26.5	26.5	25.3	26.5	25.8	25.2	24.1	25.1	25.5	25.0	24.1	26.2	25.1	0.0	1.1	0.01
PRKCI	27.7	27.4	27.7	27.2	27.6	28.2	27.7	27.6	27.8	28.0	27.8	27.9	27.8	27.4	27.4	26.5	26.8	26.6	26.2	25.9	27.7	26.6	0.0	1.1	0.01
ARFGEF1	28.1	28.3	27.9	28.2	28.1	28.2	28.1	28.4	28.0	28.1	28.1	27.9	27.7	27.8	27.2	27.0	27.1	26.8	26.8	26.3	28.1	26.9	0.0	1.1	0.01
KIF11	28.9	29.7	28.6	29.1	29.2	29.2	29.0	29.5	29.1	29.2	29.2	29.0	29.0	29.5	29.0	27.2	27.8	28.2	28.5	26.7	29.1	28.0	0.0	1.1	0.01
CHML	25.3	25.3	25.5	23.6	26.4	26.2	25.1	24.9	25.6	23.8	24.9	23.9	25.4	24.8	24.7	24.5	23.5	23.9	24.1	24.0	25.2	24.1	0.0	1.1	0.02
COA7	26.5	26.2	27.0	27.9	25.4	26.8	27.0	26.8	25.6	27.0	26.6	26.8	26.1	26.5	26.4	23.6	25.2	25.7	26.5	25.4	26.7	25.6	0.0	1.1	0.02
HTATIP2	23.7	25.8	23.8	22.3	23.7	23.2	24.7	26.0	23.8	22.6	25.2	26.1	24.0	25.1	22.8	NaN	22.6	NaN	22.9	NaN	23.9	22.8	0.0	1.1	0.02
ANKZF1	26.7	25.7	25.8	26.2	26.1	25.1	26.1	26.7	26.2	26.4	26.5	26.2	26.1	26.2	26.1	25.0	24.9	25.5	25.1	24.7	26.2	25.0	0.0	1.1	0.02
PAK1	27.8	28.5	27.3	27.4	27.6	28.0	27.9	28.6	27.5	27.8	28.1	27.2	28.1	28.2	28.1	26.1	26.6	27.6	26.8	26.6	27.8	26.7	0.0	1.1	0.02
MTA3	27.8	27.9	27.6	27.1	28.2	28.3	27.7	27.3	27.8	27.1	27.9	27.5	27.8	27.7	27.0	26.1	26.8	25.7	26.8	26.5	27.7	26.6	0.0	1.1	0.02
EIF1B	28.3	27.9	28.4	27.1	28.3	27.8	28.0	28.1	28.2	28.7	28.3	28.5	28.0	27.8	28.3	27.3	26.8	27.7	26.8	26.9	28.2	27.1	0.0	1.1	0.02
FAM207A	25.2	25.5	24.1	24.7	24.7	25.8	24.5	26.5	24.8	25.3	25.3	24.9	24.1	26.0	24.1	23.8	24.0	NaN	24.5	23.7	25.1	24.0	0.0	1.1	0.02
SLC4A7	27.3	27.5	25.3	25.7	27.0	26.5	26.6	27.8	27.0	26.5	27.0	26.7	27.4	27.1	26.8	25.5	26.3	24.5	26.2	25.4	27.0	25.9	0.0	1.1	0.02
JMJD1C	23.6	25.1	NaN	23.9	22.9	24.1	23.8	24.0	23.2	23.0	24.5	23.6	24.2	23.6	22.6	NaN	23.0	NaN	NaN	22.7	23.8	22.7	0.0	1.1	0.02
PNPLA6	25.6	25.9	24.7	25.2	25.0	26.7	25.1	25.6	25.3	25.4	25.2	24.8	24.1	24.4	24.7	24.1	24.0	24.0	24.2	24.0	25.2	24.1	0.0	1.1	0.02
GLTSCR2	26.6	26.9	27.4	26.9	28.0	28.8	26.9	26.7	27.5	27.1	26.9	26.1	26.4	26.2	27.7	25.2	26.0	24.9	26.1	25.6	26.9	25.8	0.0	1.1	0.02
PYCR2	28.9	29.3	28.4	29.4	29.4	29.4	28.7	29.0	29.0	28.9	28.9	28.9	29.1	29.0	28.8	27.5	28.5	28.0	27.8	27.6	29.0	27.9	0.0	1.1	0.02
SMARCD2	27.4	27.5	26.6	27.0	26.9	27.3	27.4	28.3	27.5	27.5	27.0	26.4	27.3	27.4	26.3	25.7	26.9	25.2	26.2	27.7	27.3	26.2	0.0	1.1	0.02
FZD7	26.4	25.8	26.1	27.4	26.5	26.3	26.7	25.9	26.4	25.8	25.8	26.5	25.4	26.0	25.7	25.7	24.4	25.6	24.6	24.5	26.2	25.1	0.0	1.1	0.02
GOLPH3L	25.2	25.3	24.7	25.8	24.6	24.6	25.6	25.9	25.5	24.8	25.5	24.5	24.7	25.6	25.2	23.9	24.1	24.1	24.8	24.1	25.2	24.1	0.0	1.1	0.02
KNOP1	27.4	27.5	27.1	27.6	27.5	27.5	27.4	27.2	27.5	27.7	27.5	27.4	27.1	27.5	27.4	25.7	26.6	26.3	26.7	25.9	27.5	26.4	0.0	1.1	0.02
EIF4EBP1	25.7	25.8	24.2	26.1	26.9	27.3	25.6	26.4	26.1	25.7	25.5	25.6	25.7	26.4	25.6	24.0	25.6	24.0	24.4	25.0	25.8	24.7	0.0	1.1	0.02
DDI2	26.7	26.8	26.8	26.6	27.1	27.0	26.4	27.5	26.7	26.8	26.8	26.2	26.6	26.8	26.7	24.6	26.2	25.6	25.6	25.7	26.8	25.7	0.0	1.1	0.02
TIMMDC1	25.8	25.4	25.9	25.7	25.9	26.4	24.3	24.8	24.5	25.3	24.8	25.9	24.4	25.5	24.6	23.8	24.1	24.8	24.9	24.2	25.5	24.4	0.0	1.1	0.02
PSAT1	32.8	32.8	33.3	33.1	33.0	32.9	32.6	33.4	33.0	32.7	32.9	32.8	33.2	33.0	32.6	31.1	31.8	31.8	32.1	31.9	32.9	31.9	0.0	1.1	0.02
UBE2S	28.0	27.8	28.1	28.1	28.0	28.2	28.1	27.9	28.1	28.3	27.9	27.7	27.4	27.7	27.4	25.5	26.4	27.6	27.5	25.7	28.0	26.9	0.0	1.1	0.02
PTCD1	24.5	25.1	24.6	25.8	23.5	24.2	24.5	25.1	25.0	25.1	24.0	24.9	23.2	23.8	24.8	22.8	23.2	23.5	24.0	23.5	24.6	23.5	0.0	1.1	0.02
HLA-C	25.9	24.9	24.7	25.4	25.1	23.6	25.0	23.7	25.8	25.6	25.2	25.5	23.5	24.1	24.1	23.1	NaN	24.2	24.0	23.4	25.0	24.0	0.0	1.1	0.02
SLC7A5	28.1	27.7	27.5	29.6	29.0	28.5	27.7	27.9	28.4	27.8	28.1	28.3	27.7	28.6	27.4	25.8	27.0	27.4	27.1	25.0	28.1	27.0	0.0	1.1	0.02
ACOT8	25.6	25.2	25.2	26.5	25.7	26.3	25.7	25.9	25.9	24.7	24.7	25.8	24.6	25.5	24.8	25.2	24.5	24.4	24.4	24.7	25.6	24.6	0.0	1.1	0.02

TSR1	28.6	29.4	29.4	28.7	28.7	28.9	28.7	29.5	29.3	29.3	28.8	29.4	28.9	28.8	28.7	27.2	27.9	27.8	28.4	27.5	28.9	27.8	0.0	1.0	0.02
ARHGAP19	23.0	24.6	21.5	NaN	25.2	24.5	21.4	23.2	22.8	22.3	22.6	NaN	21.7	23.4	21.7	22.0	NaN	NaN	NaN	NaN	22.9	21.8	0.0	1.0	0.02
AUH	24.9	25.5	24.4	24.4	25.1	26.0	25.3	26.1	25.4	24.9	24.6	25.4	24.2	24.6	24.5	23.9	23.8	25.1	24.1	23.9	25.0	24.0	0.0	1.0	0.02
MIS18A	24.7	24.5	25.1	24.9	25.5	26.2	25.2	25.8	24.7	25.3	24.9	25.0	24.7	24.5	24.2	NaN	NaN	23.9	23.9	NaN	25.0	23.9	0.0	1.0	0.02
BLVRB	27.0	26.6	26.5	26.9	26.4	27.0	27.8	27.3	26.6	26.7	26.5	26.5	26.6	27.0	25.7	26.5	25.4	25.5	25.5	26.0	26.6	25.6	0.0	1.0	0.02
PLCB3	29.7	29.7	29.7	29.5	30.2	30.3	29.5	29.6	29.6	29.5	29.6	29.4	29.3	29.5	28.7	28.5	28.7	28.3	28.1	28.6	29.6	28.6	0.0	1.0	0.02
ASNS	30.4	30.7	31.8	31.4	31.0	30.7	30.2	31.6	31.0	31.0	30.8	31.4	31.1	30.6	31.0	28.8	29.6	30.3	30.6	29.5	31.0	30.0	0.0	1.0	0.03
PYCR1	29.4	29.4	28.9	29.5	29.6	29.9	29.1	29.4	29.6	29.5	29.4	29.4	29.7	29.3	29.0	28.3	29.6	28.5	28.3	28.3	29.4	28.4	0.0	1.0	0.03
MSH2	31.4	31.2	31.4	31.2	31.4	31.4	31.2	31.4	31.3	31.3	31.2	31.2	31.3	31.4	31.0	29.6	30.2	30.4	30.5	29.7	31.3	30.3	0.0	1.0	0.03
MAN2A1	28.2	27.8	27.8	28.6	28.0	27.5	28.1	28.3	27.9	28.2	27.8	28.1	28.0	28.1	26.8	26.0	27.1	27.2	27.0	27.2	28.1	27.1	0.0	1.0	0.03
CUTC	25.4	26.0	25.7	25.8	26.1	26.5	25.4	26.2	25.8	25.3	25.7	25.1	25.8	25.7	25.1	25.5	24.6	24.9	24.6	24.2	25.7	24.7	0.0	1.0	0.03
FADD	28.6	28.4	28.5	27.6	28.0	28.0	28.7	29.0	28.4	28.6	28.1	27.9	28.2	28.4	28.3	27.5	26.5	27.2	27.4	27.3	28.4	27.4	0.0	1.0	0.03
JUP	29.9	30.1	29.9	30.0	29.8	30.2	29.7	29.9	29.9	29.8	29.9	30.1	29.4	29.7	29.2	28.2	28.8	29.1	29.0	27.5	29.9	28.9	0.0	1.0	0.03
PHGDH	33.7	33.7	33.6	33.7	34.0	34.3	33.6	34.0	33.9	33.6	33.6	33.4	33.9	33.8	33.5	31.9	33.0	32.6	32.8	32.5	33.7	32.7	0.0	1.0	0.03
POLR2L	27.0	25.9	26.8	26.6	26.9	27.2	26.5	27.2	26.2	25.8	25.9	26.5	25.9	26.3	26.4	26.3	25.3	23.9	25.7	25.1	26.5	25.5	0.0	1.0	0.03
TRABD	26.0	26.0	25.5	25.8	26.3	26.2	25.6	25.0	25.7	25.8	25.6	25.7	24.6	25.7	25.2	23.4	25.1	24.5	24.4	25.0	25.7	24.7	0.0	1.0	0.03
HK2	29.6	30.0	30.4	29.8	29.7	29.4	29.6	29.8	29.8	29.8	29.8	29.8	29.8	29.8	29.5	27.2	28.6	29.4	28.9	26.6	29.8	28.8	0.0	1.0	0.03
RCOR2	27.3	27.2	27.3	27.8	27.5	27.2	27.2	26.7	27.5	27.3	27.3	27.3	27.1	27.1	27.2	26.3	26.3	26.6	26.1	25.0	27.3	26.3	0.0	1.0	0.03
PDCD2L	26.4	26.7	26.6	27.3	26.9	26.7	26.9	27.1	26.8	26.8	26.5	26.8	26.1	26.8	26.1	24.9	25.6	26.0	26.3	24.9	26.8	25.8	0.0	1.0	0.03
CDC123	27.8	28.0	28.4	27.9	28.1	28.2	27.9	28.5	27.8	28.0	27.8	28.2	27.2	27.7	27.7	26.3	26.5	27.4	27.5	26.4	27.9	27.0	0.0	1.0	0.03
NFXL1	25.1	25.6	25.9	26.7	26.4	26.1	25.5	25.7	25.8	25.3	25.1	26.2	25.0	24.3	25.2	23.9	25.2	24.7	24.6	23.4	25.6	24.6	0.0	1.0	0.03
GCLC	25.6	26.1	26.2	26.6	25.9	26.2	26.3	26.3	26.0	25.7	26.3	25.7	26.2	26.2	25.4	24.9	25.0	25.9	25.9	24.9	26.2	25.2	0.0	1.0	0.03
STRBP	28.9	29.0	29.0	29.2	28.4	28.5	28.7	29.0	28.8	29.2	28.9	28.8	28.6	28.6	28.4	27.4	28.1	27.9	27.8	26.7	28.8	27.8	0.0	1.0	0.03
NDC1	27.3	27.1	27.2	27.5	27.6	27.8	27.2	27.1	27.3	27.2	27.4	27.5	27.0	27.1	27.2	26.0	26.3	26.0	26.6	26.2	27.3	26.3	0.0	1.0	0.03
ZNF687	25.7	25.3	24.8	25.3	25.3	25.7	25.4	25.0	24.8	25.6	25.6	24.5	25.9	25.5	24.8	24.3	24.0	24.7	24.5	24.1	25.4	24.4	0.0	1.0	0.03
GPT2	28.2	28.2	28.5	29.4	28.5	28.4	27.7	28.5	28.5	28.4	28.0	28.9	27.9	28.5	28.0	27.0	26.9	27.5	27.7	27.4	28.4	27.5	0.0	1.0	0.03
HLA-A	24.7	23.9	NaN	24.2	25.9	24.0	24.1	22.8	24.7	24.3	24.2	24.5	24.1	24.0	23.2	22.8	NaN	NaN	23.4	NaN	24.2	23.2	0.0	1.0	0.03
WNK3	24.0	24.4	22.9	23.7	23.7	24.1	23.3	25.1	23.4	23.6	23.8	NaN	23.0	23.7	22.8	NaN	23.2	23.7	22.8	22.1	23.7	22.8	0.0	1.0	0.03
ZNF483	27.0	26.7	25.6	25.7	26.0	26.0	26.6	26.6	26.3	26.0	26.7	25.5	25.1	26.5	25.0	25.3	25.4	23.9	22.8	25.9	26.2	25.2	0.0	1.0	0.03
MRPL17	27.8	27.8	27.4	27.8	27.6	28.1	27.8	27.8	27.7	27.8	27.0	27.8	27.4	27.3	27.7	26.3	27.6	26.7	26.9	26.8	27.8	26.8	0.0	1.0	0.03
RPUSD2	26.3	26.3	26.5	26.4	26.1	26.0	26.6	27.0	26.4	26.4	26.5	26.6	26.2	26.7	26.3	24.8	25.4	25.2	26.0	25.5	26.4	25.5	0.0	1.0	0.03
LYN	27.3	28.0	27.0	27.3	28.2	28.1	26.6	27.7	26.6	27.0	27.1	27.1	27.2	27.8	26.3	27.0	27.2	25.8	26.2	25.9	27.2	26.3	0.0	1.0	0.03
CHEK2	25.4	26.3	25.5	26.9	26.3	26.0	25.8	24.5	25.8	26.4	26.0	24.6	26.0	25.6	25.7	23.7	24.5	25.5	24.6	25.2	25.9	24.9	0.0	1.0	0.03
RRS1	29.5	29.8	28.6	29.6	29.9	30.2	29.3	29.6	29.6	29.5	29.2	29.6	28.7	29.5	29.3	27.2	28.7	27.8	28.6	28.7	29.6	28.6	0.0	1.0	0.03
DNAJB5	24.3	24.9	25.4	24.9	25.1	26.1	24.7	23.9	24.8	25.8	25.0	23.3	24.3	24.6	24.6	23.7	24.1	24.6	23.3	23.6	24.9	23.9	0.0	0.9	0.03
TIMELESS	26.3	26.7	25.7	26.5	26.1	26.4	26.3	26.5	26.3	26.3	26.5	26.3	26.1	26.2	26.3	24.8	25.0	25.7	25.8	24.5	26.3	25.4	0.0	0.9	0.04
TFB1M	26.3	26.8	26.2	26.7	26.8	26.9	26.2	26.1	25.9	26.5	26.1	25.6	26.2	25.8	25.5	24.6	25.7	25.9	24.4	25.0	26.2	25.3	0.0	0.9	0.04
PASK	24.6	24.4	24.4	24.2	24.0	24.3	24.5	24.6	24.2	24.4	24.9	23.6	25.2	24.9	23.5	24.1	23.2	23.7	NaN	22.9	24.4	23.5	0.0	0.9	0.04
HSD17B4	32.4	32.4	32.2	32.2	32.2	32.6	32.5	32.4	32.3	32.2	32.2	32.3	32.3	32.4	31.8	31.7	31.3	31.4	31.3	30.9	32.3	31.4	0.0	0.9	0.04
DDX18	30.6	30.5	30.0	30.2	30.6	30.6	29.7	30.4	30.1	30.3	29.9	30.9	29.6	30.1	29.9	28.6	29.4	29.2	29.8	29.1	30.2	29.3	0.0	0.9	0.04
PPM1B	28.6	28.3	28.2	28.7	29.3	29.5	29.0	29.1	28.7	28.2	28.2	28.0	28.4	28.7	28.2	27.8	27.7	28.1	27.6	27.3	28.7	27.7	0.0	0.9	0.04
RNF114	27.5	27.0	26.4	26.7	26.4	26.9	27.6	27.0	26.9	27.8	27.6	26.6	27.3	27.3	27.1	26.6	26.0	24.9	24.8	26.2	27.0	26.1	0.0	0.9	0.04

AAMP	27.4	27.7	27.7	27.4	27.3	27.2	27.3	27.4	27.4	27.4	27.0	27.6	26.9	26.7	26.7	26.5	26.4	26.1	26.8	26.0	27.4	26.4	0.0	0.9	0.04
CHMP2B	25.7	24.8	24.6	25.1	25.6	24.4	26.0	25.7	25.8	25.7	25.4	24.5	25.3	25.9	25.1	24.9	24.7	22.8	24.4	24.5	25.5	24.6	0.0	0.9	0.04
COL4A3BP	27.2	27.4	27.2	26.8	27.2	27.6	27.4	27.4	27.2	27.0	27.3	26.6	27.1	27.3	26.7	26.3	26.5	26.2	26.3	25.9	27.2	26.3	0.0	0.9	0.04
UTP20	28.4	28.8	28.6	28.9	28.7	28.8	28.3	28.8	28.5	28.6	28.5	29.3	28.1	28.3	28.3	26.2	27.7	27.7	28.0	27.5	28.6	27.7	0.0	0.9	0.04
HSPA14	28.2	28.2	28.3	27.7	28.2	28.2	28.1	28.3	28.0	28.3	27.9	27.8	28.0	27.9	28.0	26.0	27.2	27.2	27.9	27.1	28.1	27.2	0.0	0.9	0.04
SET	33.0	32.8	33.0	32.8	32.9	32.9	33.0	32.7	33.1	32.7	32.9	32.4	33.0	33.0	32.8	31.7	32.1	32.2	31.9	31.8	32.9	32.0	0.0	0.9	0.04
PDPR	27.5	27.1	26.8	27.1	26.9	26.7	26.9	27.2	26.9	26.9	26.6	27.4	26.8	27.3	26.7	25.2	25.8	25.8	26.2	26.7	26.9	26.0	0.0	0.9	0.04
SLC7A1	26.0	26.2	26.4	27.2	26.9	26.6	25.8	26.4	26.5	26.3	26.2	26.7	26.1	26.3	25.7	24.7	25.2	25.7	25.9	23.9	26.3	25.4	0.0	0.9	0.04
RCC2	32.4	32.4	32.5	32.6	32.6	32.9	32.7	32.5	32.5	32.4	32.4	32.3	32.5	32.4	32.2	30.7	31.7	31.6	32.0	31.4	32.5	31.6	0.0	0.9	0.04
NLN	30.4	30.3	30.1	30.4	30.1	30.3	30.4	30.4	30.1	30.1	30.1	30.1	30.2	30.3	29.4	28.9	29.3	29.3	29.4	29.0	30.2	29.3	0.0	0.9	0.04
LRRC1	24.3	25.4	23.8	23.7	24.2	NaN	24.2	23.5	24.3	24.0	24.1	24.0	23.3	23.3	22.9	22.4	23.2	23.0	23.7	23.2	24.0	23.1	0.0	0.9	0.04
PHC1	25.3	24.8	25.7	26.0	25.7	25.6	24.9	25.3	25.5	26.2	25.3	24.4	24.1	25.1	25.3	24.5	24.0	24.4	24.2	NaN	25.3	24.4	0.0	0.9	0.04
CYB5A	28.6	28.4	27.2	28.2	28.7	28.0	28.6	28.5	28.5	28.3	28.1	28.6	28.2	28.6	27.6	27.9	27.5	27.2	27.0	27.6	28.4	27.5	0.0	0.9	0.04
TIMM8A	26.5	25.9	25.4	26.7	27.2	27.6	26.5	27.0	26.6	26.6	26.1	27.1	25.3	26.6	26.3	25.5	25.9	25.5	26.0	25.5	26.6	25.7	0.0	0.9	0.04
MAD2L2	23.2	24.6	24.1	23.8	25.5	26.7	23.7	24.4	24.4	23.6	24.9	23.8	21.8	24.6	22.3	23.3	23.9	23.3	23.4	NaN	24.2	23.3	0.0	0.9	0.04
DPYSL3	33.0	33.3	33.5	33.0	33.2	33.6	33.1	33.3	33.3	33.1	33.4	33.1	33.1	33.2	32.8	33.1	32.4	32.2	32.2	31.7	33.2	32.3	0.0	0.9	0.04
MAP3K4	25.2	25.6	25.2	24.9	24.4	24.8	25.0	26.3	24.5	24.2	25.2	25.3	24.9	24.7	24.4	24.2	25.1	23.9	23.9	23.5	25.0	24.1	0.0	0.9	0.04
NCBP2-AS2	23.2	24.2	23.0	22.8	22.6	23.1	23.3	24.4	23.5	23.3	23.9	22.5	22.8	23.5	22.8	22.2	21.9	22.4	21.9	23.0	23.2	22.3	0.0	0.9	0.04
ZNF532	27.0	27.2	26.9	27.0	26.9	26.9	26.6	26.7	27.0	27.3	27.2	26.9	27.0	27.0	26.7	25.4	26.3	26.3	25.8	25.9	27.0	26.1	0.0	0.9	0.04
MARS	31.3	31.5	31.2	31.0	31.0	31.7	31.2	31.6	31.3	31.2	31.3	30.8	31.4	31.3	30.8	30.1	30.9	30.3	30.4	30.4	31.3	30.4	0.0	0.9	0.04
GAN	24.5	24.0	23.8	24.1	24.6	24.8	24.4	23.8	24.5	24.1	24.0	23.7	24.1	23.9	24.2	23.3	22.7	23.6	23.0	22.6	24.1	23.2	0.0	0.9	0.04
HLTF	26.2	26.5	25.3	25.9	26.2	25.8	25.6	26.4	25.6	25.4	26.1	25.1	26.5	26.5	25.2	24.7	24.9	25.0	25.7	25.8	26.0	25.1	0.0	0.9	0.05
USP9X	30.4	30.2	30.3	30.4	29.7	29.7	31.1	30.8	29.9	30.6	30.0	30.1	30.4	29.7	29.2	29.5	28.9	30.4	29.5	29.1	30.2	29.4	0.0	0.9	0.05
MCU	27.0	28.0	25.8	27.1	26.6	26.9	26.7	27.6	26.4	26.5	26.7	27.5	26.4	27.2	26.0	26.2	25.8	25.2	26.0	25.7	26.8	25.9	0.0	0.9	0.05
TBRG4	29.1	29.4	29.1	29.1	28.5	28.7	29.3	29.4	28.9	29.1	29.2	30.0	29.1	28.9	29.1	27.1	28.5	27.9	29.1	28.0	29.1	28.3	0.0	0.9	0.05
WDHD1	29.3	29.1	29.1	29.1	29.1	29.4	29.8	29.3	29.3	29.2	29.3	28.7	29.1	29.4	28.9	27.3	28.3	28.5	28.6	27.5	29.3	28.4	0.0	0.9	0.05
NOP16	27.9	27.5	27.6	28.0	27.8	27.6	27.5	27.7	27.7	28.0	27.6	27.8	26.9	27.7	27.5	26.0	26.6	26.6	27.3	27.0	27.7	26.8	0.0	0.9	0.05
CCBL1	26.0	25.6	25.0	24.9	25.9	26.0	25.4	26.4	25.8	25.3	25.7	24.5	26.7	26.2	25.5	25.1	24.3	25.1	24.7	24.4	25.8	24.9	0.0	0.9	0.05
TRMT1L	27.0	26.6	27.0	26.8	27.0	26.9	27.1	27.0	27.0	27.1	27.0	26.7	27.0	26.7	26.9	24.9	26.3	26.2	25.6	26.0	27.0	26.1	0.0	0.9	0.05
DAZAP1	30.3	29.8	29.5	29.6	30.1	30.4	30.1	30.3	30.4	30.0	29.9	29.9	30.0	30.3	29.7	29.0	29.3	28.4	29.1	29.3	30.0	29.2	0.0	0.9	0.05
ORC2	25.8	27.1	26.2	26.3	25.9	26.4	25.6	27.0	25.9	26.3	26.2	25.8	26.2	26.3	25.7	24.7	25.5	NaN	24.8	25.3	26.2	25.3	0.0	0.9	0.05
GEMIN7	26.1	25.7	25.9	26.0	25.5	26.4	25.5	24.8	25.6	25.3	25.8	25.1	25.4	25.0	25.6	NaN	24.7	25.2	24.7	24.4	25.5	24.7	0.0	0.9	0.05
RANGRF	23.4	24.7	23.1	23.7	23.2	23.7	24.4	24.2	24.1	23.4	23.6	22.5	23.7	23.4	23.2	22.3	23.2	23.0	21.8	22.5	23.6	22.8	0.0	0.9	0.05
