# Supplementary material for: Reprogrammed Cells Display Distinct Proteomic Signatures Associated with Colony Morphology Variability
Source: Stem Cells Int. 2019 Nov 18;2019:8036035. doi: 10.1155/2019/8036035 (PMC6885794; doi:10.1155/2019/8036035)
Supplement: Supplementary 1 — Supplementary table 1: information about the hiPSC lines included in the study. Supplementary table 2: a list of proteins being more abundant in stable colony morphology group. Supplementary table 3: a list of proteins being more abundant in the unstable colony morphology group. Supplementary table 4: activated upstream regulators in the unstable colony morphology group. [file 8036035.f1.zip › Supplementary table 3.pdf]

Supplement table 3: Proteins being more abundant (named by gene names) in unstable colony morphology group.
S=stable colony morphology group,U-1= Unstable class 1 morphology group, U-2=unstable class 2 morphology group .
FC=fold change. The list is sorted by FC. All proteins have a p-value <0.05 and a FC p-value <0.05. The list includes 338 proteins.
Expression values are log2 transformed LFQ intensity values.

Gene names	1-C S	3-A S	4-B S	5-A S	5-B S	6-B S	7-A S	7-B S	3-D S	1-A S	1-B S	2-A S	2-B S	2-C S	3-B U-1	6-A U-1	6-C U-1	4-C U-1	3-C U-2	4-A U-2	Median S	Median U	p-value	FC S/U	FC p-value
MRC2	19.8	21.3	21.4	20.3	NaN	21.3	20.5	NaN	20.9	21.9	20.6	21.9	22.7	22.4	23.0	31.2	28.9	28.0	26.0	29.1	21.3	28.5	0.0	-7.2	0
FN1	21.0	22.3	21.3	21.6	20.9	21.0	21.2	NaN	20.8	21.2	21.0	20.8	21.1	21.2	21.4	29.1	28.0	22.1	27.6	28.7	21.1	27.8	0.0	-6.7	0
MAP2	21.7	23.7	23.8	22.7	22.5	NaN	21.2	22.0	22.9	26.9	28.5	22.8	27.0	26.9	29.1	31.9	29.0	31.3	27.2	23.3	22.9	29.0	0.0	-6.2	0
FKBP9	NaN	19.5	NaN	20.8	NaN	NaN	20.1	19.2	NaN	21.7	21.2	NaN	22.4	21.5	23.2	27.8	26.4	26.5	27.1	30.3	21.0	26.8	0.0	-5.9	0
EPB41L3	22.0	21.3	22.3	20.3	22.5	23.3	22.3	21.7	22.2	22.2	22.2	22.0	23.8	22.1	24.8	30.0	25.2	27.2	28.7	28.9	22.2	27.9	0.0	-5.8	0
ZC3HAV1L	NaN	20.2	20.9	21.6	NaN	NaN	NaN	22.1	NaN	20.9	23.3	22.7	23.5	22.4	25.9	25.4	29.1	27.5	27.8	28.9	22.1	27.7	0.0	-5.6	0
SMURF1	19.7	NaN	20.3	19.7	NaN	NaN	19.6	NaN	20.6	19.9	19.9	20.9	23.4	19.7	24.7	24.9	25.4	24.9	25.8	26.1	19.9	25.2	0.0	-5.3	0
LGALS1	26.1	26.4	25.4	23.6	22.7	24.2	25.2	26.2	25.4	26.2	25.2	26.5	28.0	26.5	29.0	31.8	30.9	30.0	31.2	31.0	25.8	31.0	0.0	-5.2	0
ANXA1	27.5	26.5	23.1	28.1	27.2	24.8	26.6	26.1	25.9	27.1	27.4	28.6	27.4	28.9	29.6	32.2	32.2	32.2	30.6	32.3	27.1	32.2	0.0	-5.1	0
NEK7	21.8	22.0	22.3	21.6	21.9	20.0	22.3	20.8	21.3	22.4	22.2	22.1	23.5	21.8	25.6	26.7	27.1	26.8	27.9	28.6	22.0	27.0	0.0	-5.0	0
COL3A1	21.7	21.4	22.0	21.3	22.3	22.8	21.9	NaN	22.2	21.2	22.1	22.5	21.8	21.5	21.7	28.2	22.8	29.0	25.6	30.2	21.9	26.9	0.0	-5.0	0
PALLD	24.3	24.0	24.5	23.5	24.6	24.8	26.1	24.7	26.2	26.4	27.3	24.7	26.1	26.9	27.5	30.8	30.8	31.4	28.5	26.0	24.8	29.6	0.0	-4.8	0
NEFM	22.4	27.5	25.5	23.5	23.1	21.6	24.1	24.2	24.9	25.3	27.0	27.4	27.3	28.4	30.6	29.2	30.2	31.3	27.7	23.0	25.1	29.7	0.0	-4.6	1.6E-14
AHNAK	25.1	25.5	22.3	24.4	23.7	23.8	25.3	24.8	23.6	26.1	26.3	22.5	25.3	26.0	28.1	31.1	29.2	29.9	29.9	28.7	25.0	29.5	0.0	-4.6	2.5E-14
GSTM3	23.1	22.9	21.9	23.2	22.7	NaN	22.9	22.6	21.7	23.4	25.3	23.0	23.8	23.2	25.3	27.5	30.0	27.6	26.7	27.9	23.0	27.5	0.0	-4.6	3.0E-14
KCTD12	23.2	20.6	21.8	22.2	23.2	24.0	23.0	23.9	22.6	24.4	24.5	21.9	24.7	24.5	26.5	28.1	27.2	26.9	29.7	30.8	23.2	27.6	0.0	-4.4	1.4E-13
NEFL	NaN	24.3	19.6	NaN	NaN	NaN	20.6	NaN	20.7	24.8	25.6	22.8	24.5	26.5	27.7	29.4	29.1	31.9	26.0	26.6	24.3	28.4	0.0	-4.1	5.5E-12
CD99	23.4	22.6	NaN	NaN	21.8	NaN	22.3	22.1	22.6	22.5	23.9	22.3	24.8	23.5	25.4	27.0	26.3	27.1	26.0	27.9	22.6	26.7	0.0	-4.1	9.2E-12
CSRP1	25.0	22.5	NaN	22.9	23.9	24.6	25.4	24.4	22.6	25.0	25.2	23.8	26.3	26.1	25.6	28.5	28.7	26.6	29.1	29.2	24.6	28.6	0.0	-4.0	2.2E-11
ALDH1L2	20.7	NaN	22.4	NaN	NaN	NaN	NaN	19.8	21.6	21.6	21.1	20.7	20.3	NaN	22.3	24.5	25.3	21.3	25.5	29.8	20.9	24.9	0.0	-4.0	2.8E-11
CRIP2	23.6	24.2	21.8	NaN	NaN	NaN	NaN	NaN	NaN	26.3	26.8	25.1	26.2	26.2	28.0	31.0	29.9	29.6	27.6	29.5	25.6	29.6	0.0	-3.9	5.0E-11
MAP1A	22.2	22.4	25.0	22.8	21.1	22.3	24.3	20.4	21.5	23.6	23.9	22.0	23.7	23.4	23.5	29.0	27.2	27.5	23.1	25.8	22.6	26.5	0.0	-3.9	5.1E-11
PDLIM4	19.8	20.5	21.5	NaN	20.5	21.5	NaN	NaN	21.0	20.7	21.7	20.2	22.2	22.6	22.7	28.2	25.6	21.7	24.2	28.9	21.0	24.9	0.0	-3.8	1.1E-10
COL5A2	24.3	22.3	23.3	23.9	23.3	24.9	24.2	24.1	25.9	23.4	23.8	22.2	24.4	22.9	23.9	28.9	27.4	27.3	27.4	28.9	23.9	27.4	0.0	-3.5	2.9E-09
ABAT	21.7	NaN	23.6	21.6	22.0	NaN	22.0	NaN	23.4	24.5	24.8	21.6	23.7	24.6	25.0	29.7	27.5	26.9	21.9	26.8	23.4	26.8	0.0	-3.4	8.7E-09
GPX7	23.5	21.5	23.0	25.1	23.7	23.9	23.8	22.3	23.3	23.7	24.5	22.4	24.7	24.1	25.1	27.1	29.2	27.0	27.0	29.9	23.7	27.1	0.0	-3.4	9.1E-09
CAPN2	21.3	24.1	25.9	25.0	22.9	25.1	24.5	25.7	24.4	25.7	25.6	25.9	26.5	25.0	26.7	30.4	28.2	29.3	28.7	27.9	25.1	28.4	0.0	-3.4	1.4E-08
CAP2	25.2	26.2	25.8	25.8	24.7	25.8	26.3	24.8	25.9	26.2	26.4	25.4	26.8	26.3	27.6	30.5	29.4	29.5	28.6	28.9	25.8	29.2	0.0	-3.4	1.5E-08
GSTM2	24.9	23.4	24.4	24.6	22.5	23.7	24.4	25.3	24.0	25.2	25.0	24.2	25.8	24.8	26.4	29.2	28.4	27.0	27.3	28.4	24.5	27.9	0.0	-3.4	1.6E-08
NCAM1	NaN	23.7	24.0	NaN	22.5	NaN	NaN	NaN	NaN	23.6	25.5	24.2	24.9	24.4	26.0	29.1	27.5	26.7	27.3	27.9	24.1	27.4	0.0	-3.3	2.1E-08
PBK	23.9	23.4	24.2	23.0	NaN	22.6	21.9	23.3	NaN	24.2	24.2	23.2	23.6	24.1	25.9	26.9	27.3	27.3	26.8	25.9	23.5	26.8	0.0	-3.3	3.0E-08
EPM2AIP1	22.0	21.7	NaN	NaN	NaN	22.4	21.1	21.4	22.3	22.4	23.2	22.2	23.3	21.2	23.8	27.2	24.6	24.8	25.9	28.2	22.2	25.3	0.0	-3.2	9.5E-08
DPYSL5	26.7	28.5	26.4	24.1	25.3	24.7	26.6	25.8	27.3	29.3	30.0	27.1	29.0	29.5	29.8	32.4	30.2	32.1	28.6	28.4	26.9	30.0	0.0	-3.1	1.2E-07

STX12	23.0	22.6	22.8	NaN	23.1	NaN	NaN	22.4	23.5	23.8	23.6	23.3	22.0	23.6	24.1	27.9	23.4	27.1	26.4	26.0	23.1	26.2	0.0	-3.1	1.8E-07
CELF2	24.5	23.9	25.3	25.7	23.8	24.2	24.9	23.7	25.0	25.3	24.9	26.5	24.9	23.7	25.6	27.8	24.5	28.5	28.1	28.7	24.9	27.9	0.0	-3.1	2.0E-07
RHOB	21.9	NaN	NaN	22.9	22.6	NaN	NaN	22.4	NaN	22.7	22.8	22.1	22.9	23.0	25.1	27.4	23.9	27.3	26.1	25.4	22.7	25.7	0.0	-3.0	3.1E-07
COL4A2	22.0	NaN	22.1	23.0	22.7	21.9	22.6	21.2	23.2	23.2	23.1	23.0	23.1	22.3	24.5	26.0	25.4	26.9	24.3	27.0	22.7	25.7	0.0	-3.0	3.2E-07
ARHGAP18	NaN	NaN	21.2	20.5	20.8	NaN	21.8	NaN	20.9	21.1	20.7	NaN	22.2	20.6	22.9	22.5	21.8	24.8	26.0	26.7	20.9	23.9	0.0	-2.9	5.4E-07
FAM114A1	21.0	24.5	NaN	20.8	21.3	21.2	22.2	22.2	21.5	21.4	21.9	19.9	21.4	21.6	21.1	27.6	27.0	23.4	22.1	25.2	21.4	24.3	0.0	-2.9	7.2E-07
ANXA2	31.5	31.1	30.8	32.2	31.4	31.2	32.4	31.6	31.2	30.7	31.1	31.4	31.6	32.4	31.8	34.7	34.0	34.9	33.9	34.6	31.4	34.3	0.0	-2.9	8.0E-07
PLOD2	25.8	24.5	21.7	22.4	25.7	24.4	25.7	23.9	24.8	23.5	25.5	23.8	25.2	25.2	23.8	28.3	30.6	25.0	26.8	29.5	24.7	27.5	0.0	-2.9	1.2E-06
CD44	21.9	22.1	NaN	21.9	22.0	22.2	NaN	NaN	21.5	21.7	21.9	NaN	NaN	23.5	24.0	26.7	27.5	24.3	24.1	25.1	21.9	24.7	0.0	-2.7	2.9E-06
SUN2	24.7	24.4	23.1	22.8	23.5	NaN	23.3	23.6	23.7	25.8	24.7	25.0	24.6	25.6	26.1	27.7	26.9	25.3	27.3	28.0	24.4	27.1	0.0	-2.7	3.9E-06
ME1	24.6	27.3	26.8	27.0	23.4	NaN	26.5	26.0	24.5	24.0	26.4	25.9	24.6	25.3	26.3	28.0	29.3	29.5	26.3	29.4	25.9	28.6	0.0	-2.7	4.7E-06
ITGA11	NaN	22.3	22.7	22.9	NaN	NaN	22.2	NaN	22.3	21.8	22.6	22.2	22.8	23.0	23.9	27.6	26.4	28.0	23.8	23.9	22.5	25.1	0.0	-2.6	5.8E-06
ACSS2	24.9	25.8	27.3	27.6	25.3	26.0	25.8	25.7	25.5	25.2	26.7	25.1	26.8	26.8	26.9	28.1	28.6	29.6	28.6	28.3	25.8	28.4	0.0	-2.6	6.3E-06
FNDC3A	NaN	NaN	24.1	24.1	NaN	21.8	NaN	22.2	22.2	22.5	NaN	22.3	NaN	23.8	22.7	23.5	26.5	27.4	23.5	26.8	22.4	25.0	0.0	-2.6	7.1E-06
FLNC	27.4	26.9	25.2	28.8	28.1	25.2	27.6	27.3	26.4	27.2	27.1	28.9	27.8	28.9	29.4	31.1	29.4	31.0	28.7	30.6	27.4	30.0	0.0	-2.6	7.2E-06
COMMD2	21.5	NaN	22.1	21.7	22.1	22.0	22.2	21.9	21.4	21.6	23.3	21.9	22.0	21.8	23.6	25.1	25.3	23.1	24.0	25.2	21.9	24.5	0.0	-2.6	9.6E-06
CFL2	24.5	25.0	25.7	25.3	23.8	23.9	25.1	25.2	25.1	25.8	26.0	25.8	25.8	25.0	26.3	28.0	27.4	28.4	28.0	27.1	25.1	27.7	0.0	-2.6	9.9E-06
ITGA5	23.8	NaN	23.6	23.4	23.5	24.0	23.5	23.4	NaN	24.2	24.3	23.2	24.8	23.1	25.1	27.1	27.6	27.3	24.9	25.1	23.6	26.1	0.0	-2.6	1.1E-05
SNX18	23.0	21.4	21.4	23.0	23.0	NaN	21.7	22.3	22.0	23.0	23.2	22.0	22.6	23.0	23.2	24.8	27.3	26.2	23.1	25.6	22.6	25.2	0.0	-2.6	1.2E-05
RELA	23.8	NaN	23.3	23.2	23.8	NaN	23.3	23.2	23.0	23.8	23.6	23.4	23.7	23.7	24.3	26.3	25.9	26.1	26.4	26.0	23.5	26.1	0.0	-2.5	1.4E-05
HPCAL1	NaN	NaN	21.0	20.5	NaN	NaN	NaN	20.9	NaN	21.4	22.3	20.5	23.0	21.5	23.9	25.8	21.8	23.6	23.4	26.4	21.2	23.7	0.0	-2.5	1.5E-05
CADM1	24.5	24.7	24.1	25.6	25.5	23.9	24.5	24.4	25.0	24.5	24.7	24.7	24.9	24.7	25.5	27.5	27.2	27.9	26.8	NaN	24.7	27.2	0.0	-2.5	1.5E-05
IKBIP	23.7	24.7	23.7	23.9	24.0	24.0	24.4	23.4	23.1	23.8	23.4	24.5	23.6	23.7	25.2	27.1	25.9	NaN	26.3	27.7	23.8	26.3	0.0	-2.5	1.6E-05
VIM	31.5	33.9	33.7	33.7	33.5	31.7	33.2	33.4	33.7	33.3	34.0	34.0	34.4	34.8	35.5	37.6	36.6	36.8	35.7	35.7	33.7	36.2	0.0	-2.5	1.7E-05
KDELC2	24.9	24.4	24.6	25.3	24.0	NaN	25.1	25.0	24.5	25.0	25.1	24.1	25.9	24.8	26.7	27.3	27.6	27.1	27.4	29.0	24.9	27.4	0.0	-2.5	1.9E-05
PARVA	25.3	26.5	25.0	26.2	25.0	24.5	25.9	26.1	25.3	25.3	26.3	25.4	26.4	26.8	26.7	28.3	28.5	28.7	27.5	27.9	25.6	28.1	0.0	-2.5	2.0E-05
CAMK2D	26.1	27.3	27.1	25.1	26.4	26.0	26.2	26.4	26.3	26.8	27.4	26.8	27.8	27.5	28.4	30.7	28.7	30.3	29.2	29.0	26.6	29.1	0.0	-2.5	2.3E-05
LRRC57	21.9	23.5	22.5	22.8	NaN	NaN	22.7	23.2	22.2	22.4	22.3	22.3	22.2	22.4	24.2	24.8	24.4	25.4	24.8	24.9	22.4	24.8	0.0	-2.5	2.3E-05
GNG12	24.8	24.9	24.0	25.3	25.0	25.3	25.4	24.7	24.8	24.6	24.7	25.0	25.5	25.2	25.5	26.9	27.1	27.8	27.9	27.9	25.0	27.4	0.0	-2.5	2.3E-05
PTGFRN	20.9	23.1	22.8	20.9	21.1	NaN	NaN	NaN	NaN	21.5	21.5	20.5	23.1	20.8	22.8	24.6	25.9	22.9	22.6	29.2	21.3	23.8	0.0	-2.4	2.5E-05
AFAP1	21.0	21.1	NaN	NaN	21.3	NaN	NaN	20.3	20.9	21.6	NaN	21.2	22.9	21.3	23.0	24.1	23.4	24.7	23.9	22.0	21.2	23.7	0.0	-2.4	2.8E-05
RANBP6	NaN	22.5	21.8	23.6	22.3	23.0	22.5	20.7	NaN	22.4	22.7	22.6	22.5	NaN	23.4	25.2	25.3	25.0	24.8	24.8	22.5	24.9	0.0	-2.4	3.0E-05
PTGR1	24.6	24.2	25.8	25.5	26.0	24.8	25.9	25.7	24.6	25.1	24.6	24.4	27.1	24.7	24.9	27.2	27.7	28.0	26.9	27.6	25.0	27.4	0.0	-2.4	3.3E-05
HSPA4L	23.2	22.6	23.5	23.5	24.9	22.7	23.6	NaN	22.8	24.0	23.7	NaN	23.0	22.9	23.5	25.3	26.7	25.8	NaN	25.8	23.4	25.8	0.0	-2.4	3.6E-05
LNP	NaN	NaN	21.7	22.2	21.5	NaN	21.9	NaN	22.3	23.0	NaN	25.9	22.8	22.5	23.9	25.1	24.8	24.9	23.5	24.5	22.3	24.6	0.0	-2.4	3.9E-05
ARSA	22.5	22.6	23.1	22.4	24.1	25.0	22.7	NaN	22.8	22.3	22.3	22.9	22.0	22.3	23.1	26.3	25.2	24.0	24.6	26.4	22.6	24.9	0.0	-2.3	6.0E-05
LMF2	24.1	23.2	23.9	23.0	24.5	23.3	24.0	22.2	23.9	23.7	24.8	23.3	24.2	23.8	24.2	27.8	26.1	26.2	23.9	26.3	23.8	26.2	0.0	-2.3	6.1E-05
CDH11	21.9	22.8	NaN	22.3	NaN	21.7	21.4	21.4	21.9	23.3	22.0	21.8	23.0	22.9	22.9	23.7	24.8	23.6	24.8	27.9	22.0	24.2	0.0	-2.3	9.3E-05
VPS13C	22.2	NaN	NaN	23.1	23.0	NaN	24.3	NaN	23.8	23.3	22.6	NaN	23.0	NaN	23.3	25.8	25.0	24.9	25.7	27.3	23.1	25.3	0.0	-2.3	9.4E-05
H2AFY2	26.9	28.4	27.6	26.3	26.2	26.1	26.4	26.9	26.6	28.5	28.9	27.7	28.5	27.8	29.5	29.4	29.7	29.5	29.0	29.6	27.3	29.5	0.0	-2.2	1.0E-04
B3GALTL	NaN	NaN	23.9	24.0	23.6	NaN	22.4	NaN	23.4	23.5	23.4	NaN	22.8	23.0	25.7	25.2	24.0	26.9	NaN	26.0	23.4	25.7	0.0	-2.2	1.1E-04
RAB31	25.2	23.8	23.0	23.9	23.9	24.3	23.6	24.3	23.6	25.5	25.4	24.8	24.7	24.3	25.4	28.5	23.9	27.2	26.5	26.4	24.3	26.5	0.0	-2.2	1.4E-04

CNPY4	23.8	25.3	23.7	23.8	24.1	24.3	23.9	24.6	23.8	24.0	23.9	NaN	23.4	23.7	24.2	26.6	26.1	NaN	NaN	26.0	23.9	26.1	0.0	-2.2	1.4E-04
TSPAN3	20.9	23.2	24.6	24.5	NaN	NaN	20.9	NaN	NaN	24.4	24.6	24.1	23.6	21.9	24.9	26.6	26.0	26.3	NaN	25.4	23.8	26.0	0.0	-2.2	1.5E-04
FNDC3B	23.2	23.4	23.8	25.2	22.7	NaN	23.9	24.5	23.5	23.4	23.7	24.0	22.7	24.5	23.9	25.7	26.3	26.0	24.6	27.1	23.7	25.8	0.0	-2.2	1.5E-04
RND3	20.9	21.1	21.3	NaN	21.2	21.8	NaN	21.1	NaN	21.0	NaN	21.9	22.2	NaN	22.7	23.0	21.7	24.2	23.8	23.6	21.2	23.3	0.0	-2.2	1.6E-04
GSTK1	25.1	23.5	25.7	24.4	24.6	23.9	23.4	25.1	25.4	25.6	25.5	26.2	25.9	25.5	25.4	28.2	27.7	27.1	27.3	27.6	25.3	27.4	0.0	-2.2	1.7E-04
FAM120A	25.8	26.3	25.8	26.4	26.2	26.0	26.6	26.2	26.0	26.1	26.1	25.2	26.0	25.7	26.5	28.1	28.4	27.7	28.3	28.9	26.0	28.2	0.0	-2.2	1.7E-04
ALCAM	26.5	26.9	27.9	27.7	26.3	25.7	26.4	26.4	26.9	26.6	26.6	26.9	26.7	26.9	27.5	28.7	31.0	28.7	29.0	30.8	26.7	28.8	0.0	-2.1	1.9E-04
ZNF428	24.8	25.3	25.5	25.0	25.1	25.3	24.8	24.7	25.2	25.8	25.9	25.1	24.8	25.3	27.1	28.2	27.3	27.3	26.8	27.5	25.1	27.3	0.0	-2.1	2.2E-04
MARCKS	27.2	27.3	28.6	27.8	27.2	26.9	27.6	26.9	27.1	28.4	28.4	27.7	28.7	28.2	29.0	30.3	29.5	31.5	29.8	29.8	27.7	29.8	0.0	-2.1	2.2E-04
ACOT1	26.1	26.0	25.2	26.8	25.6	25.6	25.7	25.7	25.3	25.0	25.6	25.4	26.2	25.9	26.5	27.0	28.1	27.5	28.3	29.4	25.6	27.8	0.0	-2.1	2.2E-04
NIPSNAP3A	24.4	NaN	23.9	24.2	NaN	23.4	24.7	24.0	24.3	24.8	25.1	24.3	25.6	24.4	25.1	27.7	26.6	26.3	26.1	28.3	24.3	26.5	0.0	-2.1	2.3E-04
CD63	25.7	24.5	25.9	26.2	23.3	22.8	25.0	24.4	24.8	26.3	24.5	26.5	25.4	24.8	25.8	27.7	26.7	27.9	26.8	27.3	24.9	27.0	0.0	-2.1	2.4E-04
CA2	25.0	24.1	23.7	24.1	23.3	24.8	24.3	NaN	23.6	24.5	24.5	NaN	23.5	25.4	24.5	27.9	26.7	25.1	26.4	26.2	24.2	26.3	0.0	-2.1	2.4E-04
AKT1	23.5	24.6	23.5	24.8	23.6	24.7	23.6	25.1	23.1	23.9	24.0	24.2	24.3	23.2	24.5	25.7	25.9	26.0	26.0	27.4	23.9	26.0	0.0	-2.1	2.9E-04
SDC2	23.8	22.7	23.1	24.4	23.2	24.3	23.3	NaN	23.6	25.2	23.9	23.9	24.3	24.0	23.9	26.2	25.8	27.8	26.1	25.3	23.9	26.0	0.0	-2.1	3.0E-04
CAT	26.1	25.7	25.8	26.7	26.3	26.9	26.0	26.4	26.0	25.9	26.1	26.0	26.4	25.9	26.7	28.2	28.7	27.2	27.9	28.9	26.0	28.1	0.0	-2.0	3.7E-04
CAST	23.7	23.1	22.7	22.7	20.8	22.4	25.1	24.4	23.1	23.7	23.7	22.7	23.1	23.7	24.4	25.0	28.2	24.6	25.3	28.2	23.1	25.1	0.0	-2.0	3.8E-04
ANK2	25.2	23.2	25.5	25.5	24.7	22.2	25.5	26.1	25.3	25.8	25.3	26.2	26.4	23.9	26.7	29.7	25.5	26.8	29.4	28.0	25.4	27.4	0.0	-2.0	3.9E-04
ANXA6	30.5	30.7	30.9	30.6	30.3	30.5	31.0	31.1	30.5	30.8	30.9	31.0	31.6	31.0	31.5	33.4	33.0	32.7	32.2	33.5	30.8	32.9	0.0	-2.0	4.4E-04
FARP1	26.2	26.5	26.6	25.0	26.3	26.7	25.0	26.3	23.8	25.3	26.1	26.8	26.4	26.5	27.1	28.7	27.9	27.8	28.7	30.0	26.3	28.3	0.0	-2.0	4.8E-04
LMNA	29.4	29.3	28.9	29.8	29.2	29.5	29.7	29.3	29.2	29.4	29.2	29.6	29.0	29.6	30.1	32.1	31.2	31.4	31.3	32.6	29.4	31.4	0.0	-2.0	4.9E-04
DOCK7	25.3	25.7	25.7	25.9	25.5	25.6	24.5	26.2	25.5	25.7	26.0	25.9	27.1	25.9	26.9	27.8	28.2	28.2	26.9	27.5	25.7	27.7	0.0	-2.0	4.9E-04
CDK6	24.7	24.6	24.6	24.9	23.6	22.3	24.3	24.8	24.5	25.9	26.1	26.0	26.1	26.4	26.4	29.0	26.6	28.5	26.8	24.9	24.8	26.7	0.0	-2.0	5.1E-04
CBFB	23.4	24.5	23.4	23.4	NaN	NaN	23.8	24.1	23.5	25.2	24.0	24.2	23.9	23.4	25.6	24.3	26.1	25.2	26.1	26.2	23.8	25.8	0.0	-2.0	5.1E-04
PON2	25.2	24.2	25.4	25.8	24.6	23.4	25.4	25.5	25.1	25.8	26.7	26.2	26.9	26.4	26.9	31.2	27.3	28.1	25.7	27.4	25.4	27.4	0.0	-2.0	5.6E-04
MVK	25.8	26.4	26.0	26.1	26.2	26.2	24.9	26.5	26.0	25.0	26.1	26.0	25.8	26.4	26.7	28.6	28.0	27.9	28.1	28.0	26.0	28.0	0.0	-1.9	6.2E-04
ACSL1	24.8	24.7	25.3	23.5	22.3	22.7	24.8	24.2	24.3	23.4	22.8	25.3	24.2	24.3	24.8	25.8	26.5	23.7	26.9	28.7	24.2	26.2	0.0	-1.9	6.3E-04
TCAF1	24.6	23.6	25.2	23.5	24.3	23.3	24.8	23.2	24.5	24.7	24.9	24.8	24.9	24.2	25.1	26.1	27.2	27.1	26.8	25.9	24.5	26.4	0.0	-1.9	6.8E-04
HP1BP3	27.5	28.1	27.7	27.6	28.1	28.6	27.6	27.8	27.9	28.0	28.6	28.3	28.5	28.2	29.4	30.8	30.3	29.6	29.6	30.6	28.0	30.0	0.0	-1.9	7.0E-04
RP2	23.3	23.3	23.2	24.2	23.6	22.3	23.5	20.5	23.6	23.2	23.8	23.5	23.3	23.6	23.0	27.5	25.7	26.9	25.0	23.9	23.4	25.3	0.0	-1.9	7.3E-04
CLN5	22.6	NaN	22.9	24.3	22.0	NaN	22.3	NaN	21.7	22.6	22.4	NaN	22.5	22.5	22.8	26.0	22.9	26.2	22.9	26.8	22.5	24.4	0.0	-1.9	7.6E-04
LDLR	23.7	23.5	24.0	23.9	24.2	24.1	23.7	NaN	24.3	24.8	24.7	24.6	23.6	23.3	25.5	26.2	26.0	27.1	25.9	23.1	24.0	26.0	0.0	-1.9	7.6E-04
COMMD8	23.9	25.2	23.5	23.8	24.5	25.0	23.6	24.2	24.7	24.9	24.5	24.6	24.1	23.9	25.0	26.3	26.1	26.4	26.5	25.9	24.3	26.2	0.0	-1.9	7.8E-04
HMGN2	26.2	24.5	28.0	27.5	25.9	26.3	26.4	24.4	26.1	27.7	28.1	26.1	27.9	26.7	27.8	28.3	28.7	28.8	27.8	28.2	26.4	28.3	0.0	-1.9	7.9E-04
LAMB1	26.0	27.1	27.5	28.8	25.2	25.5	26.9	26.9	26.4	27.5	27.6	29.0	27.2	27.3	28.5	29.1	29.8	29.0	28.5	29.2	27.1	29.1	0.0	-1.9	7.9E-04
ATL3	25.5	25.1	24.6	26.1	24.0	26.2	24.9	25.1	24.4	24.9	23.4	25.9	25.3	24.5	25.0	26.8	27.0	26.3	27.4	28.9	25.0	26.9	0.0	-1.9	8.0E-04
SCARB2	26.4	26.3	26.4	27.0	26.1	25.3	26.9	26.3	26.8	27.4	26.0	27.5	27.2	27.1	27.5	30.3	27.8	28.9	28.1	30.1	26.6	28.5	0.0	-1.9	8.1E-04
MAP7D1	23.7	23.8	22.6	23.8	24.1	NaN	24.2	25.1	24.1	25.2	24.2	22.4	24.7	23.0	25.9	27.0	27.3	25.6	23.9	26.1	24.1	26.0	0.0	-1.9	8.5E-04
MEST	26.5	26.3	24.3	25.3	25.5	26.4	26.3	26.1	26.5	26.1	25.6	27.0	26.3	25.5	27.6	29.7	29.9	26.8	27.7	28.5	26.2	28.1	0.0	-1.9	8.6E-04
CEP170	25.7	25.5	25.4	24.9	25.7	26.0	25.3	24.4	25.5	26.5	26.5	24.2	25.7	26.3	27.2	28.1	27.8	26.5	27.2	27.9	25.6	27.5	0.0	-1.9	9.2E-04
SPARC	27.2	26.8	26.5	27.7	27.3	27.0	27.1	27.0	27.3	26.8	26.9	26.8	26.3	27.3	26.8	30.0	28.6	29.2	27.8	29.1	27.0	28.9	0.0	-1.9	9.2E-04
CRABP1	30.8	31.1	30.8	30.2	29.9	30.1	30.4	30.7	31.0	31.5	31.6	31.0	31.8	31.4	33.1	32.7	31.2	33.3	32.4	32.8	30.9	32.7	0.0	-1.8	1.3E-03

ACTR10	24.1	24.6	25.1	24.9	23.8	NaN	24.5	25.6	23.9	25.3	25.2	25.5	24.8	23.7	25.0	26.7	25.9	26.7	27.3	26.4	24.8	26.6	0.0	-1.8	1.3E-03
WHSC1	24.1	24.3	25.4	24.9	24.3	23.8	24.5	24.5	24.4	24.9	24.9	24.6	24.4	24.4	26.0	26.1	26.6	26.6	26.4	24.4	24.4	26.3	0.0	-1.8	1.3E-03
SMARCC2	26.5	26.8	26.7	26.3	26.0	25.7	26.7	27.2	26.7	26.7	27.2	27.0	27.0	26.9	27.5	29.0	28.4	28.6	28.0	28.6	26.7	28.5	0.0	-1.8	1.3E-03
HSPB1	28.4	28.7	27.6	27.6	27.7	28.5	28.6	28.6	28.5	28.6	28.5	27.9	28.1	28.5	29.3	31.6	30.5	30.0	29.8	31.6	28.5	30.3	0.0	-1.8	1.6E-03
TWF1	25.9	26.0	26.3	26.1	26.4	26.2	26.1	25.7	26.0	26.2	26.0	26.3	26.2	26.2	26.7	28.4	28.7	27.7	27.7	28.1	26.1	27.9	0.0	-1.8	1.6E-03
GNE	24.4	24.2	26.0	25.0	23.9	23.0	26.1	25.4	24.2	24.7	25.0	24.3	24.9	24.2	25.7	25.4	26.3	26.6	26.8	26.3	24.6	26.3	0.0	-1.8	1.7E-03
SCRN1	27.5	26.9	26.0	26.8	27.4	27.6	27.2	27.5	26.9	27.0	26.9	26.8	27.6	27.6	27.5	29.5	29.1	28.6	28.4	29.3	27.1	28.9	0.0	-1.8	1.7E-03
MMP14	25.1	25.5	25.0	26.1	25.8	25.1	25.7	25.7	25.0	25.4	25.6	25.3	25.8	25.5	25.0	27.8	27.4	27.1	26.0	27.5	25.5	27.2	0.0	-1.8	1.7E-03
ITGA2	NaN	NaN	24.5	22.8	22.5	22.0	24.5	NaN	24.0	24.1	22.9	21.6	24.7	24.2	24.3	28.0	26.2	23.8	NaN	25.8	24.0	25.8	0.0	-1.8	1.7E-03
DPY19L1	22.5	23.0	NaN	22.7	NaN	NaN	22.4	NaN	22.2	22.3	22.6	NaN	23.2	22.8	23.2	25.1	24.8	23.9	23.5	26.7	22.6	24.3	0.0	-1.8	1.8E-03
INA	NaN	24.1	NaN	NaN	22.4	NaN	23.8	24.1	23.6	23.0	25.6	25.9	23.6	25.3	25.4	27.0	NaN	24.4	25.7	26.9	23.9	25.7	0.0	-1.8	1.8E-03
GBA	25.5	25.7	25.5	26.4	24.9	26.5	25.5	25.5	25.4	25.0	25.1	26.1	25.3	25.8	25.7	28.7	27.4	27.2	27.2	28.9	25.5	27.3	0.0	-1.7	1.8E-03
TMX3	25.1	25.6	25.3	25.6	25.2	26.8	25.0	24.8	24.4	25.7	25.7	25.3	25.5	25.6	26.0	27.4	26.8	27.6	26.7	27.9	25.4	27.1	0.0	-1.7	1.8E-03
CRYZL1	24.6	24.5	25.8	25.6	23.9	24.2	25.5	24.5	24.6	25.4	25.5	25.4	25.9	23.9	26.2	27.1	26.8	27.4	26.6	26.7	25.0	26.7	0.0	-1.7	2.0E-03
TES	26.5	25.7	25.3	25.4	26.8	26.1	26.4	24.9	25.9	26.0	26.2	25.8	26.7	26.5	27.0	27.2	28.4	27.2	28.3	28.8	26.1	27.8	0.0	-1.7	2.2E-03
IDH2	30.0	30.2	29.2	29.7	29.8	29.7	29.9	30.0	29.6	30.0	30.4	30.0	30.9	30.2	31.5	31.8	31.4	31.5	32.5	33.4	30.0	31.7	0.0	-1.7	2.2E-03
PCYOX1L	24.3	24.7	24.1	23.4	23.5	23.1	23.6	22.9	23.9	25.2	24.8	25.9	25.8	25.8	25.5	25.8	24.5	26.0	26.5	27.4	24.2	25.9	0.0	-1.7	2.2E-03
CIRBP	28.4	27.7	27.6	27.7	28.9	29.3	27.9	28.1	28.2	28.0	28.7	27.7	27.8	28.0	29.5	30.2	30.1	28.7	28.6	30.0	28.0	29.7	0.0	-1.7	2.3E-03
SERPINB6	26.7	26.5	25.8	26.6	26.4	25.9	27.3	27.2	26.5	26.2	26.2	26.8	28.3	27.3	28.6	28.3	28.3	28.1	30.4	28.1	26.6	28.3	0.0	-1.7	2.4E-03
TTYH3	26.9	26.9	26.7	24.8	26.9	27.4	25.1	26.2	26.8	26.7	27.0	26.9	26.5	26.7	27.5	30.1	28.2	28.8	27.2	29.1	26.8	28.5	0.0	-1.7	2.4E-03
HAGH	24.5	23.8	24.5	24.1	23.7	24.2	24.5	24.5	24.5	24.3	24.5	24.1	24.7	24.3	24.8	27.0	25.3	26.5	26.0	26.2	24.4	26.1	0.0	-1.7	2.4E-03
PRAF2	25.0	25.9	25.3	25.9	25.1	24.5	25.2	24.1	25.3	25.8	25.7	25.2	25.5	25.7	26.2	27.7	27.1	27.2	26.6	26.8	25.3	27.0	0.0	-1.7	2.4E-03
PANK4	24.7	25.1	24.5	23.8	24.4	24.8	25.3	25.3	24.0	24.3	24.5	23.8	26.0	26.5	26.0	26.5	26.2	25.5	26.6	26.6	24.6	26.3	0.0	-1.7	2.5E-03
PDLIM7	27.6	27.8	28.3	27.5	28.0	27.9	27.3	27.6	27.6	28.4	28.1	27.3	27.1	27.5	28.1	29.8	29.6	29.8	28.8	28.9	27.6	29.3	0.0	-1.7	2.5E-03
NNT	27.4	27.8	27.4	28.0	27.5	26.9	27.5	27.3	27.8	28.1	28.0	28.6	27.9	27.7	29.0	29.5	29.4	29.5	30.1	30.5	27.8	29.5	0.0	-1.7	2.7E-03
COMMD4	22.1	NaN	22.3	21.4	NaN	NaN	22.3	NaN	22.2	22.2	23.0	NaN	22.3	22.3	23.3	24.0	24.5	23.2	NaN	24.1	22.3	24.0	0.0	-1.7	2.7E-03
YIF1B	25.3	25.1	25.8	26.5	25.1	NaN	25.6	25.9	25.2	25.5	25.3	25.3	25.1	25.4	25.4	27.0	27.0	27.0	26.4	27.0	25.3	27.0	0.0	-1.7	2.7E-03
MMAB	25.2	25.0	25.6	25.4	25.1	24.8	24.4	24.2	24.8	25.5	25.4	25.5	25.6	25.3	26.5	27.3	26.6	27.0	26.7	27.1	25.2	26.9	0.0	-1.7	2.8E-03
CDH2	26.4	25.4	25.8	25.6	23.7	25.5	27.0	26.9	25.0	26.6	26.8	26.1	25.7	25.7	27.1	30.1	27.0	30.2	26.8	27.6	25.7	27.4	0.0	-1.6	3.1E-03
LZIC	24.6	NaN	25.6	23.9	NaN	24.3	24.3	24.5	24.8	25.6	25.4	24.1	25.8	24.6	26.0	26.9	26.5	26.0	25.7	26.9	24.6	26.3	0.0	-1.6	3.1E-03
GIT2	24.8	NaN	23.9	23.1	24.3	NaN	23.4	NaN	24.1	22.5	23.4	23.0	24.6	23.6	24.8	26.8	25.3	25.0	25.2	25.9	23.6	25.2	0.0	-1.6	3.2E-03
DYNC1LI2	27.0	27.9	26.4	26.2	26.5	27.0	26.5	26.7	27.0	27.8	27.2	27.3	27.3	27.0	27.5	28.8	28.6	28.7	28.1	28.7	27.0	28.6	0.0	-1.6	3.2E-03
IMPAD1	24.1	24.5	23.4	24.5	25.2	25.3	24.7	24.2	24.7	24.9	24.3	25.1	25.2	24.5	25.4	26.6	26.2	26.3	25.7	27.1	24.6	26.2	0.0	-1.6	3.3E-03
MAP1B	30.8	31.2	31.2	31.3	30.8	30.8	31.2	31.3	31.2	31.5	31.9	31.1	31.8	31.8	32.5	34.0	32.8	33.3	32.8	32.9	31.2	32.9	0.0	-1.6	3.3E-03
PLOD1	27.7	27.8	27.4	27.6	27.7	27.4	27.7	27.9	27.8	27.8	27.9	28.3	28.2	27.9	28.2	29.6	30.2	29.3	29.0	30.9	27.8	29.4	0.0	-1.6	3.7E-03
SEL1L	25.1	24.7	25.0	26.2	25.1	24.7	25.2	24.7	25.5	26.0	25.4	25.8	25.4	25.4	24.9	27.8	26.7	26.9	26.8	27.3	25.3	26.9	0.0	-1.6	3.8E-03
MYO9B	24.9	26.0	24.9	24.3	25.3	25.0	24.6	25.2	24.6	26.0	26.5	25.9	26.2	25.2	25.8	28.3	26.7	26.9	26.9	26.8	25.2	26.8	0.0	-1.6	3.8E-03
TYMS	24.8	26.9	26.5	26.0	27.4	25.6	25.3	26.4	26.6	26.7	27.0	26.5	26.6	26.4	27.9	27.9	28.3	28.9	28.5	26.7	26.5	28.1	0.0	-1.6	3.8E-03
GYG1	24.9	22.4	24.9	25.5	24.2	24.5	24.2	24.6	24.1	24.7	24.8	25.1	25.8	25.2	26.0	27.0	26.5	29.1	26.2	25.8	24.8	26.4	0.0	-1.6	3.9E-03
FOXK1	24.3	23.8	23.7	23.5	24.6	24.2	22.9	NaN	24.3	24.2	24.4	23.0	23.5	25.1	25.0	25.8	26.2	24.3	25.8	25.9	24.2	25.8	0.0	-1.6	3.9E-03
CORO7	25.8	26.6	26.4	25.7	25.2	25.4	26.0	25.7	26.7	26.2	26.3	25.5	27.1	25.9	26.4	28.5	27.4	27.6	27.0	27.9	25.9	27.5	0.0	-1.6	4.0E-03
DCTN3	25.3	25.9	26.3	25.0	25.2	24.2	25.4	25.3	25.1	25.9	25.7	25.9	25.8	25.6	26.4	27.7	27.0	27.2	26.5	27.2	25.5	27.1	0.0	-1.6	4.0E-03

RPS4Y1	22.6	28.2	26.6	27.1	26.7	26.4	NaN	21.6	26.5	22.8	22.6	28.3	27.8	26.7	26.9	28.0	28.9	27.6	28.8	28.3	26.6	28.2	0.0	-1.6	4.2E-03
GNS	26.7	27.0	26.4	26.5	26.5	26.4	26.2	26.6	26.9	27.0	26.7	27.1	26.9	27.1	27.6	29.6	28.2	28.5	27.7	29.7	26.7	28.3	0.0	-1.6	4.4E-03
MTR	24.8	27.1	26.6	25.8	24.7	25.8	25.6	26.3	25.6	26.5	26.4	25.7	27.1	25.8	27.1	27.3	27.5	26.9	27.5	29.0	25.8	27.4	0.0	-1.6	4.5E-03
ARSB	24.4	24.2	23.9	24.3	23.3	23.9	24.0	NaN	23.7	23.8	23.9	24.7	23.9	23.8	25.0	25.6	24.8	25.9	25.3	26.4	23.9	25.5	0.0	-1.6	4.5E-03
CDK5RAP3	24.4	24.6	25.3	24.9	23.8	23.5	25.3	25.5	24.1	25.1	25.6	24.7	25.6	24.0	25.8	26.5	26.5	26.6	26.0	26.2	24.8	26.3	0.0	-1.6	4.8E-03
PLEC	30.3	30.9	30.0	30.8	30.6	30.9	30.6	30.6	30.4	30.3	30.3	30.4	30.4	30.4	30.1	32.7	31.7	32.2	31.3	32.4	30.4	32.0	0.0	-1.5	4.9E-03
DBNL	27.4	27.3	27.6	26.9	27.2	27.3	27.3	27.3	27.3	28.3	28.0	27.3	27.5	27.8	28.4	29.6	28.9	28.9	28.5	29.2	27.3	28.9	0.0	-1.5	5.2E-03
NHLRC2	26.3	26.3	26.4	25.6	26.0	25.3	26.3	26.6	26.1	26.3	26.5	25.6	27.3	25.2	27.5	28.2	28.0	27.8	27.9	27.4	26.3	27.8	0.0	-1.5	5.3E-03
ARPC5	27.7	27.9	27.6	28.2	27.4	27.3	27.9	27.8	27.6	27.9	27.8	28.0	27.8	27.8	28.2	29.3	29.0	29.3	29.3	29.5	27.8	29.3	0.0	-1.5	6.0E-03
COMMD9	24.3	24.8	26.4	25.7	25.4	25.1	24.9	24.7	25.0	25.8	24.7	26.0	24.1	24.5	26.3	26.7	26.2	26.6	26.6	26.3	24.9	26.4	0.0	-1.5	6.3E-03
PLOD3	27.1	27.1	26.7	26.7	27.4	27.4	26.4	27.0	27.3	27.1	27.0	27.2	27.6	27.4	27.4	28.7	29.0	28.5	27.9	29.2	27.1	28.6	0.0	-1.5	6.5E-03
SLK	25.8	25.8	26.1	26.0	25.9	25.5	25.5	26.9	26.1	25.9	26.1	26.1	26.3	25.5	27.4	27.4	27.4	27.5	28.8	27.4	26.0	27.4	0.0	-1.5	6.6E-03
AK3	26.3	25.9	25.4	25.3	24.5	21.5	26.4	25.2	25.7	25.9	25.7	26.1	26.2	26.4	27.3	27.1	26.2	27.4	27.2	28.5	25.8	27.3	0.0	-1.5	6.6E-03
PEF1	24.6	24.8	26.2	25.5	25.8	24.5	24.9	24.9	25.4	24.8	25.5	24.8	25.5	24.6	25.9	26.6	26.6	26.0	26.4	26.3	24.9	26.4	0.0	-1.5	7.2E-03
TMEM2	25.0	25.5	24.6	25.8	25.5	25.1	25.3	25.2	25.1	25.3	26.3	25.2	25.5	25.9	26.1	26.7	26.5	27.8	27.0	26.8	25.3	26.8	0.0	-1.5	7.3E-03
GNAS	27.4	27.8	27.8	28.3	27.8	28.0	27.7	27.6	27.5	27.6	28.1	28.0	27.9	27.9	28.3	29.6	28.6	29.5	29.1	29.4	27.8	29.3	0.0	-1.5	7.5E-03
LIMS1	25.8	25.2	25.8	26.2	25.0	24.3	25.9	25.7	24.4	26.1	25.8	25.8	25.8	25.3	25.9	27.4	27.5	27.5	27.0	26.8	25.8	27.2	0.0	-1.5	7.6E-03
P4HA1	28.1	28.1	26.7	28.1	27.2	27.2	27.3	27.4	27.4	27.5	27.4	27.8	28.4	28.4	28.1	29.1	29.7	28.4	28.7	30.3	27.5	28.9	0.0	-1.5	7.6E-03
BIN1	25.1	25.8	26.6	25.1	NaN	NaN	24.6	24.7	25.9	26.0	26.1	26.3	25.5	24.9	26.8	26.7	26.8	27.4	27.4	28.2	25.7	27.1	0.0	-1.5	7.6E-03
CHMP3	23.4	23.5	23.6	23.4	24.0	23.3	23.0	23.4	NaN	23.9	23.9	24.0	23.1	23.3	24.3	25.6	24.7	24.9	25.0	24.9	23.4	24.9	0.0	-1.5	7.7E-03
SUCLG2	26.1	26.4	25.8	26.5	25.4	25.2	25.7	25.5	25.9	25.9	26.0	26.8	26.5	26.2	26.5	27.3	27.6	26.9	27.4	27.8	25.9	27.4	0.0	-1.5	7.8E-03
CTSB	25.6	24.4	24.1	24.6	24.5	26.0	25.8	25.6	25.1	26.1	25.8	25.9	26.1	25.6	26.5	28.3	27.3	26.0	26.7	28.8	25.6	27.0	0.0	-1.4	8.0E-03
COLGALT1	27.4	27.5	27.1	27.1	27.8	27.8	27.0	27.7	27.4	27.4	27.6	27.8	28.2	27.5	28.2	29.2	29.2	28.7	28.6	30.0	27.5	28.9	0.0	-1.4	8.3E-03
ANP32B	28.4	29.0	29.2	29.4	28.0	28.0	29.4	28.4	28.3	29.3	29.1	29.5	29.7	29.3	30.6	30.4	30.6	30.2	31.0	31.1	29.2	30.6	0.0	-1.4	8.8E-03
MARCH5	23.7	23.8	23.0	23.0	22.9	22.9	22.8	23.6	23.4	22.6	23.8	24.9	22.8	22.4	23.2	23.2	24.9	23.9	25.2	24.9	23.0	24.4	0.0	-1.4	9.0E-03
SLC35B2	25.4	25.3	25.3	26.2	25.1	25.1	25.4	24.8	25.1	25.5	25.5	25.3	25.5	25.2	25.8	26.8	26.9	26.8	26.4	26.7	25.3	26.7	0.0	-1.4	9.4E-03
UAP1	25.3	25.0	25.2	24.3	25.2	25.0	25.1	25.3	25.2	24.5	25.3	25.4	25.7	25.4	25.1	26.7	27.1	27.1	26.5	26.2	25.2	26.6	0.0	-1.4	9.5E-03
GNAI1	24.0	24.8	24.2	23.8	23.5	NaN	23.4	24.9	23.2	24.4	24.1	24.3	25.1	24.6	25.3	24.7	24.7	25.9	26.6	26.8	24.2	25.6	0.0	-1.4	9.6E-03
FKBP10	28.8	28.6	28.8	29.4	29.2	28.8	28.6	28.4	28.7	29.0	28.6	29.2	28.6	28.6	29.3	30.4	30.5	30.0	29.4	31.6	28.8	30.2	0.0	-1.4	9.7E-03
ARHGDIA	30.5	30.3	30.4	30.4	30.3	30.1	30.5	30.4	30.3	30.6	30.6	30.3	30.6	30.7	31.0	32.4	31.8	31.9	31.8	32.0	30.4	31.8	0.0	-1.4	9.7E-03
LARP7	25.1	25.8	24.6	25.1	25.5	25.6	26.1	25.5	24.5	26.7	25.6	25.2	25.4	24.9	26.1	26.5	27.2	25.8	27.3	27.4	25.4	26.8	0.0	-1.4	9.7E-03
RER1	24.7	24.9	25.5	25.4	24.9	24.2	24.5	24.9	24.1	24.3	25.2	25.1	25.9	24.8	24.5	26.5	26.2	26.6	26.0	26.3	24.9	26.3	0.0	-1.4	1.0E-02
QIL1	25.2	25.5	25.1	26.2	25.9	24.6	25.2	24.8	25.8	25.9	25.1	26.1	25.4	25.5	26.0	26.9	26.6	26.9	26.9	26.8	25.4	26.8	0.0	-1.4	1.0E-02
INPPL1	24.7	25.1	25.7	25.3	24.2	25.9	25.4	25.0	25.3	26.0	26.2	24.3	25.9	25.0	26.1	27.2	26.8	27.2	25.6	26.5	25.3	26.7	0.0	-1.4	1.0E-02
ECHDC1	27.1	26.0	26.2	25.4	24.4	26.5	25.3	23.8	25.7	26.6	25.8	26.0	26.6	26.6	26.4	27.2	27.5	27.2	27.5	27.8	26.0	27.4	0.0	-1.4	1.1E-02
PPP4R1	25.5	26.0	25.7	26.3	26.0	26.0	25.2	26.0	25.7	25.8	25.7	25.8	25.6	25.7	26.5	27.1	26.3	28.0	27.4	27.2	25.8	27.1	0.0	-1.4	1.1E-02
ERGIC3	24.0	NaN	23.6	24.9	24.1	22.8	24.1	23.5	23.8	24.0	24.1	24.4	24.4	23.7	24.7	25.2	25.4	25.5	25.4	25.8	24.0	25.4	0.0	-1.4	1.1E-02
ADAM9	NaN	NaN	23.0	23.1	NaN	NaN	22.8	NaN	23.4	24.1	22.6	23.3	21.9	NaN	23.6	26.7	23.4	27.1	25.1	23.7	23.0	24.4	0.0	-1.4	1.1E-02
GLIPR2	24.2	NaN	23.7	NaN	24.5	24.7	24.2	NaN	24.2	24.5	24.6	NaN	24.7	23.6	24.0	27.5	25.3	26.2	25.6	25.8	24.4	25.7	0.0	-1.4	1.1E-02
CCDC22	NaN	NaN	23.1	23.0	22.2	NaN	24.0	NaN	22.8	23.2	23.1	22.7	NaN	NaN	23.8	24.8	24.0	25.8	25.3	23.8	23.0	24.4	0.0	-1.4	1.1E-02
FAM213A	27.6	28.1	28.1	28.3	27.9	28.0	27.3	27.6	27.9	28.4	28.3	27.9	28.2	27.8	28.4	29.5	28.9	29.3	29.3	29.7	27.9	29.3	0.0	-1.4	1.1E-02
CLCN3	22.4	21.8	21.9	22.1	21.5	21.7	22.4	22.8	21.7	21.8	22.1	22.0	22.6	22.7	21.8	24.3	23.4	23.4	22.3	24.5	22.0	23.4	0.0	-1.4	1.2E-02

CHMP6	24.8	25.5	25.1	24.9	24.8	24.5	24.5	24.2	24.5	25.3	25.1	25.1	24.8	24.3	25.1	27.1	25.8	26.2	26.1	26.3	24.8	26.1	0.0	-1.4	1.2E-02
RAP1GDS1	27.6	28.2	28.4	27.0	27.7	27.7	27.3	27.8	27.4	28.3	28.0	27.6	28.2	28.0	28.5	29.8	29.2	29.0	29.0	30.6	27.8	29.1	0.0	-1.3	1.2E-02
MKL2	23.6	NaN	23.3	23.1	23.7	NaN	NaN	NaN	23.5	23.3	24.6	NaN	23.4	NaN	24.0	26.0	25.6	24.3	23.5	25.2	23.4	24.8	0.0	-1.3	1.2E-02
LIMD2	24.7	24.8	23.8	23.2	24.7	25.3	25.4	NaN	24.7	26.3	25.2	23.2	24.1	24.5	26.0	26.5	26.8	24.9	26.0	25.6	24.7	26.0	0.0	-1.3	1.3E-02
B3GAT3	25.0	23.9	24.2	NaN	24.1	NaN	24.5	24.0	24.5	24.3	24.8	24.1	24.9	24.8	25.2	26.3	25.4	26.0	25.1	26.1	24.4	25.7	0.0	-1.3	1.3E-02
STAM2	23.5	23.6	25.0	NaN	24.1	NaN	23.5	NaN	23.3	24.0	23.2	NaN	23.2	23.7	24.1	25.7	25.2	NaN	NaN	24.6	23.5	24.9	0.0	-1.3	1.3E-02
NAGK	26.1	26.8	27.0	27.6	27.0	26.4	26.9	26.8	26.3	27.0	26.8	26.5	26.8	26.8	27.2	29.1	27.8	28.5	27.5	28.4	26.8	28.1	0.0	-1.3	1.3E-02
PRKAA1	25.8	25.1	25.0	25.6	25.8	25.4	25.7	25.4	25.2	25.9	25.8	25.1	25.6	25.9	26.4	26.9	27.0	26.9	26.7	27.6	25.6	26.9	0.0	-1.3	1.4E-02
GUK1	23.9	23.9	23.8	24.3	23.4	NaN	23.9	24.8	NaN	23.8	23.3	25.0	24.1	24.0	24.0	25.8	24.8	26.2	24.6	25.6	23.9	25.2	0.0	-1.3	1.4E-02
AIP	25.2	26.1	25.7	26.2	25.5	26.1	25.8	26.4	25.6	25.5	25.6	25.8	25.8	25.4	26.8	26.9	27.5	27.0	27.6	27.2	25.8	27.1	0.0	-1.3	1.4E-02
USP47	26.5	26.4	26.6	26.9	27.2	27.6	26.4	26.3	27.4	26.6	26.7	26.0	27.0	26.9	27.0	28.6	27.9	28.3	28.0	27.7	26.7	28.0	0.0	-1.3	1.5E-02
IDI1	28.1	28.1	29.5	29.0	27.7	27.5	27.9	28.2	28.0	28.7	28.7	28.6	28.3	28.4	28.9	29.5	29.6	30.8	30.3	29.1	28.2	29.5	0.0	-1.3	1.5E-02
LSS	28.8	28.8	28.9	29.1	28.3	28.4	28.4	28.4	28.3	28.8	28.9	29.1	28.4	28.5	29.0	30.4	29.7	30.4	30.2	29.4	28.6	29.9	0.0	-1.3	1.5E-02
CMPK1	27.1	27.5	27.1	26.6	27.0	27.1	27.3	27.5	27.3	27.5	27.5	27.3	27.9	27.5	28.0	29.1	29.0	28.3	28.4	28.8	27.3	28.6	0.0	-1.3	1.5E-02
PRKACA	26.6	26.1	26.6	26.2	26.5	26.0	26.4	26.9	26.1	26.3	26.9	26.8	27.1	27.0	27.0	28.2	28.0	27.7	27.2	27.9	26.5	27.8	0.0	-1.3	1.5E-02
GSN	29.1	29.0	29.9	28.3	29.1	29.2	29.1	28.7	29.3	29.0	29.3	28.5	29.9	29.0	29.6	32.3	31.4	29.4	29.4	31.1	29.1	30.4	0.0	-1.3	1.5E-02
RNH1	29.1	29.4	29.5	28.8	28.6	28.2	29.0	29.7	29.1	29.3	29.1	29.6	29.1	29.8	29.8	31.1	30.2	30.2	30.7	31.6	29.1	30.4	0.0	-1.3	1.5E-02
FTH1	29.5	29.7	30.8	28.2	28.6	29.3	28.9	29.5	28.0	28.9	29.4	28.4	30.2	29.8	29.9	30.7	31.2	30.1	30.5	30.8	29.3	30.6	0.0	-1.3	1.5E-02
MON2	24.1	24.8	24.7	23.7	NaN	22.7	24.5	24.2	23.2	24.5	24.4	25.0	25.5	24.2	24.4	25.5	25.7	25.8	26.2	25.8	24.4	25.7	0.0	-1.3	1.6E-02
RAC3	23.4	24.9	22.5	23.0	23.0	23.3	23.3	24.5	23.2	23.0	23.6	24.3	24.6	23.3	23.4	25.4	25.4	24.1	25.1	23.8	23.3	24.6	0.0	-1.3	1.6E-02
KIF5B	29.5	29.8	29.5	29.5	29.3	29.1	29.5	29.6	29.6	29.7	29.8	29.7	30.0	29.8	30.2	31.1	31.0	31.0	30.7	30.8	29.6	30.9	0.0	-1.3	1.6E-02
PPP1R18	23.2	NaN	21.7	23.5	22.4	NaN	22.8	23.1	22.3	23.1	23.2	21.8	22.5	23.0	23.0	25.7	25.1	23.1	24.4	24.0	22.9	24.2	0.0	-1.3	1.6E-02
OVCA2	23.6	23.7	25.9	25.5	23.7	24.4	24.0	23.7	23.2	24.1	24.4	24.2	25.2	25.0	23.9	25.3	25.3	27.1	25.6	25.5	24.2	25.4	0.0	-1.3	1.7E-02
CKAP4	30.5	30.7	30.5	30.9	30.7	30.5	30.3	30.4	30.7	30.7	30.8	30.9	30.9	30.8	31.2	32.2	32.4	31.7	31.5	32.8	30.7	32.0	0.0	-1.3	1.7E-02
DIP2B	24.1	24.8	24.4	24.3	23.6	22.9	24.1	24.0	24.5	25.1	24.6	24.8	24.7	24.3	25.0	26.2	25.7	24.6	25.5	25.9	24.4	25.6	0.0	-1.3	1.7E-02
ATP2B4	23.9	23.9	23.0	22.4	NaN	23.0	24.9	23.7	23.3	24.0	25.4	24.1	23.3	23.8	24.5	27.3	24.3	25.7	23.7	27.1	23.8	25.1	0.0	-1.3	1.7E-02
RBBP9	23.6	24.6	NaN	NaN	23.9	NaN	24.0	NaN	24.0	23.9	24.1	24.6	24.7	24.2	24.8	26.8	25.7	24.6	25.0	25.6	24.0	25.3	0.0	-1.3	1.7E-02
GBAS	26.5	24.6	26.0	26.6	27.1	27.1	26.2	25.9	25.7	26.3	26.6	25.8	27.0	26.4	27.0	28.6	27.7	27.5	27.6	27.9	26.4	27.6	0.0	-1.3	1.7E-02
NAGA	24.7	NaN	24.5	23.6	NaN	NaN	24.7	22.3	24.9	24.5	24.5	25.8	25.9	25.5	24.8	27.8	25.9	25.0	26.0	26.7	24.7	26.0	0.0	-1.3	1.8E-02
NIT2	26.5	26.3	26.0	26.0	26.7	26.3	26.9	26.1	25.5	26.0	25.7	26.1	26.7	26.4	27.0	27.0	27.9	26.3	28.0	28.9	26.2	27.5	0.0	-1.3	1.8E-02
YIPF5	23.7	23.3	22.9	23.6	24.3	24.6	23.7	23.1	23.5	23.8	24.0	23.1	23.6	24.0	NaN	25.0	25.0	24.2	24.1	24.9	23.7	24.9	0.0	-1.2	1.8E-02
HEBP2	24.1	23.9	24.6	25.2	25.7	NaN	24.7	24.4	NaN	24.6	24.5	24.3	24.9	24.8	25.5	24.6	24.8	26.7	26.3	26.2	24.6	25.8	0.0	-1.2	1.9E-02
MAPKAPK2	22.9	22.9	22.7	23.2	22.1	NaN	23.0	NaN	23.1	22.3	22.8	NaN	23.0	23.1	22.8	24.3	24.3	23.9	22.9	24.4	22.9	24.1	0.0	-1.2	1.9E-02
TUBA1A	31.2	31.0	30.5	30.0	30.2	30.5	31.0	30.8	31.0	31.2	31.7	30.9	31.6	31.3	32.1	33.7	32.3	32.1	31.7	32.3	31.0	32.2	0.0	-1.2	1.9E-02
PEG10	26.1	25.4	26.4	26.2	26.0	25.7	26.3	25.3	25.7	26.2	25.8	26.2	26.0	25.4	26.9	27.8	27.3	28.1	27.1	26.0	26.0	27.2	0.0	-1.2	1.9E-02
ANTXR1	24.0	23.4	23.4	24.2	23.9	23.1	23.5	23.2	23.6	24.2	23.9	23.5	23.5	24.2	24.4	25.7	24.8	24.5	24.8	25.3	23.6	24.8	0.0	-1.2	2.0E-02
DPYSL4	26.0	27.8	26.4	23.7	26.1	26.2	26.4	26.2	26.2	27.5	28.0	26.4	27.0	27.0	28.1	30.1	26.7	29.0	27.1	27.1	26.4	27.6	0.0	-1.2	2.0E-02
DCTN6	23.6	24.9	24.0	24.6	23.6	23.8	24.1	25.5	24.0	24.7	24.0	24.4	24.1	23.5	24.7	25.6	25.2	25.8	25.3	25.3	24.1	25.3	0.0	-1.2	2.0E-02
TMEM109	25.5	25.9	25.9	26.5	26.8	26.8	25.5	25.7	25.4	26.4	26.1	26.1	26.0	26.1	26.8	27.3	27.8	27.8	27.2	27.1	26.0	27.3	0.0	-1.2	2.0E-02
DTX3	23.6	NaN	24.2	24.3	23.8	NaN	23.7	23.9	23.2	24.0	23.8	24.5	23.7	NaN	24.6	25.6	NaN	25.1	24.9	25.4	23.8	25.1	0.0	-1.2	2.0E-02
MAP1LC3B	27.2	27.5	27.2	27.0	27.1	27.5	27.6	27.6	27.1	27.5	27.8	27.7	28.0	27.9	27.7	29.0	28.9	29.0	28.5	28.3	27.5	28.7	0.0	-1.2	2.0E-02
ILK	28.0	28.0	27.6	28.8	28.0	28.1	28.0	28.1	27.8	28.2	27.9	27.9	28.0	28.1	28.2	29.6	29.6	29.4	29.1	28.8	28.0	29.2	0.0	-1.2	2.0E-02

ELOVL1	25.8	25.1	25.3	26.3	20.3	28.1	25.4	25.3	25.3	25.6	25.7	25.9	25.2	25.2	25.7	26.1	26.8	27.1	26.8	26.4	25.4	26.6	0.0	-1.2	2.1E-02
KCTD15	23.4	24.3	24.5	24.0	23.2	23.3	23.3	24.6	23.6	23.2	24.3	24.9	24.6	23.8	25.3	25.1	23.7	24.6	25.6	25.1	23.9	25.1	0.0	-1.2	2.1E-02
TRIP13	26.5	27.7	27.3	27.4	25.5	26.5	26.5	27.1	25.9	26.8	26.6	26.7	27.2	26.7	27.0	27.2	28.2	28.1	28.3	27.8	26.7	27.9	0.0	-1.2	2.2E-02
PDPK2P	22.3	NaN	22.2	NaN	21.4	22.1	21.3	26.4	22.2	21.9	22.6	22.1	22.0	22.2	24.4	23.0	22.7	24.1	NaN	23.3	22.1	23.3	0.0	-1.2	2.2E-02
SEC14L1	22.5	24.0	24.1	23.4	22.7	NaN	22.2	NaN	22.4	24.4	24.5	24.0	22.8	NaN	24.0	26.1	24.5	27.1	23.8	24.8	23.4	24.6	0.0	-1.2	2.2E-02
PDYN	25.2	25.3	26.8	26.1	NaN	25.2	25.5	25.0	25.2	25.6	26.0	26.0	26.4	25.6	26.0	27.0	26.8	27.3	26.7	26.6	25.6	26.8	0.0	-1.2	2.2E-02
LAMP2	25.6	25.4	25.6	25.8	24.0	23.7	25.8	26.1	25.2	25.8	25.4	25.9	26.0	25.2	25.8	27.0	25.3	27.3	26.5	27.7	25.6	26.8	0.0	-1.2	2.2E-02
SEC22B	27.9	28.3	27.7	27.6	28.3	28.2	27.8	27.9	28.0	28.1	28.1	28.1	27.8	28.1	28.6	29.5	29.5	28.9	28.8	29.8	28.0	29.2	0.0	-1.2	2.3E-02
PBX1	23.8	19.2	NaN	23.6	23.7	24.2	23.5	NaN	23.5	23.7	23.3	NaN	23.2	23.8	24.5	24.8	24.8	24.1	NaN	26.5	23.6	24.8	0.0	-1.2	2.3E-02
CRELD2	24.1	24.8	24.4	24.5	24.0	23.5	23.9	24.1	23.9	24.4	24.4	25.3	24.3	24.9	24.3	26.2	25.7	25.0	25.4	26.1	24.4	25.6	0.0	-1.2	2.4E-02
TLN1	31.6	31.9	31.8	31.9	32.0	32.1	31.9	31.9	31.6	31.9	31.7	31.3	30.8	31.9	32.0	33.4	33.0	33.6	32.9	33.1	31.9	33.1	0.0	-1.2	2.4E-02
APOL2	22.5	23.0	22.9	23.5	23.9	23.5	23.2	23.5	23.4	22.7	22.4	22.7	22.4	21.8	23.2	24.8	24.5	23.7	23.5	24.8	23.0	24.1	0.0	-1.2	2.5E-02
PSMD9	25.2	25.0	NaN	25.0	24.8	24.3	25.9	25.5	24.8	25.6	25.6	24.7	24.2	24.8	25.4	26.1	26.0	26.1	26.6	26.6	25.0	26.1	0.0	-1.2	2.5E-02
FXR2	26.1	26.5	25.6	24.5	25.7	26.6	26.4	26.4	26.0	26.2	26.5	25.8	26.1	26.1	26.8	27.4	27.5	27.4	26.8	27.2	26.1	27.3	0.0	-1.2	2.5E-02
WIZ	26.2	26.0	26.1	25.7	26.1	NaN	25.9	23.5	25.5	26.8	26.6	24.0	25.4	25.1	27.0	26.8	27.8	26.6	27.2	27.2	25.9	27.1	0.0	-1.2	2.6E-02
CPPED1	25.4	25.5	25.0	24.2	25.5	NaN	25.2	24.0	25.4	25.6	24.8	24.9	22.8	25.5	25.5	26.0	26.6	25.3	27.3	27.5	25.2	26.3	0.0	-1.2	2.6E-02
HINT2	26.2	25.9	27.1	26.9	25.7	25.4	26.1	26.5	26.0	26.5	26.2	26.6	26.6	26.6	27.2	27.4	26.8	27.7	27.6	27.7	26.4	27.5	0.0	-1.2	2.6E-02
ITGB1	29.8	29.3	29.5	30.0	29.5	29.0	29.7	29.4	29.1	29.7	29.6	29.7	29.6	29.5	29.7	30.8	30.6	31.1	30.3	30.8	29.5	30.7	0.0	-1.2	2.6E-02
GSTO1	27.8	27.7	28.2	28.3	27.8	28.3	28.5	28.1	27.1	27.9	28.2	27.2	28.7	28.8	28.6	29.2	29.8	29.3	29.4	29.6	28.2	29.3	0.0	-1.2	2.6E-02
DECR1	27.6	27.4	27.6	28.1	27.4	27.7	27.7	27.1	27.3	27.3	27.5	27.6	28.1	28.0	27.9	29.6	28.5	28.7	28.7	28.8	27.6	28.7	0.0	-1.2	2.6E-02
DDAH2	27.6	28.0	27.3	27.0	27.6	28.0	27.5	28.2	27.7	27.8	28.3	27.4	28.6	28.4	29.0	30.5	28.9	29.4	28.6	28.1	27.8	29.0	0.0	-1.2	2.7E-02
CGGBP1	24.5	23.9	24.1	24.1	23.7	24.1	23.9	24.1	24.6	25.2	25.4	23.3	24.9	24.4	25.3	25.3	25.7	25.7	25.1	25.1	24.1	25.3	0.0	-1.1	2.7E-02
GOLIM4	25.5	25.8	24.9	26.3	26.4	26.1	26.1	25.6	26.2	25.9	25.8	26.3	26.2	25.8	26.7	27.2	27.8	26.9	27.1	27.3	26.0	27.1	0.0	-1.1	2.7E-02
BAZ1A	24.3	24.0	24.1	24.0	24.7	25.3	24.3	23.9	24.6	24.5	24.6	25.8	24.4	24.3	26.1	25.5	25.6	25.0	26.9	25.3	24.4	25.5	0.0	-1.1	2.8E-02
NEMF	25.4	25.3	25.0	25.2	25.1	25.1	25.3	26.3	25.5	25.5	25.4	25.9	25.9	25.2	25.6	26.3	25.7	26.6	26.8	26.7	25.3	26.5	0.0	-1.1	2.8E-02
FDPS	30.3	30.4	30.6	30.4	30.3	30.5	30.4	30.6	30.2	30.2	30.8	29.8	31.1	30.9	30.6	31.9	32.2	31.8	31.3	31.1	30.4	31.5	0.0	-1.1	2.8E-02
DOCK1	24.9	24.2	24.2	24.6	24.6	24.1	24.9	24.5	24.6	25.2	24.5	23.8	25.3	23.9	25.6	26.1	26.2	25.7	24.8	25.4	24.5	25.7	0.0	-1.1	2.8E-02
HECTD3	23.2	NaN	23.3	23.7	NaN	NaN	23.4	22.9	NaN	23.2	23.8	22.9	23.1	22.9	23.8	25.5	24.5	23.8	24.3	24.5	23.2	24.4	0.0	-1.1	2.8E-02
GSS	27.5	27.4	27.8	27.4	26.9	27.0	28.2	27.8	27.5	27.7	27.7	27.6	28.2	27.9	28.1	29.0	28.8	28.6	28.8	29.4	27.7	28.8	0.0	-1.1	2.8E-02
DCTN1	28.5	28.4	28.9	29.1	28.5	27.4	28.9	29.1	28.3	28.5	28.6	28.0	28.7	28.5	29.2	30.5	29.5	30.1	29.1	29.8	28.5	29.7	0.0	-1.1	2.9E-02
GDI1	28.8	29.2	28.9	28.9	29.1	29.4	28.9	29.1	29.0	29.2	29.3	29.0	29.5	29.3	29.7	31.7	29.9	30.9	30.0	30.4	29.1	30.2	0.0	-1.1	2.9E-02
SH3BGRL	25.9	26.4	27.2	25.8	25.6	26.0	26.3	26.6	26.3	26.8	26.4	26.2	26.2	25.8	26.9	27.3	26.7	28.2	27.4	27.7	26.2	27.4	0.0	-1.1	2.9E-02
BCS1L	24.6	25.1	24.4	23.5	24.6	NaN	23.7	23.2	24.1	25.0	24.4	25.3	24.4	23.5	25.6	25.7	25.4	24.3	NaN	25.9	24.4	25.6	0.0	-1.1	2.9E-02
VAMP3	26.4	26.0	26.2	26.7	25.7	26.0	26.2	25.6	26.0	26.5	26.3	26.2	26.6	26.2	26.8	27.3	27.3	27.5	27.3	27.7	26.2	27.3	0.0	-1.1	2.9E-02
CSK	26.4	27.0	26.5	26.5	26.7	26.9	26.4	27.3	26.6	26.8	26.9	26.8	27.2	26.8	27.5	28.0	27.7	27.8	28.6	28.1	26.8	27.9	0.0	-1.1	3.0E-02
NENF	24.1	25.0	23.9	24.3	23.1	24.6	24.0	24.3	24.1	23.7	24.3	24.7	23.8	24.2	24.0	25.6	24.1	25.7	25.0	25.8	24.2	25.3	0.0	-1.1	3.1E-02
ARFGAP3	23.8	23.8	22.8	NaN	24.2	24.5	23.6	24.3	24.0	23.5	NaN	NaN	NaN	24.0	24.1	25.6	25.7	25.0	23.9	25.1	23.9	25.0	0.0	-1.1	3.1E-02
RB1	24.2	24.3	23.5	24.0	23.7	24.1	24.1	23.6	24.3	24.6	24.5	24.3	24.2	24.3	24.6	26.3	26.0	25.4	24.8	25.3	24.2	25.3	0.0	-1.1	3.1E-02
LZTFL1	23.7	24.4	23.7	24.2	24.0	NaN	23.8	25.7	NaN	23.7	23.6	23.8	24.2	23.7	24.2	25.7	24.4	24.6	25.2	25.7	23.8	24.9	0.0	-1.1	3.2E-02
MYDGF	27.6	27.8	27.2	27.7	27.6	27.9	27.5	27.6	27.6	27.8	27.5	27.7	27.8	27.7	27.9	28.8	29.0	28.9	28.5	28.7	27.6	28.7	0.0	-1.1	3.2E-02
SLC35E1	24.0	NaN	24.5	25.2	23.5	24.2	23.3	NaN	23.8	24.2	24.0	23.8	23.7	NaN	24.4	24.5	25.4	25.5	24.7	25.9	24.0	25.1	0.0	-1.1	3.2E-02
GMPPB	26.5	26.8	27.0	27.3	26.8	26.9	27.0	27.2	26.8	26.9	26.8	26.8	27.1	27.0	27.1	28.0	28.1	28.3	28.1	27.9	26.9	28.0	0.0	-1.1	3.3E-02

ACOX1	25.2	25.6	25.9	26.5	24.9	NaN	25.7	25.6	25.5	26.0	25.4	27.0	25.6	25.2	26.0	26.9	26.2	26.5	27.6	27.3	25.6	26.7	0.0	-1.1	3.3E-02
SLC12A2	23.9	22.7	23.9	23.8	23.9	24.3	23.7	23.9	24.3	24.6	24.8	23.3	23.8	23.8	24.8	25.6	24.8	26.4	24.2	25.2	23.9	25.0	0.0	-1.1	3.3E-02
CDK4	24.6	25.9	25.1	24.9	24.1	24.2	25.0	24.5	24.4	25.0	25.0	25.6	24.9	24.4	26.0	24.9	26.0	25.7	26.8	28.5	24.9	26.0	0.0	-1.1	3.4E-02
ATP2B1	26.5	26.8	26.6	27.1	26.7	26.2	26.3	25.8	26.6	26.9	26.9	26.9	26.9	26.7	27.3	28.0	28.1	27.4	27.6	28.5	26.7	27.8	0.0	-1.1	3.4E-02
BICD2	22.9	24.1	23.6	23.3	23.5	NaN	23.4	23.1	23.3	23.3	23.4	22.7	23.4	23.1	23.6	24.6	24.0	25.9	25.3	24.2	23.3	24.4	0.0	-1.1	3.4E-02
PTMS	28.7	29.3	28.6	28.9	29.1	28.7	29.0	28.8	29.4	29.0	29.3	28.1	29.3	29.1	29.9	30.1	30.8	30.1	28.8	30.1	29.0	30.1	0.0	-1.1	3.5E-02
TRIM2	24.8	25.8	26.4	26.2	25.9	25.9	25.6	26.0	25.4	25.9	25.8	25.6	25.9	25.8	25.9	27.0	25.9	27.3	26.9	27.0	25.8	26.9	0.0	-1.1	3.5E-02
TMX4	23.0	22.9	22.7	22.3	23.2	23.1	22.7	21.9	22.9	22.1	23.0	23.2	23.5	NaN	23.6	25.2	24.5	22.7	23.0	24.4	22.9	24.0	0.0	-1.1	3.5E-02
JAM3	22.5	22.6	22.9	22.7	NaN	NaN	NaN	22.3	NaN	22.7	22.9	22.6	23.0	23.2	23.9	23.6	24.5	23.8	23.5	24.6	22.7	23.8	0.0	-1.1	3.5E-02
TP53I3	27.0	27.5	28.2	27.6	26.6	26.9	27.1	27.3	27.0	27.6	27.7	27.2	27.6	26.8	27.4	28.7	28.5	29.1	27.8	28.1	27.2	28.3	0.0	-1.1	3.6E-02
TERF2IP	25.4	25.1	25.0	24.4	24.4	25.2	24.6	25.3	24.9	25.3	25.3	24.9	24.9	25.6	26.2	26.2	26.0	25.6	25.7	26.5	25.0	26.1	0.0	-1.1	3.7E-02
CYB5R1	23.3	23.9	23.5	23.9	24.1	NaN	23.8	24.2	23.6	23.4	23.3	23.9	24.3	24.0	23.7	26.0	25.4	24.0	24.5	25.4	23.9	24.9	0.0	-1.1	3.7E-02
TAGLN2	29.5	28.7	27.9	28.9	28.8	29.2	29.5	29.2	29.1	29.1	28.6	28.5	28.7	29.2	29.4	30.6	29.7	30.4	31.1	29.3	29.0	30.0	0.0	-1.1	3.7E-02
TSG101	25.9	26.8	26.0	26.3	25.9	26.2	26.1	26.7	26.0	26.1	26.2	26.1	26.0	26.0	26.5	27.4	26.6	27.1	27.2	27.2	26.1	27.1	0.0	-1.1	3.8E-02
TFG	26.4	26.4	26.3	27.1	26.7	26.8	26.2	26.5	26.5	26.9	26.7	26.5	26.6	26.5	26.9	27.6	27.7	27.7	27.4	27.6	26.5	27.6	0.0	-1.1	3.8E-02
NEDD1	24.5	24.1	22.4	24.4	24.7	24.2	24.1	23.2	23.8	24.6	24.3	23.4	23.9	24.3	24.6	25.8	25.4	25.1	25.3	23.8	24.1	25.2	0.0	-1.1	3.9E-02
ERAP1	24.0	25.2	24.2	24.0	24.4	24.5	24.8	25.2	23.9	24.4	24.3	23.9	24.0	23.8	24.5	26.0	26.1	24.8	25.0	25.6	24.2	25.3	0.0	-1.1	3.9E-02
ELMO2	25.0	25.3	25.2	25.4	25.0	24.9	25.1	24.9	25.2	25.5	25.2	25.7	25.2	25.2	25.4	26.4	26.6	26.2	24.9	26.3	25.2	26.3	0.0	-1.1	3.9E-02
TRIO	24.7	25.5	24.4	24.7	24.2	24.7	24.2	24.0	24.5	24.0	24.4	25.6	23.8	24.9	25.4	26.4	27.4	25.0	24.8	25.6	24.5	25.5	0.0	-1.0	4.0E-02
PFN2	28.3	28.0	28.3	27.8	28.4	28.6	28.6	28.2	28.2	28.2	28.5	28.1	29.0	28.5	28.8	29.3	29.2	29.4	29.4	29.9	28.3	29.3	0.0	-1.0	4.0E-02
SUMF2	25.9	25.4	25.3	25.5	25.4	25.3	25.5	25.2	25.7	25.7	25.5	26.4	25.7	25.9	26.4	26.5	26.5	26.1	27.1	27.5	25.5	26.5	0.0	-1.0	4.0E-02
FN3KRP	24.1	23.8	24.5	24.6	24.5	24.5	24.8	24.7	24.2	24.7	24.6	24.1	24.9	24.0	25.2	25.7	25.2	25.7	25.4	26.4	24.5	25.5	0.0	-1.0	4.1E-02
HARS	28.6	28.8	29.4	29.2	28.0	28.1	28.4	29.1	28.6	28.7	28.5	28.9	28.7	28.4	28.9	29.5	29.8	29.7	29.7	29.9	28.7	29.7	0.0	-1.0	4.1E-02
GBE1	25.7	25.6	26.3	26.0	25.8	26.2	25.7	25.7	25.5	25.5	26.1	25.6	26.3	25.8	26.5	26.5	27.6	26.9	26.9	26.8	25.8	26.8	0.0	-1.0	4.2E-02
ACP2	24.9	25.4	25.1	23.7	25.4	25.0	24.9	25.2	24.8	25.1	25.3	24.8	25.4	25.1	25.6	26.7	25.9	26.3	25.3	26.9	25.1	26.1	0.0	-1.0	4.2E-02
MVD	28.9	28.8	29.3	29.2	28.7	29.2	28.7	29.1	28.8	29.0	29.1	29.0	29.2	29.1	29.0	30.0	30.1	30.7	30.4	30.0	29.0	30.1	0.0	-1.0	4.2E-02
UFL1	26.4	26.3	26.7	27.3	27.1	27.0	27.0	26.8	26.7	26.4	26.6	26.6	27.3	26.4	27.3	28.4	28.1	27.5	27.3	28.0	26.7	27.8	0.0	-1.0	4.2E-02
CCDC90B	NaN	NaN	21.5	22.2	NaN	21.7	21.3	NaN	21.8	21.1	NaN	NaN	22.2	21.4	21.5	23.0	22.0	23.1	23.0	22.2	21.6	22.6	0.0	-1.0	4.2E-02
FAM127A	23.4	24.3	22.4	22.4	NaN	22.4	23.1	24.0	22.5	23.5	23.4	23.4	23.9	23.4	23.2	24.9	24.9	25.0	24.0	24.0	23.4	24.4	0.0	-1.0	4.3E-02
ARL6IP5	27.1	27.2	27.2	26.4	27.2	27.6	26.8	26.7	27.0	27.5	27.1	27.3	27.2	27.3	27.7	29.2	28.2	28.2	28.0	29.0	27.2	28.2	0.0	-1.0	4.3E-02
RABGAP1	25.6	25.8	25.5	25.6	25.2	25.1	25.9	25.6	25.3	26.2	25.7	25.3	25.8	25.9	26.1	27.2	26.7	27.3	26.3	26.6	25.6	26.6	0.0	-1.0	4.3E-02
PURB	25.7	24.3	25.8	26.9	24.2	24.8	25.8	25.2	25.0	25.9	25.8	25.3	25.9	25.4	25.9	26.3	26.3	27.9	26.9	27.0	25.6	26.6	0.0	-1.0	4.4E-02
SUCLG1	27.9	28.0	27.7	28.5	27.6	27.3	27.8	27.3	27.8	28.0	27.7	28.2	27.7	28.4	28.2	28.9	28.8	28.6	28.9	28.9	27.8	28.8	0.0	-1.0	4.4E-02
ITGAV	27.4	27.3	26.1	27.5	27.3	26.9	27.4	26.6	27.0	27.4	26.9	27.3	27.0	27.2	27.1	29.5	28.2	29.0	28.1	28.3	27.2	28.3	0.0	-1.0	4.4E-02
FAHD1	26.3	26.1	26.4	27.0	26.6	26.7	26.6	26.8	26.5	26.6	26.0	26.6	26.6	26.6	26.9	28.0	27.6	27.8	27.4	27.7	26.6	27.6	0.0	-1.0	4.4E-02
ACOX3	23.0	23.9	NaN	23.3	NaN	NaN	23.1	23.7	NaN	23.3	22.8	23.4	23.0	23.2	23.4	24.2	25.5	24.4	24.0	26.1	23.3	24.3	0.0	-1.0	4.5E-02
DCTN2	28.9	29.5	28.9	29.1	28.6	28.5	29.0	29.9	28.9	29.2	29.2	28.9	29.2	29.3	29.6	30.6	29.9	30.6	29.9	30.3	29.1	30.1	0.0	-1.0	4.5E-02
ZNF512	26.1	26.3	25.8	25.6	26.5	26.7	25.9	26.3	24.9	26.5	26.5	25.5	26.4	26.3	27.3	27.4	27.2	26.9	26.4	27.5	26.3	27.3	0.0	-1.0	4.5E-02
G6PD	29.4	29.7	29.9	29.5	29.5	29.7	29.6	29.3	29.5	29.6	29.8	29.4	30.1	29.8	29.9	30.4	30.3	30.9	30.9	30.9	29.6	30.6	0.0	-1.0	4.6E-02
PCYT2	25.6	25.6	25.5	26.1	26.0	26.7	25.8	25.4	25.3	25.7	25.8	25.4	25.5	25.9	25.7	26.6	26.6	26.9	27.0	26.7	25.7	26.7	0.0	-1.0	4.6E-02
SESTD1	22.4	22.8	23.1	23.4	22.9	24.1	23.1	22.8	23.2	23.8	23.2	23.6	21.9	22.9	23.9	24.2	24.7	24.0	23.7	24.3	23.1	24.1	0.0	-1.0	4.6E-02
MSN	32.2	32.2	32.3	32.3	32.1	32.1	32.8	32.5	32.1	32.5	32.6	32.1	32.7	32.5	32.6	33.8	33.3	33.5	32.9	33.3	32.3	33.3	0.0	-1.0	4.6E-02

AKT1S1	24.9	NaN	25.3	25.1	25.3	24.9	25.0	NaN	25.1	25.5	25.3	NaN	25.0	25.7	25.4	26.1	26.1	25.7	NaN	26.2	25.1	26.1	0.0	-1.0	4.6E-02
PLXNA1	28.0	29.2	27.6	28.2	28.1	30.5	27.2	27.4	27.4	29.2	28.0	29.6	27.4	27.7	28.4	29.3	28.8	30.1	29.1	28.9	28.0	29.0	0.0	-1.0	4.6E-02
SDCBP	27.2	26.6	27.4	27.0	26.4	26.9	27.2	27.0	27.0	27.6	27.3	27.2	27.3	27.1	27.4	28.5	28.4	27.9	27.2	29.3	27.1	28.1	0.0	-1.0	4.7E-02
ASPH	26.4	26.4	26.4	26.4	26.8	26.7	25.9	26.9	26.2	26.6	26.5	26.7	26.5	26.4	26.5	27.5	28.0	27.5	27.4	28.3	26.5	27.5	0.0	-1.0	4.7E-02
APPL2	23.6	23.4	23.7	NaN	NaN	NaN	NaN	22.4	23.0	24.3	23.4	24.8	23.2	22.5	24.0	27.7	24.3	26.1	24.6	24.2	23.4	24.4	0.0	-1.0	4.8E-02
H2AFY	29.9	30.5	30.3	29.8	30.3	30.1	29.8	30.2	30.3	30.4	30.7	30.5	30.6	30.4	31.2	32.0	31.4	31.2	30.9	31.3	30.3	31.3	0.0	-1.0	4.8E-02
UHRF1	27.1	27.3	27.0	27.3	27.5	27.3	27.0	27.2	27.0	27.6	27.5	27.3	26.9	27.2	28.5	27.9	28.3	28.4	28.2	27.2	27.2	28.2	0.0	-1.0	4.8E-02
GATAD2B	26.2	26.2	26.4	26.3	26.7	26.3	26.3	26.2	26.3	27.0	27.0	26.2	26.5	26.8	27.3	27.0	28.3	27.0	27.4	27.7	26.3	27.3	0.0	-1.0	4.8E-02
VPS35	28.6	28.6	29.0	28.8	28.5	28.6	28.7	28.8	28.7	28.8	28.8	28.9	29.2	28.8	29.0	30.2	29.9	30.0	29.5	29.7	28.8	29.8	0.0	-1.0	4.8E-02
NECAP2	25.2	25.3	25.7	25.1	24.1	24.7	25.2	25.1	25.4	25.8	25.5	24.9	25.4	25.1	25.7	26.1	26.2	25.9	26.3	26.3	25.2	26.2	0.0	-1.0	4.9E-02
CARS2	24.4	23.8	24.8	24.7	23.7	23.5	23.4	23.6	24.1	24.3	24.1	24.5	24.4	24.0	24.3	25.5	24.7	25.8	24.6	25.5	24.1	25.1	0.0	-1.0	4.9E-02
