# Supplementary material for: Reprogrammed Cells Display Distinct Proteomic Signatures Associated with Colony Morphology Variability
Source: Stem Cells Int. 2019 Nov 18;2019:8036035. doi: 10.1155/2019/8036035 (PMC6885794; doi:10.1155/2019/8036035)
Supplement: Supplementary 1 — Supplementary table 1: information about the hiPSC lines included in the study. Supplementary table 2: a list of proteins being more abundant in stable colony morphology group. Supplementary table 3: a list of proteins being more abundant in the unstable colony morphology group. Supplementary table 4: activated upstream regulators in the unstable colony morphology group. [file 8036035.f1.zip › supplementary table 4.pdf]

Supplement table 4: Activated upstream regulators in the transformed morphology group.

© 2000-2019 QIAGEN. All rights reserved.

Upstream Regulator	Molecule Type	Predicted Activation State	Activation z-score	p-value of overlap
TCF7L2	transcription regulator	Activated	3,706	0,0000207
CTGF	growth factor	Activated	3,145	0,000678
TGFB1	growth factor	Activated	3,066	1,46E-12
ETV5	transcription regulator	Activated	2,813	0,0000138
VCAN	other	Activated	2,621	0,154
TNF	cytokine	Activated	2,607	0,0000681
MYOD1	transcription regulator	Activated	2,587	0,159
IL4	cytokine	Activated	2,579	0,00012
TP53	transcription regulator	Activated	2,567	1,96E-22
SREBF1	transcription regulator	Activated	2,554	0,0000254
PRKCD	kinase	Activated	2,449	0,432
GATA6	transcription regulator	Activated	2,425	0,447
GH1	growth factor	Activated	2,395	0,0411
SREBF2	transcription regulator	Activated	2,373	0,0000459
TWIST1	transcription regulator	Activated	2,359	0,0118
Collagen type I	complex	Activated	2,298	0,0000826
PAX7	transcription regulator	Activated	2,236	0,0157
THRB	ligand-dependent nuclear receptor	Activated	2,236	0,00777
TWIST2	transcription regulator	Activated	2,234	0,00298
FGF10	growth factor	Activated	2,219	0,00406
NOTCH3	transcription regulator	Activated	2,219	0,0037
MAP2K1	kinase	Activated	2,216	0,0713
SNAI1	transcription regulator	Activated	2,208	0,000000242
PTK2	kinase	Activated	2,195	0,000869
VEGFA	growth factor	Activated	2,17	0,000678
OSM	cytokine	Activated	2,17	0,000238
ALDH2	enzyme	Activated	2,138	0,000000113
TGFB3	growth factor	Activated	2,126	0,0122
Tgf beta	group	Activated	2,098	0,0215
SPP1	cytokine	Activated	2,072	0,000000797
HNF4A	transcription regulator	Activated	2,065	4,12E-09
GLI1	transcription regulator	Activated	2,028	0,00165
TP73	transcription regulator	Activated	2,02	0,00787
LTA	cytokine	Activated	2	0,0356
Focal adhesion kinase	group	Activated	2	0,00905
EDN3	other	Activated	2	0,00241
SIRT2	transcription regulator	Activated	2	0,00163
CSNK2A1	kinase	Activated	2	0,00104
