# Supplementary figures and images for: Reprogrammed Cells Display Distinct Proteomic Signatures Associated with Colony Morphology Variability
Source: Stem Cells Int. 2019 Nov 18;2019:8036035. doi: 10.1155/2019/8036035 (PMC6885794; doi:10.1155/2019/8036035)

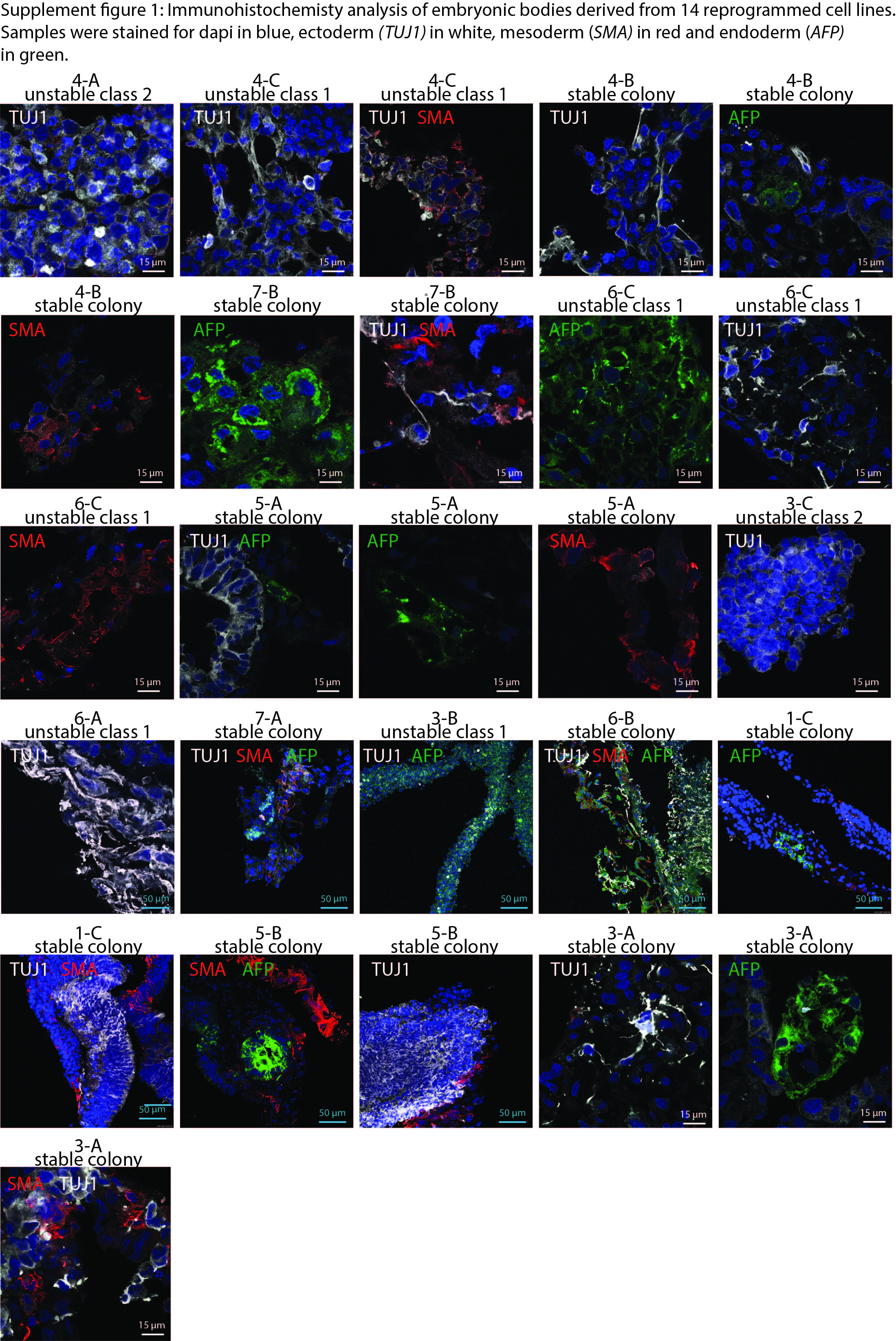

Supplement: Supplementary 2 — Supplementary figure 1: immunohistochemistry images of all embryonic bodies. [file 8036035.f2.jpg]
